# Cooperative Chiral Lewis Base/Palladium‐Catalyzed Asymmetric Syntheses of Methylene‐Containing δ‐Lactams

**DOI:** 10.1002/ejoc.202300982

**Published:** 2023-11-09

**Authors:** Paul Zebrowski, Uwe Monkowius, Mario Waser

**Affiliations:** ^1^ Institute of Organic Chemistry Johannes Kepler University Linz Altenbergerstrasse 69 4040 Linz Austria; ^2^ School of Education Chemistry Johannes Kepler University Linz Altenbergerstrasse 69 4040 Linz Austria

**Keywords:** asymmetric organocatalysis, Pd-catalysis, cyclizations, lactams, Lewis base catalysis

## Abstract

We herein report a two‐step approach for the enantioselective synthesis of novel chiral δ‐lactams. By using a cooperative chiral ITU/achiral Pd‐catalyst system, this protocol proceeds via an asymmetric α‐allylation of activated aryl esters first, followed by an acid‐mediated lactam formation. A variety of differently substituted products could be obtained with usually high levels of enantioselectivities and in reasonable yields (16 examples, up to 98 : 2 er and 73 % yield over two steps). In addition, further utilizations of the products via transformations of the exocyclic double bond were successfully carried out as well.

## Graphical abstract

An asymmetric protocol for the syntheses of novel δ‐lactams by utilizing a synergistic chiral isothiourea/achiral Pd‐catalyst combination for the α‐allylation of activated aryl esters followed by deprotection/cyclization has been successfully established.

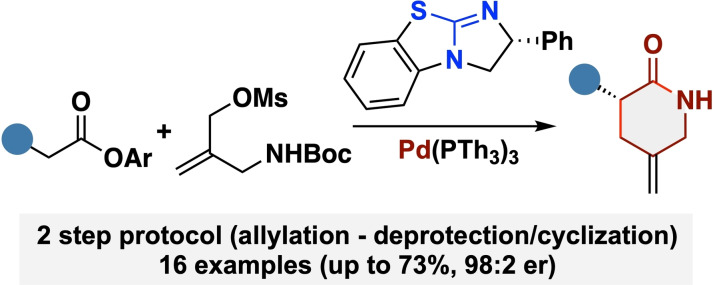

## Introduction

**Scheme 1:**
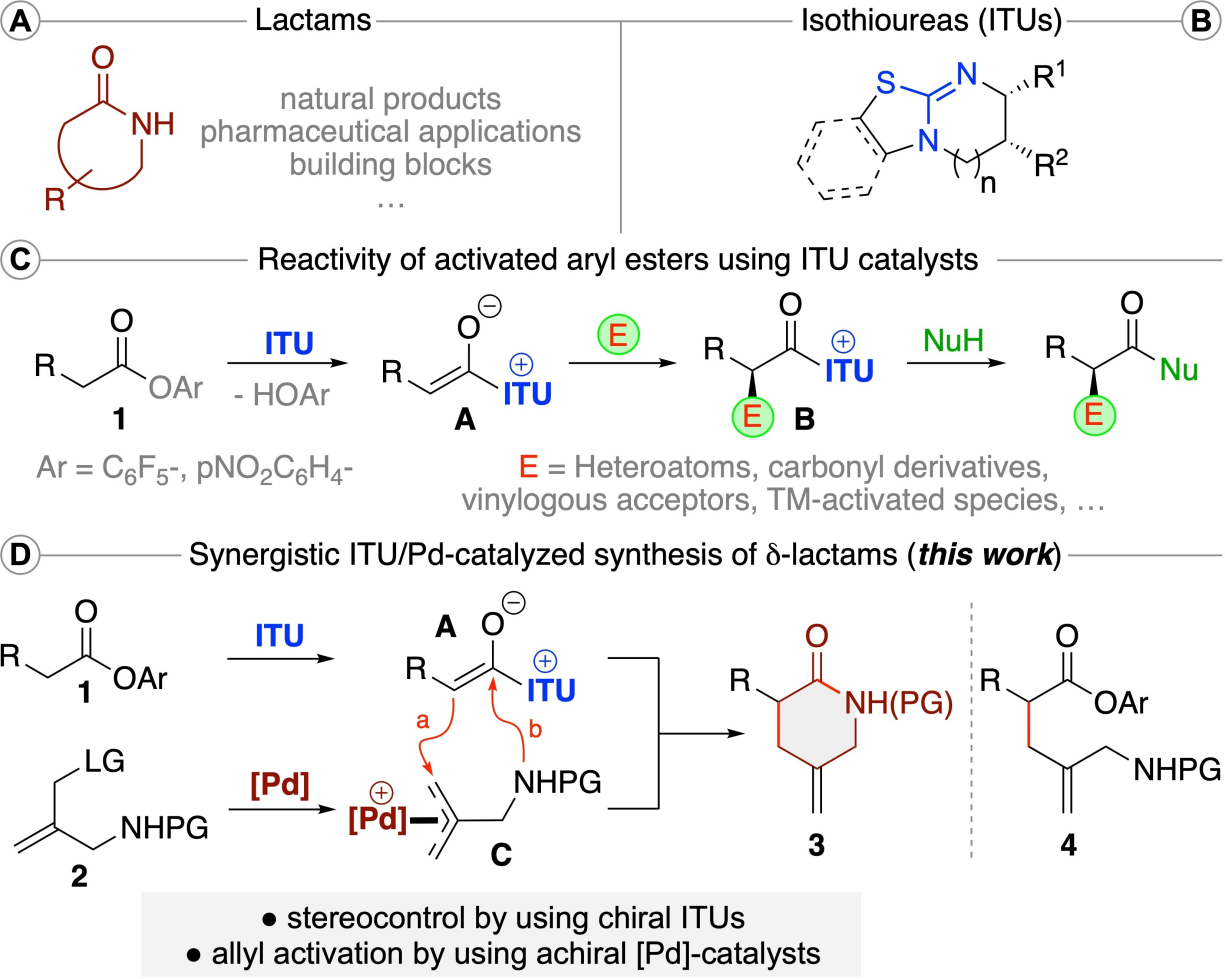
General structures of lactams (A) and isothioureas (B), the general reactivity of activated aryl esters **1** under ITU catalysis (C), and the herein investigated synergistic ITU/Pd‐catalyzed synthesis of δ‐lactams **3** (D).

Lactams are amongst the most prominent heterocyclic motives which are frequently found in natural products and biologically active molecules (Scheme 1A).^[1]^ In addition, they also serve as valuable building blocks for further manipulations (e. g. polymerizations),^[2]^ and thus it comes as no surprise that the introduction of synthesis strategies to access novel (chiral) lactams represents an important task.[^3^, ^4^] Our group has a long standing interest in the development of organocatalytic asymmetric 5–7‐membered ring heterocycle formations.^[5]^ Hereby we have become especially fascinated by the unique activation potential of chiral isothiourea (ITU) Lewis base organocatalysts (Scheme 1B).^[6]^ These easily accessible nucleophilic catalysts can facilitate a multitude of asymmetric α‐functionalization and cyclization reactions of activated carboxylic acid derivatives like electron‐deficient aryl esters **1** (Scheme 1C).[^7^, ^8^, ^9^, ^10^, ^11^, ^12^] Mechanistically, such transformations proceed via displacement of the OAr group by the ITU and subsequent α‐deprotonation to generate chiral C1 ammonium enolates **A** in situ. These can then add to different electrophiles in an enantioselective manner giving the catalyst‐bound products **B** which finally react with nucleophiles (like the initially cleaved‐off HOAr^[7c]^ group or an external nucleophile) either in a cyclization approach or in a bimolecular manner by releasing the catalyst again.[^8^, ^9^] Besides electrophilic heteroatom transfer reagents, carbonyl derivatives, and various vinylogous acceptors, the last years have also witnessed the introduction of a variety of impressive synergistic approaches^[10]^ where complementary catalysis principles, i. e. transition metal (TM) catalysts, were employed to generate highly reactive electrophilic species in situ.[^11^, ^12^]

Inspired by Snaddon's seminal contribution demonstrating the potential of such synergistic Lewis base/Pd‐catalysis approaches for asymmetric allylations of C1 ammonium enolates,^[11]^ a variety of spectacular conceptually similar transformations have been introduced by numerous research groups over the course of the last years.^[12]^

Based on these recent advancements in the field of synergistic Lewis base/transition metal catalysis,[^10^, ^11^, ^12^] our own focus on asymmetric ITU catalysis and heterocycle forming reactions,[^5^, ^9^] and the general value of developing novel lactam‐forming approaches, we now became interested in utilizing cooperative Lewis base/TM‐catalysis to access novel δ‐lactams.^[13]^ We rationalized that it should be possible to utilize esters **1** and activated allylamine compounds **2** under cooperative ITU/Pd catalysis to access δ‐lactams **3** in an asymmetric fashion (Scheme 1D). More specifically, while the ITU activates ester **1** by forming the established chiral C1 ammonium enolate species **A**, Pd‐catalysis will allow for the activation of compounds **2** by forming highly electrophilic π‐allyl‐Pd species **C**.^[14]^ These two in situ formed reagents should be prone to react via a two‐step allylation (a) – cyclization (b) sequence to access the targeted δ‐lactams **3** either directly, or, depending on the reactivity of the N‐protected amine group, via formation of the intermediate allylation product **4** first, which can then undergo deprotection and cyclization towards **3** in a distinct step. It should be noted that analogous scaffolds containing a tetra‐substituted α‐stereogenic center were recently accessed enantioselectively by utilizing deconjugated butenolides^[15]^ or azlactones^16]^ under asymmetric Pd‐catalysis.

## Results and Discussion

We started by carrying out a systematic screening of different ITUs, Pd‐sources and ligands, allylation reagents **2**, and bases and solvents for the synthesis of the parent lactam **3 a**.^[17]^ Right from the beginning we found it necessary to carry out the reaction in a two‐step fashion, where the first synergistic catalytic step gave the α‐allylation product **4 a** (ITU turnover was thus achieved by phenolate rebinding), followed by an acid‐mediated quantitative Boc‐deprotection/lactamization sequence delivering the targeted lactam **3 a**.

Amongst a variety of different ITU catalysts and allylation reagents tested, we rapidly identified **BTM** ^[18]^ as the catalyst of choice when using the allylation reagent **2 a** in THF in combination with Hünig's Base (DIPEA).^[17]^ With these parent conditions at hand, we carried out a screening of different Pd‐sources and ligands next (Table 1 gives a condensed overview about the most significant results obtained hereby).^[17]^ In general, the allylation step required at least one day for reasonable amount of product **4 a** formation. Unfortunately however, we obtained rather low yields accompanied with significant amounts of mesylate **2 a** decomposition and formation of other side products in our initial experiments when using Pd_2_(dba)_3_ in combination with different phosphine ligands only (entries 1–5; Xantphos, P(2‐Fu)_3_ and P(2‐Th)_3_ were successfully used for similar applications before,[^11^, ^19^] L1 and DTBPF were tested to see if other ligand classes may be suited for this application either). Thus, despite the promising initial levels of enantioselectivities obtained herein, we decided to screen more advanced Pd complexes in order to improve product formation. While XantphosPdG3 did not allow us to improve the low allylation yield (entry 6), the use of Pd(PTh_3_)_3_ (which was also the system of choice for conceptually similar allylations recently^[19]^) was found to be much more promising. By employing 10 mol % of this Pd catalyst in combination with 20 mol % BTM the allylation proceeded in a much cleaner manner (67 % for the first step), delivering the lactam **3 a** with an overall NMR yield of 65 % and a high enantioselectivity of 96 : 4 er (entry 7; lower loadings of both catalysts were tested but resulted in reduced yields and enantioselectivities^[17]^). Further optimization of concentration and stoichiometry (entries 8–11) showed that slightly more diluted conditions and a small excess of ester **1 a** ^[20]^ allow for a good isolated yield of 64 % over both steps on 1 mmol scale with high enantioselectivity (97 : 3 er).

**Table 1:** Optimization of reaction conditions for the synthesis of **3 a**.^[a]^

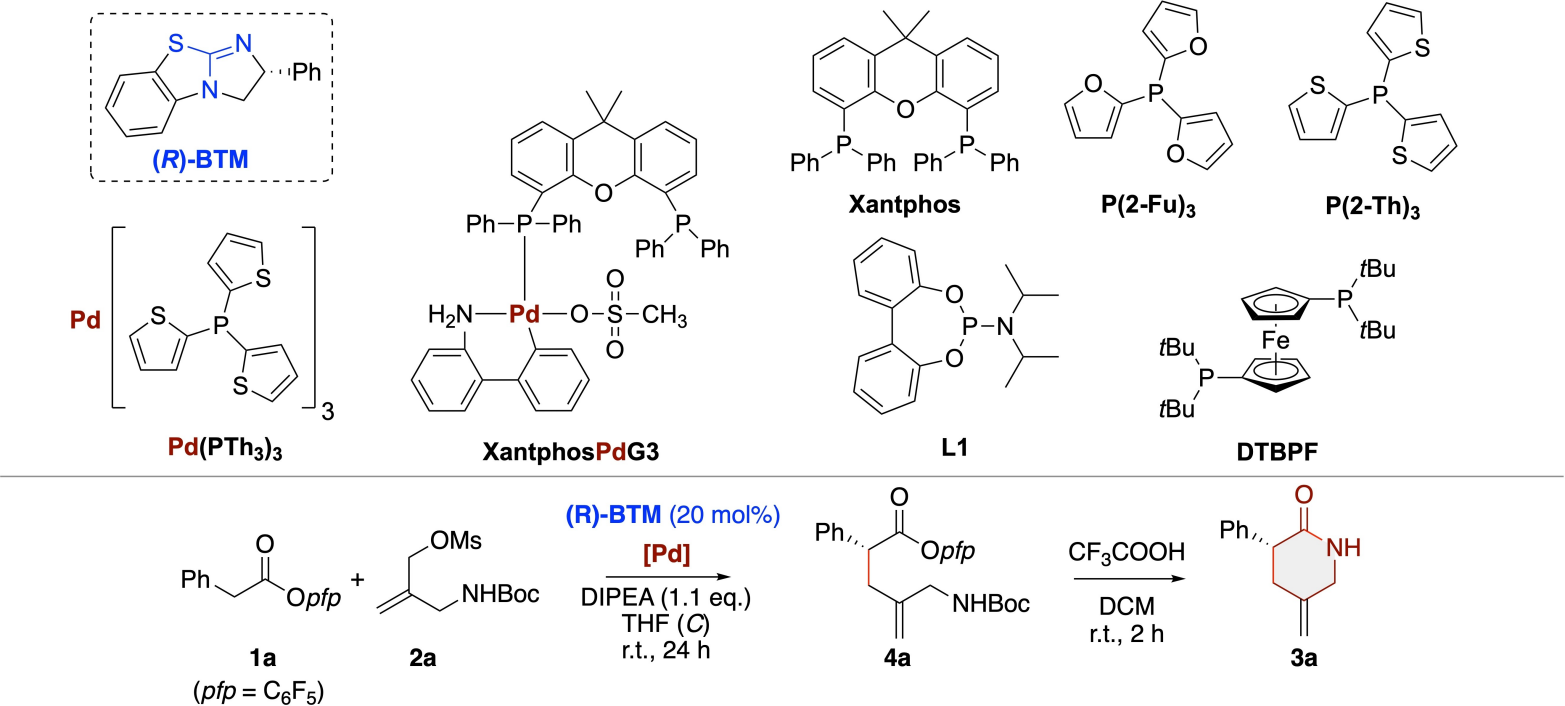							
Entry	[**Pd**]	Ligand	**2 a** [equiv.]	Conc.	**4 a** [%]^[b]^	**3 a** [%]^[c]^	er (**3 a**)^[d]^
1	Pd_2_(dba)_3_.CHCl_3_ (5 mol %)	Xantphos (20 mol %)	1.2	0.1 M	22	21	86 : 14
2	Pd_2_(dba)_3_.CHCl_3_ (5 mol %)	L1 (20 mol %)	1.2	0.1 M	11	11	61 : 39
3	Pd_2_(dba)_3_.CHCl_3_ (5 mol %)	DTBPF (20 mol %)	1.2	0.1 M	23	22	65 : 35
4	Pd_2_(dba)_3_.CHCl_3_ (5 mol %)	P(2‐Fu)_3_ (20 mol %)	1.2	0.1 M	27	25	90 : 10
5	Pd_2_(dba)_3_.CHCl_3_ (5 mol %)	P(2‐Th)_3_ (20 mol %)	1.2	0.1 M	31	30	93 : 7
6	XantphosPdG3 (10 mol %)	–	1.2	0.1 M	11	10	60 : 40
7	Pd(PTh_3_)_3_ (10 mol %)	–	1.2	0.1 M	67	65	96 : 4
8	Pd(PTh_3_)_3_ (10 mol %)	–	1.2	0.05 M	65	65	97 : 3
9	Pd(PTh_3_)_3_ (10 mol %)	–	1.2	0.025 M	45	44	97 : 3
10	Pd(PTh_3_)_3_ (10 mol %)	–	0.8	0.05	72	70	97 : 3
11	Pd(PTh_3_)_3_ (10 mol %)	–	0.8	0.05	68^[e]^	64^[e]^	97 : 3

**Scheme 2:**
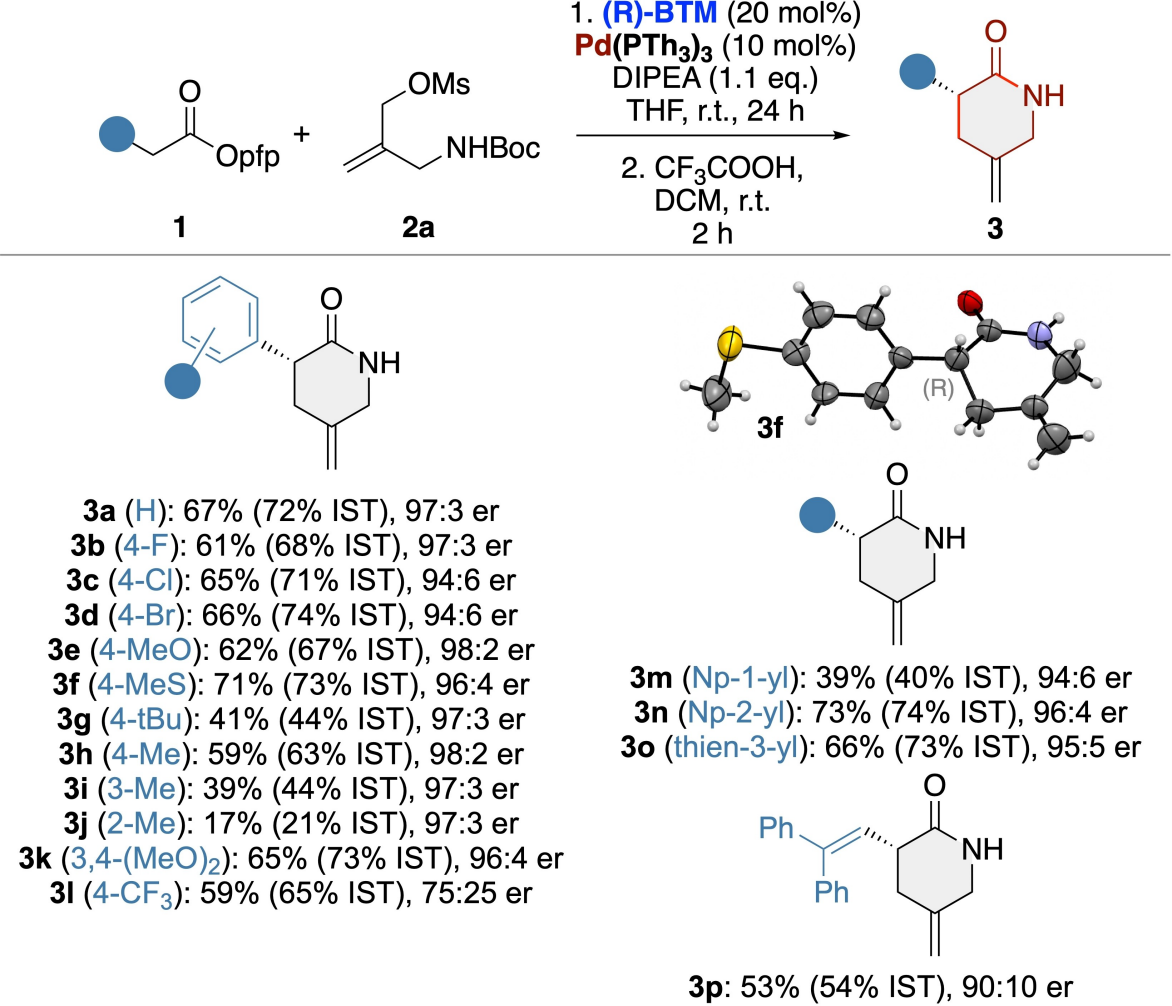
Asymmetric application scope (isolated and NMR (IST) yields over both steps).

[a] Unless otherwise stated, all reactions were carried out in two steps by first stirring **1 a** (0.1 mmol), the indicated amount of **2 a**, DIPEA (1.1 eq.), (R)‐BTM (20 mol %), and the given Pd system in THF for 24 h at r.t. giving **4 a**. Intermediate **4 a** was then stirred in a mixture of CF_3_COOH (50 eq.) and DCM for 2 h at r.t. giving **3 a**. [b] NMR yields using o‐xylene as an internal standard (IST). [c] NMR yields over both steps using o‐xylene as an internal standard. [d] Determined by HPLC using a chiral stationary phase (absolute configuration was assigned in analogy to derivative **3 f** (Scheme 2)). [e] Isolated yields on 1 mmol scale.

Having identified suitable two‐step conditions for the enantioselective synthesis of lactam **3 a**, we next investigated the application scope of this protocol (Scheme 2). In general, a variety of differently substituted lactams **3** were all accessible with comparably high enantioselectivities. The only case where we observed a significantly reduced selectivity was for the CF_3_‐containing product **3 l**. However, the nature of the substituents showed some impact on conversion and yield and especially derivatives with ortho‐substituted aryl groups showed lower conversion rates and gave lower yields only (**3 j**, **m**). Gratifyingly, we were able to analyze enantioenriched **3 f** by X‐Ray diffraction analysis,^[21]^ which allowed us to assign the absolute configuration being (R) for this derivative and assignment of the other products was done in analogy (it should be stated that this sense of induction is in full accordance with Snaddon's previous allylation reports when using BTM as a catalyst[^11^, ^19^])

**Scheme 3:**
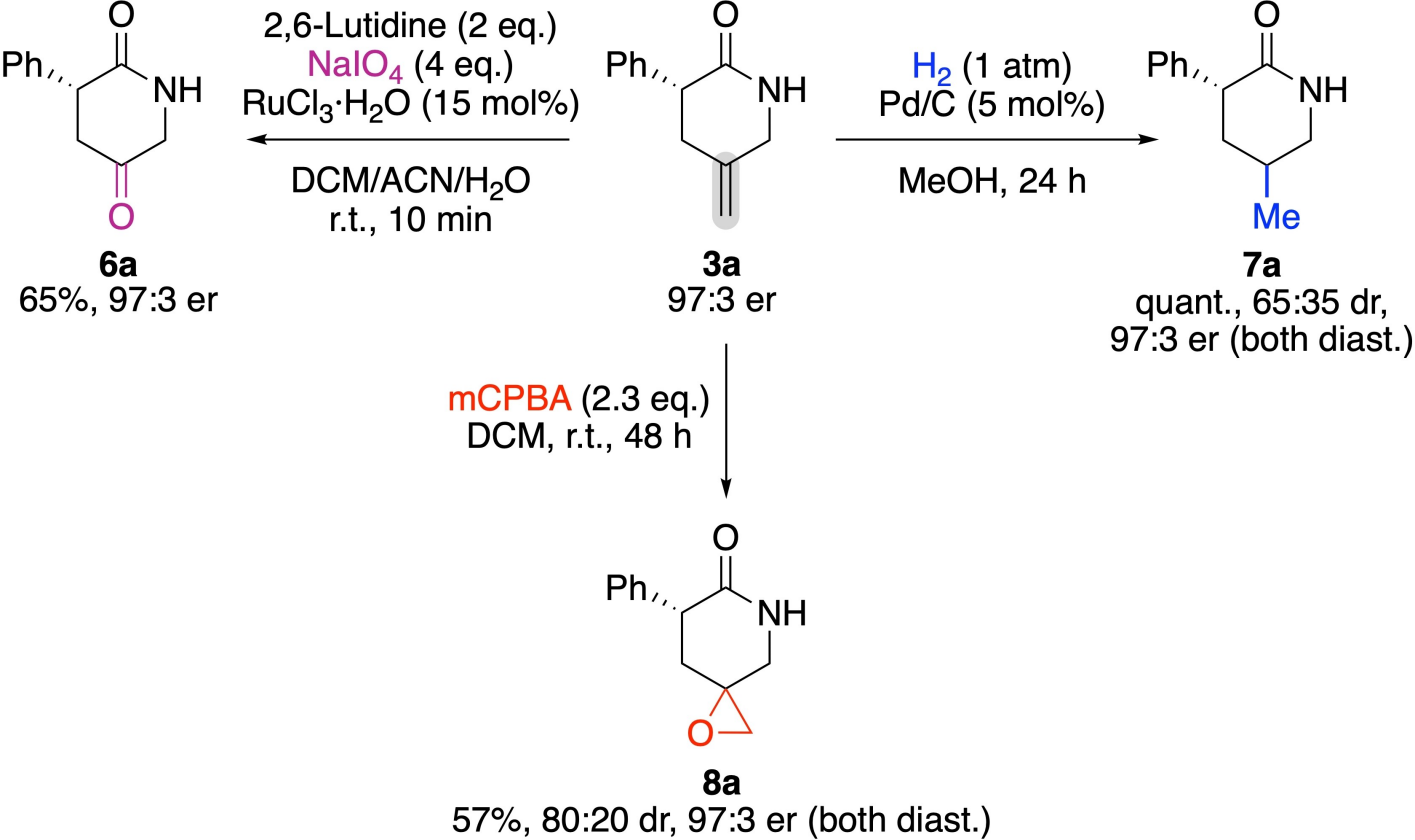
Further manipulations of the exocyclic double bond.

Finally, we also investigated the suitability of **3 a** for further manipulations of the exocyclic double bond. As outlined in Scheme 3, this double bond could either be hydrogenated (product **7 a**), oxidatively cleaved (product **6 a**), or epoxidized (product **8 a**) under standard conditions without any erosion of enantiopurity. Interestingly, a classical N‐Boc‐protection of **3 a** using Boc_2_O, DMAP, and Et_3_N lead to pronounced racemization of the stereogenic center however.

## Conclusions

A two‐step protocol for the asymmetric syntheses of chiral δ‐lactams **3** via reaction of activated aryl esters **1** with allylamine reagent **2 a** was developed. This transformation relies on the synergistic combination of chiral isothiourea catalysis (giving a chiral C1 ammonium enolate **A**) and achiral Pd‐catalysis (resulting in the formation of an electrophilic π‐allyl‐Pd species **C**) for the initial allylation step, which yields the intermediate allylation products **4** first. These compounds then underwent a quantitative acid‐mediated deprotection‐cyclization delivering the final δ‐lactams **3** in yields up to 73 % and up to 98 : 2 er. Further manipulations of the exocyclic double bond of products **3**, like hydrogenations, oxidative cleavage, and epoxidation, were carried out under established standard conditions and proceeded with full retention of enantiopurity.

## Experimental Section

*General procedure for the syntheses of compounds **3***: A flame‐dried Schlenk tube equipped with a magnetic stirring bar was charged with Pd(PTh_3_)_3_ (9.5 mg, 10 mol %). The flask was evacuated and backfilled with Ar (3x). Dry and degassed THF (2 mL, 0.05 M with respect to **2 a**) was added and the mixture was stirred for 5 minutes giving a clear yellow solution. Pfp ester **1** (125 μmol, 1.25 equiv), DIPEA (18.7 μL, 110 μmol, 1.1 equiv), (*R*)‐BTM (5.0 mg, 20 mol %) and mesylate **2 a** (26.5 mg, 100 μmol, 1 equiv) were added sequentially and the reaction was stirred for 24 h under Ar. The mixture was concentrated under reduced pressure and filtered through a short pad of silica gel (DCM). Evaporation of the solvent the gave crude allylation products **4**, which were used without further purification. The crude product was dissolved in DCM (1 mL) and TFA (400 μL) was added. The reaction was stirred for 2 h and concentrated followed by 2–3 cycles of co‐evaporation with DCM to remove residual TFA. Filtration through a short pad of basic Al_2_O_3_ (DCM/MeOH=20/1) and concentration under reduced pressure gave the crude products **3**, which were purified by column chromatography (silica gel, 2.5 vol % MeOH in DCM).

*Analytical details for the parent compound **3 a***: m.p.=80–83 °C. TLC (silica gel, 2.5 vol % MeOH in DCM): R_f_=0.20 (KMnO_4_). [α]_D_^19^ (*c* 0.96, CHCl_3_)=−51.7°. ^1^H NMR (500 MHz, δ, CDCl_3_, 298 K): 7.34–7.31 (m, 2H), 7.26–7.23 (m, 3H), 6.56 (bs, 1H), 5.00 (s, 1H), 4.90 (s, 1H), 4.06–4.00 (m, 2H), 3.71 (dd, *J*=7.9, 5.9 Hz, 1H), 2.84 (dd, *J*=13.9, 5.9 Hz, 1H), 2.69 (dd, *J*=13.9, 7.9 Hz, 1H). ^13^C NMR (75 MHz, δ, CDCl_3_, 298 K): 173.3, 139.8, 137.7, 128.7, 128.3, 127.1, 112.3, 48.5, 47.8, 37.6. HPLC (YMC Chiral ART Cellulose‐SB, eluent: *n*‐hexane:*i*‐PrOH=4/1, 1.0 mL⋅min‐1, 10 °C, λ=210 nm) retention times: t_*major*_=13.8 min, t_*minor*_=23.9 min. HRMS (ESI): calcd *m/z* for C_12_H_14_NO^+^: 188.1070 [*M*+H]+; found: 188.1071.

## Supporting Information

Experimental procedures, screening details, and analytical characterization of all new compounds are provided in the online Supporting Information. Additional references were cited within the Supporting Information.[^22^, ^23^]

## Conflict of interest

The authors declare no conflict of interest.

1

## Supporting information

As a service to our authors and readers, this journal provides supporting information supplied by the authors. Such materials are peer reviewed and may be re‐organized for online delivery, but are not copy‐edited or typeset. Technical support issues arising from supporting information (other than missing files) should be addressed to the authors.

Supporting Information

## Data Availability

The data that support the findings of this study are available in the supplementary material of this article.
