# Supplementary material for: Cooperative Chiral Lewis Base/Palladium‐Catalyzed Asymmetric Syntheses of Methylene‐Containing δ‐Lactams
Source: European J Org Chem. 2023 Nov 9;26(45):e202300982. doi: 10.1002/ejoc.202300982 (PMC11005102; doi:10.1002/ejoc.202300982)

European Journal of Organic Chemistry

Supporting Information

Cooperative Chiral Lewis Base/Palladium-Catalyzed Asymmetric Syntheses of Methylene-Containing δ-Lactams

Paul Zebrowski, Uwe Monkowius, and Mario Waser*

List of Contents

1. General Information ... 1
2. Syntheses of Starting Material ... 2
   - 2.1. Catalysts and Ligands ... 2
   - 2.2. Pfp-Esters **1** ... 3
   - 2.3. Allylation Reagents **2** ... 3
     - 2.3.1. Alcohol **11** ... 3
     - 2.3.2. Mesylate **2a** ... 4
     - 2.3.3. Acetate **2b** ... 4
     - 2.3.4. Methyl Carbonate **2c** ... 5
     - 2.3.5. Phosphate **2d** ... 5
     - 2.3.6. Pivalate **2e** ... 6
3. Reaction Optimization – Further Conditions Tested ... 7
   - 3.1. Lewis Base Screening ... 7
   - 3.2. Alternative Solvent Screening ... 7
   - 3.3. Alternative Electrophile Screening ... 8
   - 3.4. Alternative Base Screening ... 8
   - 3.5. Alternative Catalyst Loading Screening ... 9
   - 3.6. Alternative Temperature Screening ... 9
   - 3.7. Time Study ... 10
4. Syntheses and Analytical Details of Targets **3**, **4**, **6**, **7** and **8** ... 11
   - 4.1. Allylation – Deprotection/Cyclization Protocol ... 11
   - 4.2. Acyclic Allylated Ester Intermediate **4a** ... 17
   - 4.3. Further Transformations and Products ... 17
     - 4.3.1. Oxidative Cleavage of **3a** ... 17
     - 4.3.2. Double Bond Hydrogenation of **3a** ... 18
     - 4.3.3. Epoxidation of **3a** ... 19
5. Single Crystal X-Ray Diffraction ... 20
6. Copies of Product NMR Spectra ... 21
7. Copies of HPLC Chromatograms ... 54
8. HRMS Data ... 74

1. General Information

^{1}H-, ^{13}C-, ^{19}F- and ^{31}P-NMR spectra were recorded on a Bruker Avance III 300 MHz spectrometer with a broad band observe probe and a sample changer for 16 samples, and on a Bruker Avance DRX 500 MHz spectrometer with an Ascend magnet and TCI cryoprobe, which are both property of the Austro-Czech NMR-Research Center "RERI-uasb". NMR spectra were referenced on the solvent peak and chemical shifts are given in ppm.

High resolution mass spectra were obtained using a Thermo Scientific LTQ Orbitrap XL system with an Ion Max API Source. Analyses were made in the positive ionization mode if not otherwise stated. Purine (exact mass for $[M+H]^+$ = 121.050873) and 1,2,3,4,5,6-hexakis(2,2,3,3-tetrafluoropropoxy)-1,3,5,2,4,6-triazatriphosphinane (exact mass for $[M+H]^+$ = 922.009798) were used for internal mass calibration.

HPLC was performed using a Thermo Scientific Dionex Ultimate 3000 system with a CHIRALPAK OD-H, CHIRAL ART Amylose-SA or Cellulose-SB (250 × 4.6 mm, 5 µm) chiral stationary phase. Optical rotations were recorded on a Schmidt + Haensch Polarimeter Model UniPol L1000 at 589 nm. $[\alpha]_D^T$ values are listed in deg·cm^3·g^{-1}·dm^{-1}, concentration *c* is given in g/100 mL.

Preparative column chromatography was carried out using Davisil 60 Å 70–200 µm silica gel. (Preparative) Thin layer chromatography was performed using Machery-Nagel precoated aluminum sheets (ALUGRAM Xtra SIL G UV_{254}) and glass plates (SIL G UV_{254}). TLC plates were visualized by irradiation with 254 nm UV light or using a staining solution ($KMnO_4$) followed by heating.

Dry solvents were obtained from a MBraun-SPS-800 solvent purification system, degassed by freeze-pump-thaw technique and immediately used. All reactions were carried out under argon atmosphere, unless stated otherwise.

2. Syntheses of Starting Material

2.1. Catalysts and Ligands

Chiral isothiourea catalyst **(*R*)-Benzotetramisole** [(*R*)-BTM, CAS: 885051-07-0] was obtained from TCI and used without any purification. Palladium catalyst **$Pd(PTh_3)_3$** was prepared following the literature procedure of Bo and co-workers[1]. Achiral ITU catalyst **3,4-Dihydro-2*H*-9-thia-1,4a-diazafluorene** was used for the synthesis of racemic HPLC standards and prepared following the procedures of Okamoto and Kobayashi[2].

DTBPF [CAS: 84680-95-5] was obtained from BLDpharm and used without any purification.

(*S*)-Homobenzotetramisole [(*S*)-HBTM, CAS: 1015248-96-0], **$Pd_2(dba)_3$** [CAS: 51364-51-3], **$P(2\text{-}Fu)_3$** [CAS: 5518-52-5], **Xantphos** [CAS: 161265-03-8] and **XantPhos Pd G3** [CAS: 1445085-97-1] were obtained from Sigma-Aldrich. With exception of $Pd_2(dba)_3$, every compound was used without any purification. The palladium complex was purified by recrystallization from $CHCl_3$, following the literature procedure of Ananikov and Zalesskiy[3].

(+)-HyperBTM [CAS: 1203507-02-1] was obtained from Santa Cruz Biotechnology and used without any purification.

Phosphine ligand **L1** was prepared following the literature procedure of Harrity and co-workers[4].

$P(2\text{-}Th)_3$ [CAS: 24171-89-9] was obtained from Alfa Aesar and purified by column chromatography (*n*-heptane/EtOAc).

(-)-Tetramisole hydrochloride [CAS: 16595-80-5] was obtained from Fluorochem and converted into its free base *via* aqueous extraction (sat. Na_2CO_3/DCM).

---

[1] W. Li, Y. Han, B. Li, C. Liu and Z. Bo, *J. Polym. Sci. A Polym. Chem.,* **2008,** *46,* 4556–4563.
[2] M. Kobayashi and S. Okamoto, *Tetrahedron Lett.,* **2006,** *47,* 4347–4350.
[3] S. S. Zalesskiy and V. P. Ananikov, *Organometallics,* **2012,** *31,* 2302–2309.
[4] V. García-Vázquez, L. Hoteite, C. P. Lakeland, D. W. Watson and J. P. A. Harrity, *Org. Lett.,* **2021,** *23,* 2811–2815.

2.2. Pfp-Esters 1

Esters **1a-o** were prepared using established procedures[5]. Compounds **1f** and **1p** were used in literature[6], but (to best of our knowledge) have not been characterized before.

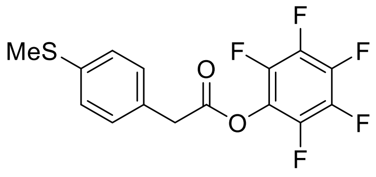

1f

Perfluorophenyl 2-(4-(methylthio)phenyl)acetate (1f): white solid; m.p. = 77–78 °C. TLC (silica gel, *n*-heptane/EtOAc = 20/1): R_f = 0.31 (UV). ^{1}H NMR (300 MHz, *δ*, $CDCl_3$, 298 K): 7.27 (s, 4H), 3.92 (s, 2H), 2.49 (s, 3H). ^{13}C NMR (75 MHz, *δ*, $CDCl_3$, 298 K): 167.5, 138.5, 129.8, 128.8, 127.0, 39.8, 15.9. ^{19}F NMR (282 MHz, δ, $CDCl_3$, 298 K): -152.5 – -152.6 (m, 2F), 157.8 (t, *J* = 21.8 Hz, 1F), -162.1 – -162.3 (m, 2F). HRMS (ESI): calcd *m/z* for $C_{15}H_{13}F_5NO_2S^+$: 366.0582 [*M*+NH_4]$^+$; found: 366.0578.

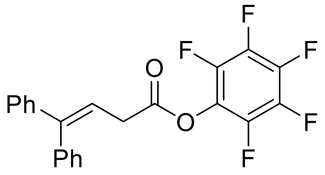

1p

Perfluorophenyl 4,4-diphenylbut-3-enoate (1p)[7]: white solid; m.p. = 94–95 °C. TLC (silica gel, *n*-heptane /EtOAc = 20/1): R_f = 0.48 (UV). ^{1}H NMR (300 MHz, *δ*, $CDCl_3$, 298 K): 7.46–7.19 (m, 10H), 6.29 (t, *J* = 7.4 Hz, 1H), 3.50 (d, *J* = 7.4 Hz, 2H). ^{13}C NMR (75 MHz, *δ*, $CDCl_3$, 298 K): 168.0, 146.8, 141.5, 138.9, 129.7, 128.8, 128.4, 128.0, 127.9, 127.6, 117.9, 34.6. ^{19}F NMR (282 MHz, δ, $CDCl_3$, 298 K): -152.6 – -152.7 (m, 2F), -157.9 (t, *J* = 21.6 Hz, 1F), -162.1 – -162.3 (m, 2F). HRMS (ESI): calcd *m/z* for $C_{22}H_{14}F_5O_2^+$: 405.0908 [*M*+H]$^+$; found: 405.0914.

2.3. Allylation Reagents 2

2.3.1. Alcohol 11

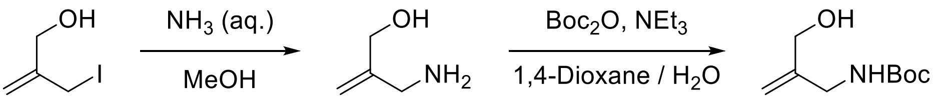

9 **10** **11**

2-(Iodomethyl)prop-2-en-1-ol (**9**)[4] (0.98 g, 4.95 mmol) was dissolved in MeOH (2.8 mL, 1.8 M with respect to **9**) and added dropwise to an ice-cooled solution of 28% NH_3 in H_2O (16.5 mL). The reaction was stirred for 1 h while gradually warming it up to room temperature. Evaporation of the solvent gave the crude amine **10**, which was used in the next step without any purification. The crude amine was dissolved in 1,4-dioxane/H_2O (10.8 mL, 5/1.5), cooled in an ice-bath and NEt_3 (1.3 mL, 9.4 mmol,

[4] V. García-Vázquez, L. Hoteite, C. P. Lakeland, D. W. Watson and J. P. A. Harrity, *Org. Lett.*, **2021,** *23,* 2811–2815.
[5] C. McLaughlin, A. M. Z. Slawin and A. D. Smith, *Angew. Chem. Int. Ed.*, **2019,** *58,* 15111–15119.
[6] a) M. Zhu, P. Wang, Q. Zhang, W. Tang and W. Zi, *Angew. Chem. Int. Ed.*, **2022,** *61,* e202207621; b) Q. Wang, T. Fan and J. Song, *Org. Lett.*, **2023,** *25,* 1246–1251.
[7] a) J.-J. Zhang, C.-S. Yan, Y. Peng, Z.-B. Luo, X.-B. Xu and Y.-W. Wang, *Org. Biomol. Chem.*, **2013,** *11,* 2498–2513; b) M.-C. Fu, R. Shang, W.-M. Cheng and Y. Fu, *Chemistry,* **2017,** *23,* 8818–8822.

1.9 equiv) and Boc_2O (1.18 g, 5.4 mmol, 1.1 equiv) were added drop/portion wise. The mixture was stirred for 24 h at room temperature. Aqueous work-up (H_2O/DCM) and purification by column chromatography (silica gel, *n*-heptane/EtOAc = 4/1→1/1) gave *N*-Boc protected amine **11** as a colorless oil in 59% yield (0.55 g, 2.94 mmol). The analytical data of **11** matched those reported in literature[8].

2.3.2. Mesylate 2a

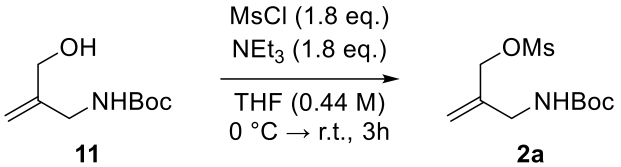

11 **2a**

Alcohol **11** (0.33 g, 1.76 mmol) was dissolved in dry THF (4 mL, 0.44 M with respect to **11**) and cooled in an ice-bath under stirring. NEt_3 (0.45 mL, 3.23 mmol, 1.8 equiv) and methanesulfonyl chloride (0.25 mL, 3.23 mmol, 1.8 equiv) were added dropwise, whereupon a white precipitate formed. The white suspension was vigorously stirred for 3 h while slowly warming it up to room temperature. The reaction was quenched with 1N HCl and the aqueous phase was extracted with EtOAc (3x). The combined organic extracts were washed with brine until neutral, dried with anhydrous Na_2SO_4, filtered over cotton, and concentrated under reduced pressure. Purification by column chromatography (silica gel, *n*-heptane/EtOAc = 2/1→1/1) gave allylic mesylate **2a** as a colorless oil in 98% yield (0.46 g, 1.73 mmol), which solidified upon storage in a refrigerator.

2-(((*tert*-Butoxycarbonyl)amino)methyl)allyl methanesulfonate (2a): white solid; m.p. = 52–55 °C. TLC (silica gel, *n*-heptane/EtOAc = 1/1): R_f = 0.32 ($KMnO_4$). ^1H NMR (300 MHz, δ, $CDCl_3$, 298 K): 5.30 (s, 1H), 5.28 (s, 1H), 4.77 (bs, 1H), 4.71 (s, 2H), 3.82 (d, J = 5.2 Hz, 2H), 3.04 (s, 3H), 1.44 (s, 9H). ^{13}C NMR (75 MHz, δ, $CDCl_3$, 298 K): 155.9, 139.6, 117.4, 80.0, 70.5, 42.6, 38.1, 28.5. HRMS (ESI): calcd *m/z* for $C_{10}H_{19}NNaO_5S^+$: 288.0876 $[M+Na]^+$; found: 288.0878.

2.3.3. Acetate 2b

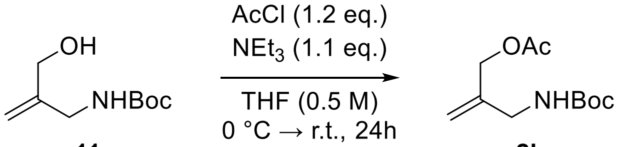

11 **2b**

Acetate **2b** was synthesized in analogy to **2a**. Alcohol **11** (0.14 g, 0.75 mmol), dissolved in dry THF (1.6 mL, 0.5 M) was treated with NEt_3 (0.11 mL, 0.79 mmol, 1.1 equiv) and acetyl chloride (62 µL, 0.87 mmol, 1.2 equiv). Aqueous work-up (sat. NH_4Cl, Et_2O and brine) followed by column

[8] Y. Komatsu, R. Watanabe, H. Ikishima, K. Nakano, Y. Ichikawa and H. Kotsuki, *Org. Biomol. Chem.*, **2012,** *10,* 2993–3001.

chromatography (silica gel, *n*-heptane/EtOAc = 4/1→1/1) gave allylic acetate **2b** as a colorless oil in 47% yield (80 mg, 0.35 mmol).

2-(((*tert*-Butoxycarbonyl)amino)methyl)allyl acetate (2b): colorless oil; TLC (silica gel, *n*-heptane/EtOAc = 1/1): R_f = 0.25 ($KMnO_4$). ^{1}H NMR (300 MHz, *δ*, $CDCl_3$, 298 K): 5.14–5.12 (m, 2H), 4.74 (bs, 1H), 4.56 (s, 2H), 3.76 (d, *J* = 5.5 Hz, 2H), 2.07 (s, 3H), 1.43 (s, 9H). ^{13}C NMR (75 MHz, *δ*, $CDCl_3$, 298 K): 170.8, 155.9, 141.2, 114.3, 79.7, 65.3, 43.0, 28.5, 21.0. HRMS (ESI): calcd *m/z* for $C_{11}H_{19}NNaO_4^+$: 252.1206 [*M*+Na]$^+$; found: 252.1208.

2.3.4. Methyl Carbonate 2c

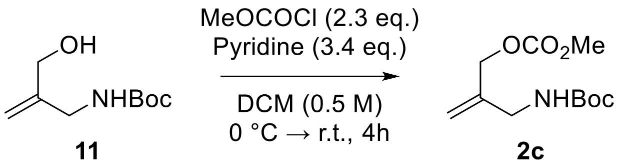

Alcohol **11** (0.18 g, 0.96 mmol) was dissolved in dry DCM (2 mL, 0.5 M with respect to **11**) and cooled in an ice-bath under stirring. Pyridine (0.26 mL, 3.23 mmol, 3.4 equiv) and methyl chloroformate (0.17 mL, 2.20 mmol, 2.3 equiv) were added dropwise. The reaction was stirred for 4 h while warming up to room temperature. The white suspension was quenched with brine and the phases were separated. The organic phase was washed with brine (1x) and deionized water (3x), dried with anhydrous Na_2SO_4, filtered over cotton, and concentrated under reduced pressure to give pure allylic methyl carbonate **2c** as a colorless oil.

***tert*-Butyl (2-(((methoxycarbonyl)oxy)methyl)allyl)carbamate (2c):** colorless oil; TLC (silica gel, *n*-heptane/EtOAc = 4/1): R_f = 0.29 ($KMnO_4$). ^{1}H NMR (300 MHz, *δ*, $CDCl_3$, 298 K): 5.20–5.17 (m, 1H), 5.16 (s, 1H), 4.75 (bs, 1H), 4.62 (s, 2H), 3.87–3.66 (m, 5H), 1.43 (s, 9H). ^{13}C NMR (75 MHz, *δ*, $CDCl_3$, 298 K): 155.9, 155.7, 140.7, 115.0, 79.7, 68.7, 55.0, 42.9, 28.5. HRMS (ESI): calcd *m/z* for $C_{11}H_{19}NNaO_5^+$: 268.1155 [*M*+Na]$^+$; found: 268.1153.

2.3.5. Phosphate 2d

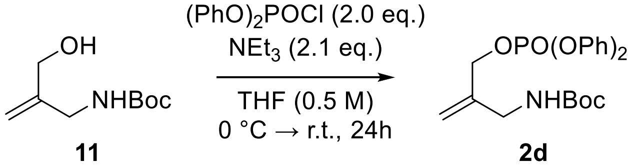

Phosphate **2d** was synthesized in analogy to **2a**. Alcohol **11** (0.15 g, 0.80 mmol), dissolved in dry THF (1.6 mL, 0.5 M) was treated with NEt_3 (0.22 mL, 1.58 mmol, 2.1 equiv) and diphenyl chlorophosphate (0.34 mL, 1.64 mmol, 2.0 equiv). The reaction was quenched with sat. NH_4Cl and filtered through Celite (filter crucible, G4). The white residue was washed with Et_2O (3x) and the phases were separated. The

aqueous phase was extracted with Et_2O (3x) and the combined organic extracts were dried with anhydrous Na_2SO_4, filtered over cotton and concentrated under reduced pressure. Purification by column chromatography (silica gel, *n*-heptane/EtOAc = 1/1) gave allylic phosphate **2d** as a colorless oil in 89% yield (0.30 g, 0.72 mmol).

***tert*-Butyl (2-(((diphenoxyphosphoryl)oxy)methyl)allyl)carbamate (2d):** colorless oil; TLC (silica gel, *n*-heptane/EtOAc = 1/1): R_f = 0.42 (UV). ^{1}H NMR (300 MHz, *δ*, $CDCl_3$, 298 K): 7.38–7.33 (m, 4H), 7.27–7.18 (m, 6H), 5.25–5.20 (m, 1H), 5.18 (s, 1H), 4.78 (bs, 1H), 4.73 (d, *J* = 8.2 Hz, 2H), 3.75 (d, *J* = 5.3 Hz, 2H), 1.44 (s, 9H). ^{13}C NMR (75 MHz, *δ*, $CDCl_3$, 298 K): 155.9, 150.6 (d, $J_{C\text{-}P}$ = 7.2 Hz), 140.7 (d, $J_{C\text{-}P}$ = 5.1 Hz), 130.0, 125.6 (d, $J_{C\text{-}P}$ = 1.3 Hz), 120.2 (d, $J_{C\text{-}P}$ = 4.9 Hz), 115.7, 79.8, 69.8 (d, $J_{C\text{-}P}$ = 5.9 Hz), 42.6, 28.5. ^{31}P NMR (121 MHz, *δ*, $CDCl_3$, 298 K): -11.8. HRMS (ESI): calcd *m/z* for $C_{21}H_{26}NNaO_6P^+$: 442.1390 [*M*+Na]$^+$; found: 442.1393.

2.3.6. Pivalate 2e

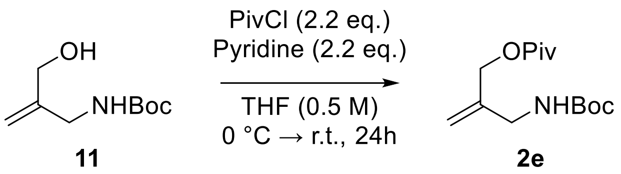

Pivalate **2e** was synthesized in analogy to **2a**. Alcohol **11** (0.14 g, 0.75 mmol), dissolved in dry THF (1.6 mL, 0.5 M) was treated with pyridine (0.13 mL, 1.61 mmol, 2.2 equiv) and pivaloyl chloride (0.20 mL, 1.62 mmol, 2.2 equiv). Aqueous work-up (sat. NH_4Cl, Et_2O, brine and deionized water) followed by column chromatography (silica gel, *n*-heptane/EtOAc = 5/1→4/1) gave allylic pivalate **2e** as a colorless oil in 69% yield (0.14 g, 0.52 mmol).

2-(((*tert*-Butoxycarbonyl)amino)methyl)allyl pivalate (2e): colorless oil; TLC (silica gel, *n*-heptane/EtOAc = 4/1): R_f = 0.32 ($KMnO_4$). ^{1}H NMR (300 MHz, *δ*, $CDCl_3$, 298 K): 5.16–5.09 (m, 2H), 4.74 (bs, 1H), 4.55 (s, 2H), 3.76 (d, *J* = 5.3 Hz, 2H), 1.43 (s, 9H), 1.21 (s, 9H). ^{13}C NMR (75 MHz, *δ*, $CDCl_3$, 298 K): 178.3, 155.9, 141.4, 113.9, 79.7, 65.2, 43.1, 39.0, 28.5, 27.3. HRMS (ESI): calcd *m/z* for $C_{14}H_{25}NNaO_4^+$: 294.1676 [*M*+Na]$^+$; found: 294.1679.

3. Reaction Optimization – Further Conditions Tested

3.1. Lewis Base Screening

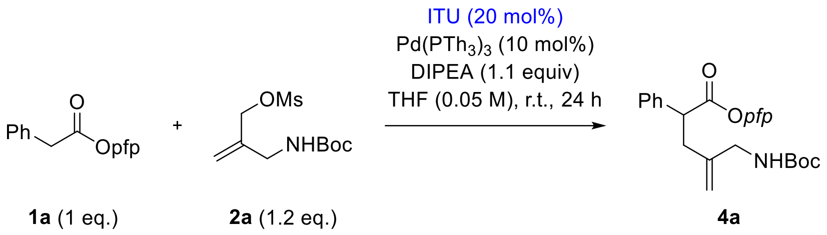

Table 1 – Lewis base screening results. Reactions were performed using 0.1 mmol **1a**, 1.2 equiv **2a**, 20 mol% ITU catalyst, 10 mol% $Pd(PTh_3)_3$, 1.1 equiv DIPEA and 0.05 M THF for 24 h at room temperature. For a detailed procedure see: **4.1**. [a] NMR yields using *o*-xylene as internal standard. [b] Determined by HPLC using a chiral stationary phase.

ITU	Yield[a]	*e.r.*[b]
(*R*)-BTM	65%	97/03
(+)-HyperBTM	16%	60/40
(*S*)-HBTM	30%	77/23
(-)-Tetramisole	20%	69/31

3.2. Alternative Solvent Screening

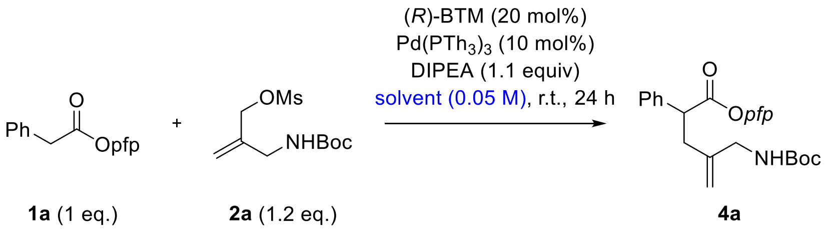

Table 2 – Solvent screening results. Reactions were performed using 0.1 mmol **1a**, 1.2 equiv **2a**, 20 mol% (*R*)-BTM, 10 mol% $Pd(PTh_3)_3$, 1.1 equiv DIPEA and 0.05 M solvent for 24 h at room temperature. For a detailed procedure see: **4.1**. [a] NMR yields using *o*-xylene as internal standard. [b] Determined by HPLC using a chiral stationary phase.

Solvent	Yield[a]	*e.r.*[b]
1,4-Dioxane	46%	96/04
CH_2Cl_2	45%	84/16
Toluene	35%	96/04
CPME	22%	92/08
Bu_2O	-	-
2-MeTHF	43%	97/03

3.3. Alternative Electrophile Screening

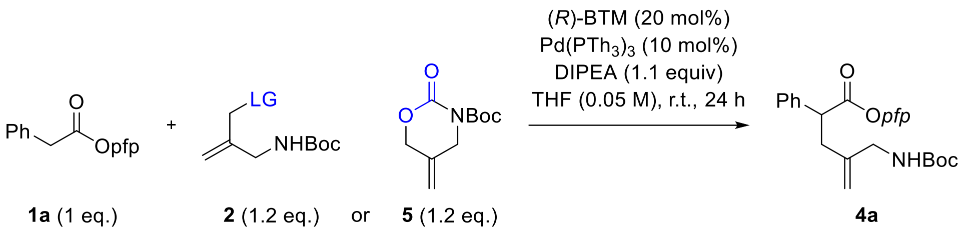

Table 3 – Electrophile screening results. Reactions were performed using 0.1 mmol **1a**, 1.2 equiv **2** or **5**[4], 20 mol% (*R*)-BTM, 10 mol% $Pd(PTh_3)_3$, 1.1 equiv DIPEA and 0.05 M THF for 24 h at room temperature. For a detailed procedure see: **4.1**. [a] NMR yields using *o*-xylene as internal standard. [b] Determined by HPLC using a chiral stationary phase.

Electrophile	Yield[a]	*e.r.*[b]
LG = OAc (**2b**)	-	-
LG = OCO_2Me (**2c**)	49%	51/49
LG = OCO_2tBu[9]	49%	53/47
LG = $OPO(OPh)_2$ (**2d**)	38%	90/10
LG = OPiv (**2e**)	-	-
**5**	-	-

3.4. Alternative Base Screening

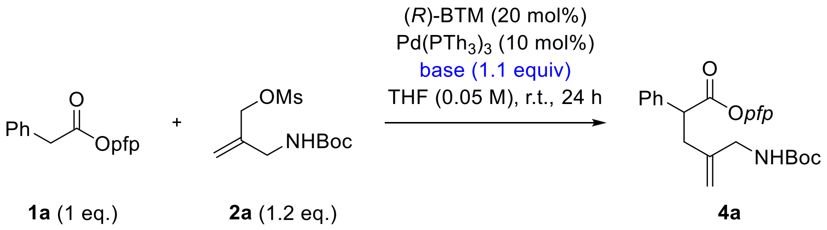

Table 4 – Base screening results. Reactions were performed using 0.1 mmol **1a**, 1.2 equiv **2a**, 20 mol% (*R*)-BTM, 10 mol% $Pd(PTh_3)_3$, 1.1 equiv base and 0.05 M THF for 24 h at room temperature. For a detailed procedure see: **4.1**. [a] NMR yields using *o*-xylene as internal standard. [b] Determined by HPLC using a chiral stationary phase.

Base	Yield[a]	*e.r.*[b]
NEt_3	67%	89/11
DABCO	11%	95/05
Imidazole	4%	-
DBU	13%	78/22
TMP	73%	91/09

[4] V. García-Vázquez, L. Hoteite, C. P. Lakeland, D. W. Watson and J. P. A. Harrity, *Org. Lett.*, **2021,** *23*, 2811–2815.

[9] S.-P. Yuan, Q. Bao, T.-J. Sun, J.-Q. Zhao, Z.-H. Wang, Y. You, Y.-P. Zhang, M.-Q. Zhou and W.-C. Yuan, *Org. Lett.*, **2022,** *24*, 8348–8353.

3.5. Alternative Catalyst Loading Screening

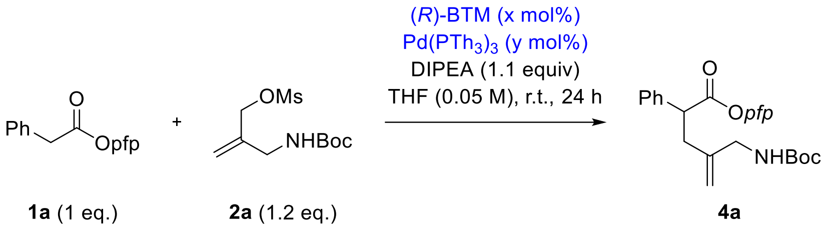

Table 5 – Catalyst loading screening results. Reactions were performed using 0.1 mmol **1a**, 1.2 equiv **2a**, x mol% (*R*)-BTM, y mol% $Pd(PTh_3)_3$, 1.1 equiv DIPEA and 0.05 M THF for 24h at room temperature. For a detailed procedure see: **4.1**. [a] NMR yields using *o*-xylene as internal standard. [b] Determined by HPLC using a chiral stationary phase.

ITU / mol%	[Pd] / mol%	Yield[a]	*e.r.*[b]
20	5	27%	96/04
5	10	32%	84/16
10	10	47%	93/07
35	10	55%	97/03

3.6. Alternative Temperature Screening

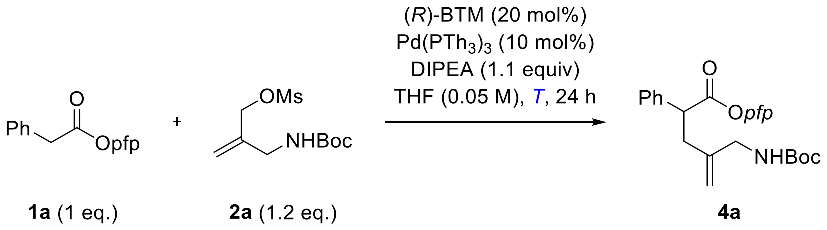

Table 6 – Temperature screening results. Reactions were performed using 0.1 mmol **1a**, 1.2 equiv **2a**, 20 mol% (*R*)-BTM, 10 mol% $Pd(PTh_3)_3$, 1.1 equiv DIPEA and 0.05 M THF for 24 h at the specified temperature. For a detailed procedure see: **4.1**. [a] NMR yields using *o*-xylene as internal standard. [b] Determined by HPLC using a chiral stationary phase.

T / °C	Yield[a]	*e.r.*[b]
+40	48%	95/05
0	46%	97/03
-20	24%	96/04

3.7. Time Study

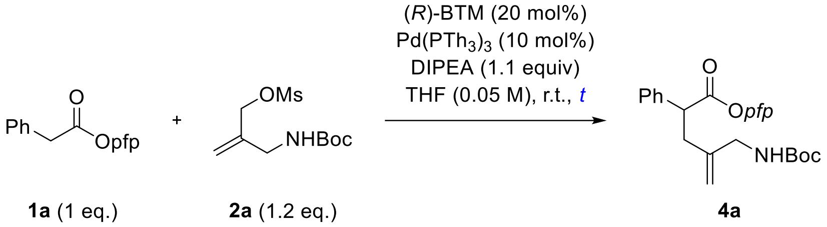

Table 7 – Time study results. Reactions were performed using 0.1 mmol **1a**, 1.2 equiv **2a**, 20 mol% (*R*)-BTM, 10 mol% $Pd(PTh_3)_3$, 1.1 equiv DIPEA and 0.05 M THF for the specified time at room temperature. For a detailed procedure see: **4.1**. [a] NMR yields using *o*-xylene as internal standard. [b] Determined by HPLC using a chiral stationary phase.

t / h	Yield[a]	*e.r.*[b]
1	38%	97/03
2	46%	97/03
6	60%	97/03
12	60%	97/03
24	62%	97/03
48	69%	96/04

4. Syntheses and Analytical Details of Targets 3, 4, 6, 7 and 8

4.1. Allylation – Deprotection/Cyclization Protocol

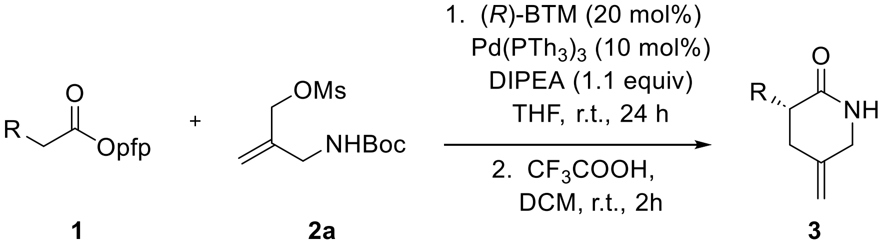

General Procedure: A flame-dried Schlenk tube equipped with a magnetic stirring bar was charged with $Pd(PTh_3)_3$ (9.5 mg, 10 mol%). The flask was evacuated and backfilled with argon (3x), dry and degassed THF (2 mL, 0.05 M with respect to **2a**) was added and the mixture was stirred for 5 minutes to give a clear yellow solution. Pfp ester **1** (125 µmol, 1.25 equiv), DIPEA (18.7 µL, 110 µmol, 1.1 equiv), (*R*)-BTM (5.0 mg, 20 mol%) and mesylate **2a** (26.5 mg, 100 µmol, 1 equiv) were added sequentially and the reaction was stirred for 24 h under argon atmosphere. The mixture was concentrated under reduced pressure and filtered through a short pad of silica gel (DCM). Evaporation of the solvent gave crude allylation product **4**, which was used without further purification. The crude product was dissolved in DCM (1 mL) and TFA (400 µL) was added. The reaction was stirred for 2 h before it was concentrated and co-evaporated several times with DCM. Filtration through a short pad of basic Al_2O_3 (DCM/MeOH = 20/1) and concentration under reduced pressure gave crude δ-Lactam **3**, which was purified by column chromatography (silica gel, 2.5 vol% MeOH in DCM).

(*R*)-5-Methylene-3-phenylpiperidin-2-one (3a): obtained in 67% yield as an off-white solid (12.6 mg, 67 µmol); *e.r.* = 97/3. m.p. = 80–83 °C. TLC (silica gel, 2.5 vol% MeOH in DCM): R_f = 0.20 ($KMnO_4$). $[\alpha]_D^{19}$ (*c* 0.96, $CHCl_3$) = -51.7°. ^{1}H NMR (500 MHz, *δ*, $CDCl_3$, 298 K): 7.34–7.31 (m, 2H), 7.26–7.23 (m, 3H), 6.56 (bs, 1H), 5.00 (s, 1H), 4.90 (s, 1H), 4.06–4.00 (m, 2H), 3.71 (dd, *J* = 7.9, 5.9 Hz, 1H), 2.84 (dd, *J* = 13.9, 5.9 Hz, 1H), 2.69 (dd, *J* = 13.9, 7.9 Hz, 1H). ^{13}C NMR (75 MHz, *δ*, $CDCl_3$, 298 K): 173.3, 139.8, 137.7, 128.7, 128.3, 127.1, 112.3, 48.5, 47.8, 37.6. HPLC (YMC Chiral ART Cellulose-SB, eluent: *n*-hexane:*i*-PrOH = 4/1, 1.0 mL·min^{-1}, 10 °C, λ = 210 nm) retention times: t_{major} = 13.8 min, t_{minor} = 23.9 min. HRMS (ESI): calcd *m/z* for $C_{12}H_{14}NO^+$: 188.1070 $[M+H]^+$; found: 188.1071.

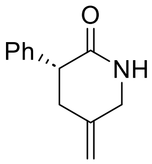

3a

(*R*)-3-(4-Fluorophenyl)-5-methylenepiperidin-2-one (3b): obtained in 61% yield as an off-white solid (12.5 mg, 61 µmol); *e.r.* = 97/3. m.p. = 110–113 °C. TLC (silica gel, 2.5 vol% MeOH in DCM): R_f = 0.19 ($KMnO_4$). $[\alpha]_D^{19}$ (*c* 0.99, $CHCl_3$) = -47.3°. ^1H NMR (300 MHz, *δ*, $CDCl_3$, 298 K): 7.23–7.17 (m, 2H), 7.04–6.97 (m, 2H), 6.69 (bs, 1H), 5.01 (s, 1H), 4.90 (s, 1H), 4.04–4.00 (m, 2H), 3.68 (dd, *J* = 8.1, 5.9 Hz, 1H), 2.82 (dd, *J* = 13.9, 5.9 Hz, 1H), 2.64 (dd, *J* = 13.9, 8.1 Hz, 1H). ^{13}C NMR (75 MHz, *δ*, $CDCl_3$, 298 K): 173.1, 162.0 (d, $J_{C\text{-}F}$ = 245.1 Hz), 137.5, 135.5 (d, $J_{C\text{-}F}$ = 3.3 Hz), 129.8 (d, $J_{C\text{-}F}$ = 8.0 Hz), 115.5 (d, $J_{C\text{-}F}$ = 21.4 Hz), 112.5, 47.8, 47.7, 37.6. ^{19}F NMR (282 MHz, δ, $CDCl_3$, 298 K): -115.9 (tt, *J* = 8.4, 5.6 Hz). HPLC (YMC Chiral ART Cellulose-SB, eluent: *n*-hexane:*i*-PrOH = 4/1, 1.0 mL·min^{-1}, 10 °C, *λ* = 210 nm) retention times: t_{major} = 14.4 min, t_{minor} = 25.6 min. HRMS (ESI): calcd *m/z* for $C_{12}H_{13}FNO^+$: 206.0976 $[M+H]^+$; found: 206.0978.

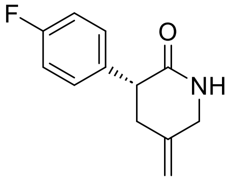

3b

(*R*)-3-(4-Chlorophenyl)-5-methylenepiperidin-2-one (3c): obtained in 65% yield as a white solid (14.4 mg, 65 µmol); *e.r.* = 94/6. m.p. = 109–111 °C. TLC (silica gel, 2.5 vol% MeOH in DCM): R_f = 0.19 ($KMnO_4$). $[\alpha]_D^{20}$ (*c* 0.99, $CHCl_3$) = -56.8°. ^1H NMR (300 MHz, *δ*, $CDCl_3$, 298 K): 7.31–7.27 (m, 2H), 7.19–7.15 (m, 2H), 6.80 (bs, 1H), 5.01 (s, 1H), 4.90 (s, 1H), 4.03–3.98 (m, 2H), 3.67 (dd, *J* = 8.1, 5.9 Hz, 1H), 2.81 (dd, *J* = 13.9, 5.9 Hz, 1H), 2.63 (dd, *J* = 13.9, 8.1 Hz, 1H). ^{13}C NMR (75 MHz, *δ*, $CDCl_3$, 298 K): 172.9, 138.3, 137.4, 133.0, 129.7, 128.8, 112.6, 47.9, 47.7, 37.5. HPLC (YMC Chiral ART Cellulose-SB, eluent: *n*-hexane:*i*-PrOH = 4/1, 1.0 mL·min^{-1}, 10 °C, *λ* = 210 nm) retention times: t_{major} = 15.9 min, t_{minor} = 27.1 min. HRMS (ESI): calcd *m/z* for $C_{12}H_{13}ClNO^+$: 222.0680 $[M+H]^+$; found: 222.0683.

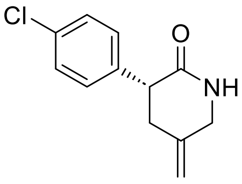

3c

(*R*)-3-(4-Bromophenyl)-5-methylenepiperidin-2-one (3d): obtained in 66% yield as a white solid (17.5 mg, 66 µmol); *e.r.* = 94/6. m.p. = 137–138 °C. TLC (silica gel, 2.5 vol% MeOH in DCM): R_f = 0.20 ($KMnO_4$). $[\alpha]_D^{20}$ (*c* 1.01, $CHCl_3$) = -50.9°. ^1H NMR (300 MHz, *δ*, $CDCl_3$, 298 K): 7.47–7.42 (m, 2H), 7.14–7.09 (m, 2H), 6.74 (bs, 1H), 5.01 (s, 1H), 4.90 (s, 1H), 4.03–3.99 (m, 2H), 3.65 (dd, *J* = 8.1, 5.9 Hz, 1H), 2.81 (dd, *J* = 13.9, 5.9 Hz, 1H), 2.63 (dd, *J* = 13.9, 8.1 Hz, 1H). ^{13}C NMR (75 MHz, *δ*, $CDCl_3$, 298 K): 172.8, 138.8, 137.3, 131.8, 130.1, 121.1, 112.6, 48.0, 47.8, 37.4. HPLC (YMC Chiral ART Cellulose-SB, eluent: *n*-hexane:*i*-PrOH = 4/1, 1.0 mL·min^{-1}, 10 °C, *λ* = 210 nm) retention times: t_{major} = 17.0 min, t_{minor} = 28.8 min. HRMS (ESI): calcd *m/z* for $C_{12}H_{13}BrNO^+$: 266.0175 $[M+H]^+$; found: 266.0179.

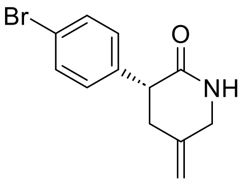

3d

(*R*)-3-(4-Methoxyphenyl)-5-methylenepiperidin-2-one (3e): obtained in 62% yield as a white solid (13.5 mg, 62 µmol); *e.r.* = 98/2. m.p. = 136–139 °C. TLC (silica gel, 2.5 vol% MeOH in DCM): R_f = 0.20 ($KMnO_4$). $[\alpha]_D^{20}$ (*c* 0.97, $CHCl_3$) = -63.2°. ^1H NMR (300 MHz, *δ*, $CDCl_3$, 298 K): 7.18–7.13 (m, 2H), 6.88–6.83 (m, 3H), 4.98 (s, 1H), 4.89 (s, 1H), 4.02–3.98 (m, 2H), 3.78 (s, 3H), 3.64 (dd, *J* = 8.0, 5.9 Hz, 1H), 2.80 (dd, *J* = 13.9, 5.9 Hz, 1H), 2.64 (dd, *J* = 13.9, 8.0 Hz, 1H). ^{13}C NMR (75 MHz, *δ*, $CDCl_3$, 298 K): 173.7, 158.6, 138.0, 131.9, 129.3, 114.1, 112.1, 55.4, 47.7, 47.6, 37.6. HPLC (CHIRALPAK OD-H, eluent: *n*-hexane:*i*-PrOH = 4/1, 1.0 mL·min^{-1}, 10 °C, λ = 210 nm) retention times: t_{major} = 17.8 min, t_{minor} = 31.7 min. HRMS (ESI): calcd *m/z* for $C_{13}H_{16}NO_2^+$: 218.1176 $[M+H]^+$; found: 218.1175.

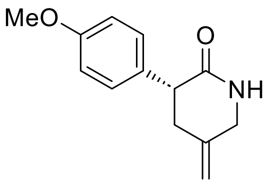

3e

(*R*)-5-Methylene-3-(4-(methylthio)phenyl)piperidin-2-one (3f): obtained in 71% yield as a white solid (16.6 mg, 71 µmol); *e.r.* = 96/4. m.p. = 152–154 °C. TLC (silica gel, 2.5 vol% MeOH in DCM): 0.20 ($KMnO_4$). $[\alpha]_D^{20}$ (*c* 0.98, $CHCl_3$) = -76.9°. ^1H NMR (300 MHz, *δ*, $CDCl_3$, 298 K): 7.24–7.21 (m, 2H), 7.18–7.15 (m, 2H), 6.85 (bs, 1H), 5.00 (s, 1H), 4.90 (s, 1H), 4.04–3.98 (m, 2H), 3.66 (dd, *J* = 8.0, 5.9 Hz, 1H), 2.81 (dd, *J* = 13.9, 5.9 Hz, 1H), 2.65 (dd, *J* = 13.9, 8.0 Hz, 1H), 2.47 (s, 3H). ^{13}C NMR (75 MHz, *δ*, $CDCl_3$, 298 K): 173.1, 137.7, 137.1, 136.7, 128.8, 127.1, 112.4, 48.0, 47.8, 37.5, 16.1. HPLC (CHIRALPAK OD-H, eluent: *n*-hexane:*i*-PrOH = 4/1, 1.0 mL·min^{-1}, 10 °C, λ = 210 nm) retention times: t_{major} = 19.3 min, t_{minor} = 37.4 min. HRMS (ESI): calcd *m/z* for $C_{13}H_{16}NOS^+$: 234.0947 $[M+H]^+$; found: 234.0948.

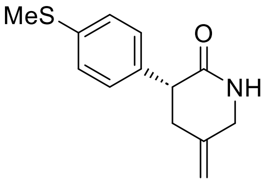

3f

(*R*)-3-(4-(*tert*-Butyl)phenyl)-5-methylenepiperidin-2-one (3g): obtained in 41% yield as a white solid (10.0 mg, 41 µmol); *e.r.* = 97/3. m.p. = 138–141 °C. TLC (silica gel, 2.5 vol% MeOH in DCM): 0.20 ($KMnO_4$). $[\alpha]_D^{20}$ (*c* 0.84, $CHCl_3$) = -53.8°. ^1H NMR (300 MHz, *δ*, $CDCl_3$, 298 K): 7.36–7.31 (m, 2H), 7.19–7.15 (m, 2H), 6.43 (bs, 1H), 5.00 (s, 1H), 4.92 (s, 1H), 4.08–3.97 (m, 2H), 3.66 (dd, *J* = 8.2, 6.0 Hz, 1H), 2.82 (dd, *J* = 13.9, 6.0 Hz, 1H), 2.69 (dd, *J* = 13.9, 8.2 Hz, 1H), 1.30 (s, 9H). ^{13}C NMR (75 MHz, *δ*, $CDCl_3$, 298 K): 173.4, 149.7, 138.0, 136.5, 127.8, 125.5, 111.9, 47.9, 47.7, 37.4, 34.4, 31.3. HPLC (YMC Chiral ART Cellulose-SB, eluent: *n*-hexane:*i*-PrOH = 4/1, 1.0 mL·min^{-1}, 10 °C, λ = 210 nm) retention times: t_{major} = 11.7 min, t_{minor} = 17.7 min. HRMS (ESI): calcd *m/z* for $C_{16}H_{22}NO^+$: 244.1696 $[M+H]^+$; found: 244.1697.

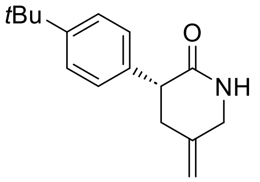

3g

(*R*)-5-Methylene-3-(*p*-tolyl)piperidin-2-one (3h): obtained in 59% yield as a white solid (11.9 mg, 59 µmol); *e.r.* = 98/2. m.p. = 97–100 °C. TLC (silica gel, 2.5 vol% MeOH in DCM): 0.19 ($KMnO_4$). $[\alpha]_D^{20}$ (*c* 1.05, $CHCl_3$) = -61.2°. ^{1}H NMR (300 MHz, *δ*, $CDCl_3$, 298 K): 7.13 (s, 4H), 6.55 (bs, 1H), 4.99 (s, 1H), 4.90 (s, 1H), 4.06–3.96 (m, 2H), 3.66 (dd, *J* = 8.0, 5.9 Hz, 1H), 2.81 (dd, *J* = 13.9, 5.9 Hz, 1H), 2.67 (dd, *J* = 13.9, 8.0 Hz, 1H), 2.32 (s, 3H). ^{13}C NMR (75 MHz, *δ*, $CDCl_3$, 298 K): 173.6, 138.0, 136.8, 136.7, 129.4, 128.1, 112.1, 48.1, 47.8, 37.6, 21.2. HPLC (YMC Chiral ART Cellulose-SB, eluent: *n*-hexane:*i*-PrOH = 4/1, 1.0 mL·min^{-1}, 10 °C, λ = 210 nm) retention times: t_{major} = 12.7 min, t_{minor} = 21.4 min. HRMS (ESI): calcd *m/z* for $C_{13}H_{16}NO^+$: 202.1226 $[M+H]^+$; found: 202.1229.

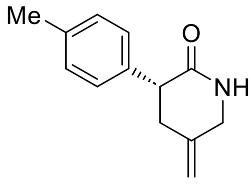

3h

(*R*)-5-Methylene-3-(*m*-tolyl)piperidin-2-one (3i): obtained in 39% yield as a colorless oil (7.9 mg, 39 µmol); *e.r.* = 97/3. TLC (silica gel, 2.5 vol% MeOH in DCM): 0.20 ($KMnO_4$). $[\alpha]_D^{20}$ (*c* 0.79, $CHCl_3$) = -52.5°. ^{1}H NMR (500 MHz, *δ*, $CDCl_3$, 298 K): 7.21 (t, *J* = 7.5 Hz, 1H), 7.07–7.02 (m, 3H), 6.53 (bs, 1H), 4.99 (s, 1H), 4.90 (s, 1H), 4.06–3.99 (m, 2H), 3.65 (dd, *J* = 8.1, 6.0 Hz, 1H), 2.81 (dd, *J* = 13.9, 6.0 Hz, 1H), 2.68 (dd, *J* = 13.9, 8.1 Hz, 1H), 2.33 (s, 3H). ^{13}C NMR (125 MHz, *δ*, $CDCl_3$, 298 K): 173.5, 139.8, 138.2, 137.9, 129.1, 128.5, 127.9, 125.3, 112.1, 48.5, 47.8, 37.7, 21.6. HPLC (YMC Chiral ART Cellulose-SB, eluent: *n*-hexane:*i*-PrOH = 4/1, 1.0 mL·min^{-1}, 10 °C, λ = 210 nm) retention times: t_{major} = 12.3 min, t_{minor} = 17.7 min. HRMS (ESI): calcd *m/z* for $C_{13}H_{16}NO^+$: 202.1226 $[M+H]^+$; found: 202.1223.

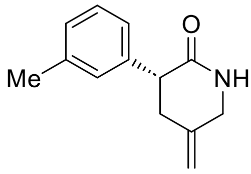

3i

(*R*)-5-Methylene-3-(*o*-tolyl)piperidin-2-one (3j): obtained in 17% yield as a colorless oil (3.4 mg, 17 µmol); *e.r.* = 97/3. TLC (silica gel, 2.5 vol% MeOH in DCM): 0.20 ($KMnO_4$). $[\alpha]_D^{20}$ (*c* 0.34, $CHCl_3$) = -32.2°. ^{1}H NMR (500 MHz, *δ*, $CDCl_3$, 298 K): 7.19–7.09 (m, 4H), 6.18 (bs, 1H), 5.02 (s, 1H), 4.89 (s, 1H), 4.12–4.02 (m, 2H), 3.88 (dd, *J* = 9.0, 6.1 Hz, 1H), 2.76 (dd, *J* = 13.6, 6.1 Hz, 1H), 2.61 (dd, *J* = 13.6, 9.0 Hz, 1H), 2.36 (s, 3H). ^{13}C NMR (125 MHz, *δ*, $CDCl_3$, 298 K): 173.3, 138.4, 137.9, 136.1, 130.8, 128.0, 127.2, 126.3, 112.4, 48.1, 45.6, 36.9, 19.8. HPLC (YMC Chiral ART Cellulose-SB, eluent: *n*-hexane:*i*-PrOH = 4/1, 1.0 mL·min^{-1}, 10 °C, λ = 210 nm) retention times: t_{major} = 11.5 min, t_{minor} = 24.0 min. HRMS (ESI): calcd *m/z* for $C_{13}H_{16}NO^+$: 202.1226 $[M+H]^+$; found: 202.1228.

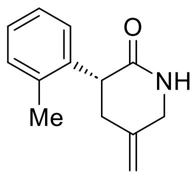

3j

(*R*)-3-(3,4-Dimethoxyphenyl)-5-methylenepiperidin-2-one (3k): obtained in 65% yield as an off-white solid (16.0 mg, 65 µmol); *e.r.* = 96/4. m.p. = 124–126 °C. TLC (silica gel, 2.5 vol% MeOH in DCM): 0.19 ($KMnO_4$). $[\alpha]_D^{20}$ (*c* 0.99, $CHCl_3$) = -64.5°. ^{1}H NMR (300 MHz, δ, $CDCl_3$, 298 K): 6.92 (bs, 1H), 6.85–6.78 (m, 3H), 5.01 (s, 1H), 4.93 (s, 1H), 4.06–3.96 (m, 2H), 3.87 (s, 3H), 3.86 (s, 3H), 3.64 (dd, *J* = 7.9, 6.0 Hz, 1H), 2.83 (dd, *J* = 13.9, 6.0 Hz, 1H), 2.68 (dd, *J* = 13.9, 7.9 Hz, 1H). ^{13}C NMR (75 MHz, δ, $CDCl_3$, 298 K): 173.5, 149.0, 148.2, 138.0, 132.4, 120.3, 112.1, 111.6, 111.3, 56.0 (2), 48.0, 47.7, 37.5. HPLC (CHIRALPAK OD-H, eluent: *n*-hexane:*i*-PrOH = 2/1, 1.0 mL·min^{-1}, 10 °C, λ = 210 nm) retention times: t_{major} = 15.5 min, t_{minor} = 31.7 min. HRMS (ESI): calcd *m/z* for $C_{14}H_{18}NO_3^+$: 248.1281 [*M*+H]$^+$; found: 248.1282.

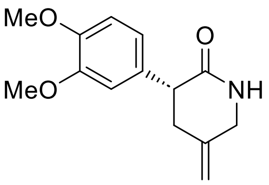

3k

(*R*)-5-Methylene-3-(4-(trifluoromethyl)phenyl)piperidin-2-one (3l): obtained in 59% yield as an off-white solid (15.1 mg, 59 µmol); *e.r.* = 75/25. m.p. = 150–152 °C. TLC (silica gel, 2.5 vol% MeOH in DCM): 0.20 ($KMnO_4$). $[\alpha]_D^{20}$ (*c* 1.04, $CHCl_3$) = -22.3°. ^{1}H NMR (500 MHz, δ, $CDCl_3$, 298 K): 7.58 (d, *J* = 8.2 Hz, 2H), 7.36 (d, *J* = 8.2 Hz, 2H), 6.73 (bs, 1H), 5.03 (s, 1H), 4.92 (s, 1H), 4.07–4.01 (m, 2H), 3.76 (dd, *J* = 8.3, 6.0 Hz, 1H), 2.84 (dd, *J* = 13.9, 6.0 Hz, 1H), 2.67 (dd, *J* = 13.9, 8.3 Hz, 1H). ^{13}C NMR (125 MHz, δ, $CDCl_3$, 298 K): 172.5, 143.8, 137.1, 129.4 (q, J_{C-F} = 32.5 Hz), 128.8, 125.6 (q, J_{C-F} = 3.8 Hz), 124.3 (q, J_{C-F} = 272.1 Hz), 112.8, 48.4, 47.8, 37.4. ^{19}F NMR (470 MHz, δ, $CDCl_3$, 298 K): -62.5 (s). HPLC (YMC Chiral ART Cellulose-SB, eluent: *n*-hexane:*i*-PrOH = 4/1, 1.0 mL·min^{-1}, 10 °C, λ = 210 nm) retention times: t_{major} = 16.0 min, t_{minor} = 25.9 min. HRMS (ESI): calcd *m/z* for $C_{13}H_{13}F_3NO^+$: 256.0944 [*M*+H]$^+$; found: 256.0946.

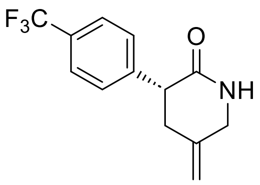

3l

(*R*)-5-Methylene-3-(naphthalen-1-yl)piperidin-2-one (3m): obtained in 39% yield as an off-white solid (9.3 mg, 39 µmol); *e.r.* = 94/6. m.p. = 50–53 °C. TLC (silica gel, 2.5 vol% MeOH in DCM): 0.20 ($KMnO_4$). $[\alpha]_D^{20}$ (*c* 0.93, $CHCl_3$) = +17.9°. ^{1}H NMR (300 MHz, δ, $CDCl_3$, 298 K): 7.96 (d, *J* = 8.3 Hz, 1H), 7.89–7.86 (m, 1H), 7.77 (d, *J* = 8.2 Hz, 1H), 7.56–7.46 (m, 2H), 7.45–7.40 (m, 1H), 7.32 (dd, *J* = 7.1, 1.0 Hz, 1H), 6.84 (bs, 1H), 4.99 (s, 1H), 4.78 (s, 1H), 4.47 (t, *J* = 6.9 Hz, 1H), 4.15–4.04 (m, 2H), 2.92 (dd, *J* = 13.6 Hz, 6.2 Hz, 1H), 2.80 (dd, *J* = 13.6 Hz, 7.5 Hz, 1H). ^{13}C NMR (75 MHz, δ, $CDCl_3$, 298 K): 173.7, 137.4, 136.0, 134.3, 131.3, 129.3, 128.0, 126.3, 126.1, 125.6, 125.4, 123.3, 112.8, 48.0, 45.6, 37.1. HPLC (CHIRALPAK OD-H, eluent: *n*-hexane:*i*-PrOH = 4/1, 1.0 mL·min^{-1}, 10 °C, λ = 210 nm) retention times: t_{major} = 16.7 min, t_{minor} = 36.9 min. HRMS (ESI): calcd *m/z* for $C_{16}H_{16}NO^+$: 238.1226 [*M*+H]$^+$; found: 238.1228.

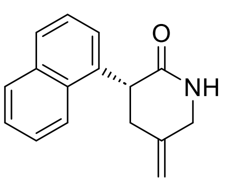

3m

(*R*)-5-Methylene-3-(naphthalen-2-yl)piperidin-2-one (3n): obtained in 73% yield as a brown solid

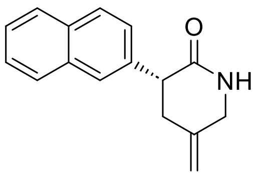

3n

(17.3 mg, 73 µmol); *e.r.* = 96/4. m.p. = 97–100 °C. TLC (silica gel, 2.5 vol% MeOH in DCM): 0.20 ($KMnO_4$). $[\alpha]_D^{20}$ (*c* 1.02, $CHCl_3$) = -81.0°. ^1H NMR (300 MHz, δ, $CDCl_3$, 298 K): 7.83–7.78 (m, 3H), 7.69 (s, 1H), 7.49–7.41 (m, 2H), 7.38 (dd, J = 8.5, 1.8 Hz, 1H), 6.91 (bs, 1H), 4.99 (s, 1H), 4.89 (s, 1H), 4.09–3.98 (m, 2H), 3.88 (dd, J = 8.0, 6.0 Hz, 1H), 2.89 (dd, J = 13.9, 6.0 Hz, 1H), 2.78 (dd, J = 13.9, 8.0 Hz, 1H). ^{13}C NMR (75 MHz, δ, $CDCl_3$, 298 K): 173.2, 137.7, 137.2, 133.6, 132.7, 128.4, 127.9, 127.7, 127.1, 126.4, 126.2, 125.9, 112.4, 48.6, 47.9, 37.6. HPLC (CHIRALPAK OD-H, eluent: *n*-hexane:*i*-PrOH = 4/1, 1.0 mL·min^{-1}, 10 °C, λ = 210 nm) retention times: t_{major} = 17.9 min, t_{minor} = 31.6 min. HRMS (ESI): calcd *m/z* for $C_{16}H_{16}NO^+$: 238.1226 [*M*+H]$^+$; found: 238.1226.

(*R*)-5-Methylene-3-(thiophen-3-yl)piperidin-2-one (3o): obtained in 66% yield as an off-white solid

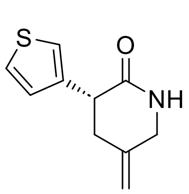

3o

(12.8 mg, 66 µmol); *e.r.* = 95/5. m.p. = 102–104 °C. TLC (silica gel, 2.5 vol% MeOH in DCM): 0.19 ($KMnO_4$). $[\alpha]_D^{20}$ (*c* 0.99, $CHCl_3$) = -62.4°. ^1H NMR (300 MHz, δ, $CDCl_3$, 298 K): 7.28 (dd, J = 5.0, 3.0 Hz, 1H), 7.15–7.13 (m, 1H), 7.06 (dd, J = 5.0, 1.3 Hz, 1H), 6.55 (bs, 1H), 5.01 (s, 1H), 4.96 (s, 1H), 4.05–3.95 (m, 2H), 3.81 (t, J = 6.3 Hz, 1H), 2.86 (dd, J = 14.1, 5.6 Hz, 1H), 2.74 (dd, J = 14.1, 6.9 Hz, 1H). ^{13}C NMR (75 MHz, δ, $CDCl_3$, 298 K): 172.9, 139.5, 137.8, 127.6, 125.7, 121.9, 112.3, 47.5, 43.7, 36.4. HPLC (CHIRALPAK OD-H, eluent: *n*-hexane:*i*-PrOH = 4/1, 1.0 mL·min^{-1}, 10 °C, λ = 210 nm) retention times: t_{major} = 16.2 min, t_{minor} = 25.6 min. HRMS (ESI): calcd *m/z* for $C_{10}H_{12}NOS^+$: 194.0634 [*M*+H]$^+$; found: 194.0635.

(*R*)-3-(2,2-Diphenylvinyl)-5-methylenepiperidin-2-one (3p): obtained in 53% yield as an off-white

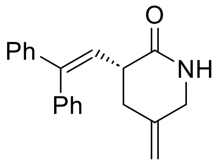

3p

solid (15.2 mg, 53 µmol); *e.r.* = 90/10. m.p. = 152–154 °C. TLC (silica gel, 2.5 vol% MeOH in DCM): 0.24 ($KMnO_4$). $[\alpha]_D^{20}$ (*c* 1.01, $CHCl_3$) = -102.6°. ^1H NMR (300 MHz, δ, $CDCl_3$, 298 K): 7.41–7.30 (m, 5H), 7.25–7.22 (m, 5H), 6.20 (bs, 1H), 6.09 (d, J = 10.1 Hz, 1H), 4.97 (s, 1H), 4.92 (s, 1H), 4.01–3.89 (m, 2H), 3.27 (ddd, J = 10.1, 8.9, 5.7 Hz, 1H), 2.55 (dd, J = 13.8, 5.7 Hz, 1H), 2.45 (dd, J = 13.8, 8.9 Hz, 1H). ^{13}C NMR (75 MHz, δ, $CDCl_3$, 298 K): 173.6, 144.7, 142.3, 139.5, 137.9, 130.1, 128.4, 128.2, 127.7, 127.5 (2), 126.0, 111.8, 47.6, 42.8, 36.3. HPLC (YMC Chiral ART Cellulose-SB, eluent: *n*-hexane:*i*-PrOH = 4/1, 1.0 mL·min^{-1}, 10 °C, λ = 210 nm) retention times: t_{major} = 7.3 min, t_{minor} = 12.1 min. HRMS (ESI): calcd *m/z* for $C_{20}H_{20}NO^+$: 290.1539 [*M*+H]$^+$; found: 290.1535.

4.2. Acyclic Allylated Ester Intermediate 4a

The crude allylation product was purified by column chromatography (silica gel, n-heptane/EtOAc = 7/1→5/1) to obtain **4** in the given yield and enantioselectivity.

Perfluorophenyl (*R*)-4-(((*tert*-butoxycarbonyl)amino)methyl)-2-phenylpent-4-enoate (4a): obtained in 68% yield as a colorless oil [322 mg, 0.68 mmol (from 1 mmol **2a)**]; *e.r.* = 97/3. TLC (silica gel, *n*-heptane/EtOAc = 7/1): R_f = 0.25 ($KMnO_4$/UV). $[\alpha]_D^{21}$ (*c* 1.00, $CHCl_3$) = -56.4°. ^{1}H NMR (300 MHz, *δ*, $CDCl_3$, 298 K): 7.40–7.29 (m, 5H), 5.02 (s, 1H), 4.93 (s, 1H), 4.62 (bs, 1H), 4.31–4.26 (m, 1H), 3.82–3.69 (m, 2H), 2.94 (dd, *J* = 15.1, 9.3 Hz, 1H), 2.57 (dd, *J* = 15.1, 6.2 Hz, 1H). 1.45 (s, 9H). ^{13}C NMR (75 MHz, *δ*, $CDCl_3$, 298 K): 169.7, 156.1, 143.1, 137.0, 129.1, 128.2, 128.0, 113.5, 79.8, 49.4, 45.3, 37.3, 28.5. ^{19}F NMR (282 MHz, δ, $CDCl_3$, 298 K): -152.3 (d, *J* = 17.8 Hz, 2F), -158.0 (t, *J* = 21.7 Hz, 1F), -162.4 (dd, *J* = 21.7, 17.8 Hz, 2F). HPLC (YMC Chiral ART Amylose-SA, eluent: *n*-hexane:*i*-PrOH = 50/1, 0.7 mL·min^{-1}, 10 °C, λ = 210 nm) retention times: t_{major} = 18.8 min, t_{minor} = 16.3 min. HRMS (ESI): calcd *m/z* for $C_{23}H_{22}F_5NNaO_4^+$: 494.1361 [*M*+Na]$^+$; found: 494.1361.

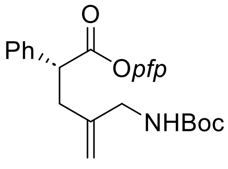

4a

4.3. Further Transformations and Products

4.3.1. Oxidative Cleavage of 3a

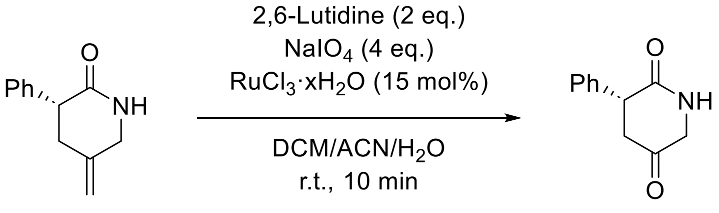

3a **6a**

Following a known procedure[10], δ-Lactam **3a** (9.9 mg, 53 µmol, 1 equiv, *e.r.* = 97/3) was transferred to a Schlenk tube equipped with a stirring bar. DCM (0.3 mL), ACN (0.3 mL), 2,6-lutidine (12.5 µL, 108 µmol, 2 equiv), H_2O (0.3 mL) and $NaIO_4$ (45.7 mg, 214 µmol, 4 equiv) were added sequentially, before adding a solution of $RuCl_3$·xH_2O (1.6 mg, 15 mol%) in H_2O (0.2 mL) dropwise under vigorous stirring. The reaction was stirred for 10 min, after which time the suspension's color changed rapidly from light brown to black. The phases were separated, and the aqueous phase was extracted with EtOAc (3x). The combined organic extracts were washed with brine, dried with anhydrous Na_2SO_4, filtered over cotton, and concentrated under reduced pressure. Purification by preparative TLC (silica gel,

[10] D. W. Watson, M. Gill, P. Kemmitt, S. G. Lamont, M. V. Popescu and I. Simpson, *Tetrahedron Lett.*, **2018,** *59,* 4479–4482.

DCM/MeOH = 20/1) gave **6a** as an off-white solid in 65% yield (6.5 mg, 34 µmol). Full retention of the compound's stereochemistry was confirmed *via* HPLC using a chiral stationary phase.

(*R*)-3-Phenylpiperidine-2,5-dione (6a): off-white solid; *e.r.* = 97/3. m.p. = 142–144 °C. TLC (silica gel, DCM/MeOH = 20/1): 0.17 (UV). $[\alpha]_D^{20}$ (*c* 0.66, $CHCl_3$) = +3.8°. ^1H NMR (300 MHz, *δ*, $CDCl_3$, 298 K): 7.40–7.26 (m, 5H), 6.59 (bs, 1H), 4.03 (t, *J* = 6.3 Hz, 1H), 3.94 (dd, *J* = 19.4, 2.6 Hz, 1H), 3.81 (dd, *J* = 19.4, 2.0 Hz, 1H), 3.11 (dd, *J* = 17.0, 6.8 Hz, 1H), 3.02 (dd, *J* = 17.0, 5.7 Hz, 1H). ^{13}C NMR (75 MHz, *δ*, $CDCl_3$, 298 K): 204.1, 172.8, 135.8, 129.2, 128.0, 127.5, 51.5, 45.0, 42.2. HPLC (YMC Chiral ART Cellulose-SB, eluent: *n*-hexane:*i*-PrOH = 1/1, 1.0 mL·min^{-1}, 10 °C, λ = 210 nm) retention times: t_{major} = 21.4 min, t_{minor} = 20.3 min. HRMS (ESI): calcd *m/z* for $C_{11}H_{12}NO_2^+$: 190.0863 $[M+H]^+$; found: 190.0865.

4.3.2. Double Bond Hydrogenation of 3a

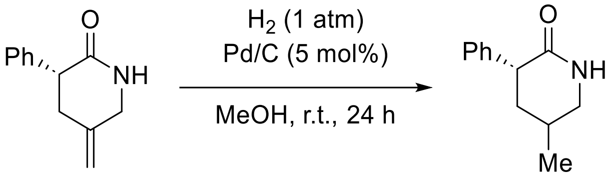

3a **7a**

A flame-dried Schlenk tube equipped with a stirring bar was charged with 10% Pd/C (2.8 mg, 5 mol%). The flask was evacuated and backfilled with argon (3x), before adding a solution of δ-Lactam **3a** (9.9 mg, 53 µmol, *e.r.* = 97/3) in MeOH (1 mL, 0.05 M with respect to **3a**) *via* syringe. The mixture was degassed by means of three freeze-pump-thaw cycles, filled with H_2-gas (balloon), and vigorously stirred for 24 h at room temperature. Filtration over Celite (DCM) and evaporation of the solvent gave pure **7a** as a white solid in 99% yield (9.9 mg, 52 µmol). The product was obtained as an inseparable mixture of diastereomers. Full retention of the compound's stereochemistry was confirmed *via* HPLC using a chiral stationary phase.

(3*R*)-5-Methyl-3-phenylpiperidin-2-one (7a): white solid; *d.r.* = 65/35. *e.r.* = 97/3 (both diast). m.p. = 146–149 °C. TLC (silica gel, DCM/MeOH = 20/1): 0.30 (UV). $[\alpha]_D^{20}$ (*c* 1.00, $CHCl_3$) = +4.1°. ^1H NMR (300 MHz, *δ*, DMSO-d_6, 298 K): 7.69 and 7.57 (bs, 1H), 7.32–7.14 (m, 5H), 3.59–3.56 and 3.49 (*minor*: m; *major*: dd, *J* = 12.4, 6.1 Hz; 1H), 3.31–3.15 (m, 1H), 2.95–2.81 (m, 1H), 2.08–1.52 (m, 3H), 0.94 and 0.90 (*major*: d, *J* = 6.5 Hz; *minor*: d, *J* = 6.2 Hz; 3H). ^{13}C NMR (75 MHz, *δ*, $CDCl_3$, 298 K): 173.3, 173.0, 141.8, 141.3, 128.7 (2), 128.5, 128.2, 126.9, 126.7, 49.9, 49.5, 49.0, 46.5, 40.1, 37.8, 29.3, 23.8, 18.7, 18.3. HPLC (YMC Chiral ART Cellulose-SB, eluent: *n*-hexane:*i*-PrOH = 4/1, 0.5 mL·min^{-1}, 10 °C, λ = 210 nm) retention times: t_{R1} = 21.8 min (*minor* diast.), t_{R2} = 36.3 min (*minor* diast.), t_{R3} = 37.2 min (*major* diast.), t_{R4} = 48.7 min (*major* diast.). HRMS (ESI): calcd *m/z* for $C_{12}H_{16}NO^+$: 190.1226 $[M+H]^+$; found: 190.1231.

4.3.3. Epoxidation of 3a

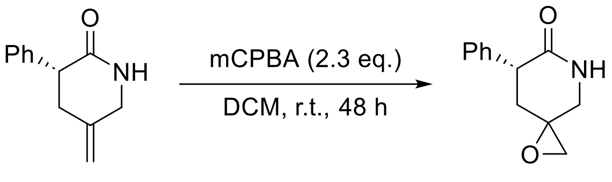

δ-Lactam **3a** (20.4 mg, 109 µmol, 1 equiv) was dissolved in dry DCM (0.7 mL, 0.16 M with respect to **3a**). The solution was cooled in an ice-bath before adding mCPBA (56.4 mg, 252 µmol, 2.3 equiv, ≤77%) portionwise. The ice-bath was removed, and the reaction was stirred for 48 hours at room temperature. The reaction was quenched with Na_2SO_3 (135 mg, 1.07 mmol, 9.8 equiv) and sat. $NaHCO_3$ (1 mL). The biphasic mixture was stirred for 30 min and the phases were separated. The organic phase was washed with sat. $NaHCO_3$ (3x) and brine (2x), dried with anhydrous Na_2SO_4, filtered over cotton and concentrated under reduced pressure. Purification by preparative TLC (silica gel, DCM/MeOH = 20/1) gave **8a** as a white solid in 56% yield (12.4 mg, 61 µmol). The product was obtained as an inseparable mixture of diastereomers. Full retention of the compound's stereochemistry was confirmed *via* HPLC using a chiral stationary phase.

(7*R*)-7-Phenyl-1-oxa-5-azaspiro[2.5]octan-6-one (8a): white solid; *d.r.* = 80/20. *e.r.* = 97/3 (both diast). m.p. = 162–165 °C. TLC (silica gel, DCM/MeOH = 20/1): 0.17 (UV). $[\alpha]_D^{20}$ (*c* 0.95, $CHCl_3$) = -17.1°. ^1H NMR (300 MHz, *δ*, $CDCl_3$, 298 K): 7.38–7.24 (m, 5H), 6.92 and 6.85 (bs, 1H), 3.91 and 3.80 (*major*: dd, *J* = 11.5, 5.7 Hz; *minor*: dd, *J* = 9.0, 6.5 Hz; 1H), 3.89–3.85 and 3.49 (*major*: m; *minor*: dd, *J* = 12.9, 1.6 Hz; 1H). 3.42 and 3.16–3.11 (*minor*: dd, *J* = 12.9, 2.0 Hz; *major*: m; 1H), 2.85–2.81 (m, 2H), 2.47 and 2.33 (*major*: dd, *J* = 13.7, 11.5 Hz; *minor*: dd, *J* = 13.9, 9.0 Hz; 1H), 2.22 and 1.91 (*minor*: ddd, *J* = 13.9, 6.5, 0.5 Hz; major: ddd, *J* = 13.7, 5.7, 1.6 Hz; 1H). ^{13}C NMR (75 MHz, *δ*, $CDCl_3$, 298 K): 173.0, 172.9, 139.6, 139.4, 128.8 (2), 128.5, 128.4, 127.3 (2), 54.5, 54.4, 52.9, 52.3, 48.5, 47.7, 47.0, 46.3, 36.5, 36.0. HPLC (YMC Chiral ART Cellulose-SB, eluent: *n*-hexane:*i*-PrOH = 1/1, 1.0 mL·min^{-1}, 10 °C, λ = 210 nm) retention times: t_{R1} = 10.0 min (*major* diast.), t_{R2} = 15.2 min (*minor* diast.), t_{R3} = 18.4 min (*minor* diast.), t_{R4} = 21.2 min (*major* diast.). HRMS (ESI): calcd *m/z* for $C_{12}H_{14}NO_2^+$: 204.1019 $[M+H]^+$; found: 204.1021.

5. Single Crystal X-Ray Diffraction

Single crystals suitable for single crystal X-ray diffraction were obtained by recrystallisation from CH_2Cl_2/MeOH. Single-crystal structure analysis was carried out at room temperature on a Bruker D8 Quest ECO diffractometer with graphite-monochromated MoKα radiation (λ = 0.71073 Å). The structures were solved by direct methods (SHELXT-2018/2[11]) and refined by full-matrix least-squares on F^2 (SHELXL-2019/1[12]). The H atoms were calculated geometrically, and a riding model was applied in the refinement process. Crystallographic details for **3f** can be found in Table 8. CCDC 2291222 contain the supplementary crystallographic data. This information can be obtained free of charge *via* https://www.ccdc.cam.ac.uk/structures/.

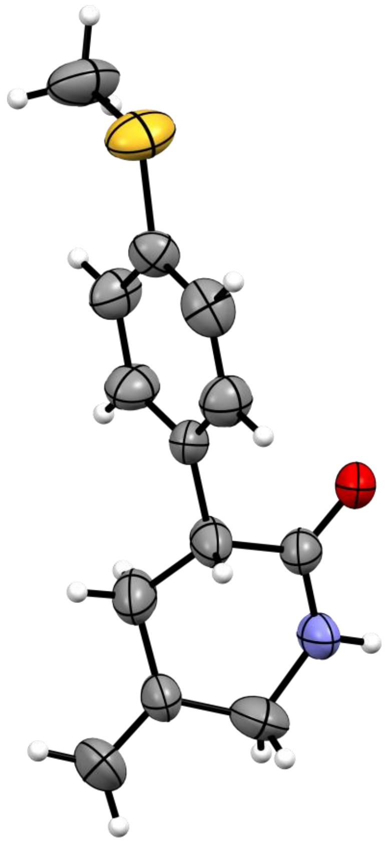

Figure 1 - Single crystal structure of **3f**.

Table 8 – Crystal data for the structure of **3f**.

Compound	**3f**
Empirical formula	$C_{13}H_{15}NOS$
Formula weight	233.32
Crystal system	monoclinic
Space group	$P2_1$
Temp/K	293
a (Å)	9.863(10)
b (Å)	5.434(6)
c (Å)	22.48(2)
α (°)	90
β (°)	94.074(15)
γ (°)	90
V (Å^3)	1202(2)
Z	4
D_{calc} (g/cm^3)	1.289
Reflns collected	28434
Indep. reflns	4308
Obs. reflns [I > 2σ(I)]	1873
Param. refin./restr.	292/1
Absorption correction	multi-scan
Flack parameter	-0.03(11)
R_1	0.069
wR_2	0.132
CCDC	2291222

[11] G. M. Sheldrick, *Acta Cryst. A*, **2015,** *71*, 3–8.
[12] G. M. Sheldrick, *Acta Cryst. C*, **2015,** *71*, 3–8.

6. Copies of Product NMR Spectra

<u>**NMR spectra** of compound **1f**</u>

^{1}H NMR (300 MHz, $CDCl_3$, 298 K)

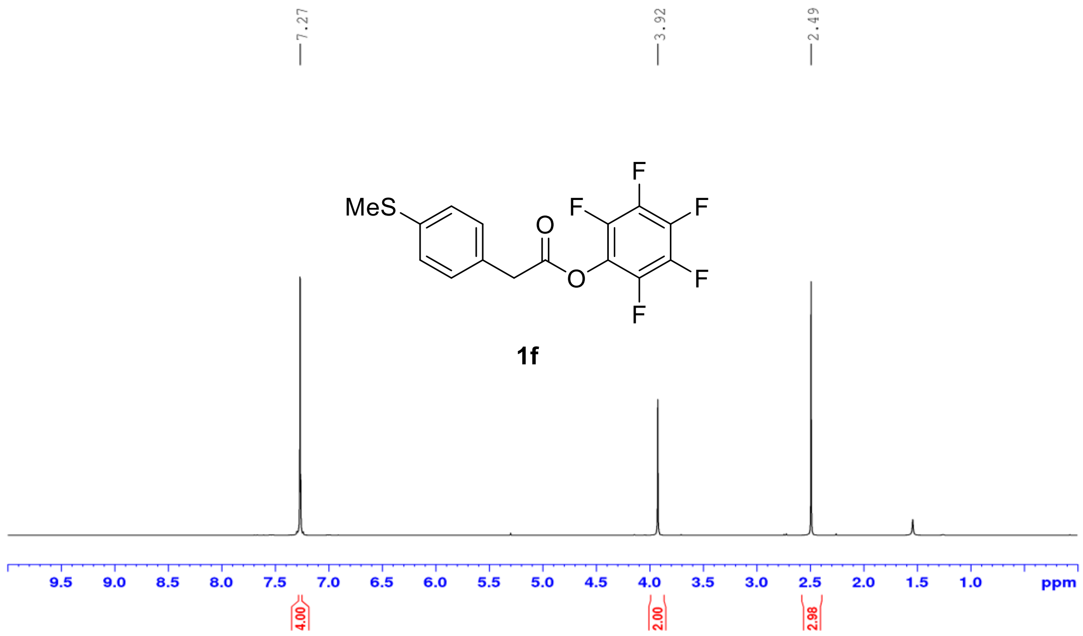

^{13}C NMR (75 MHz, $CDCl_3$, 298 K)

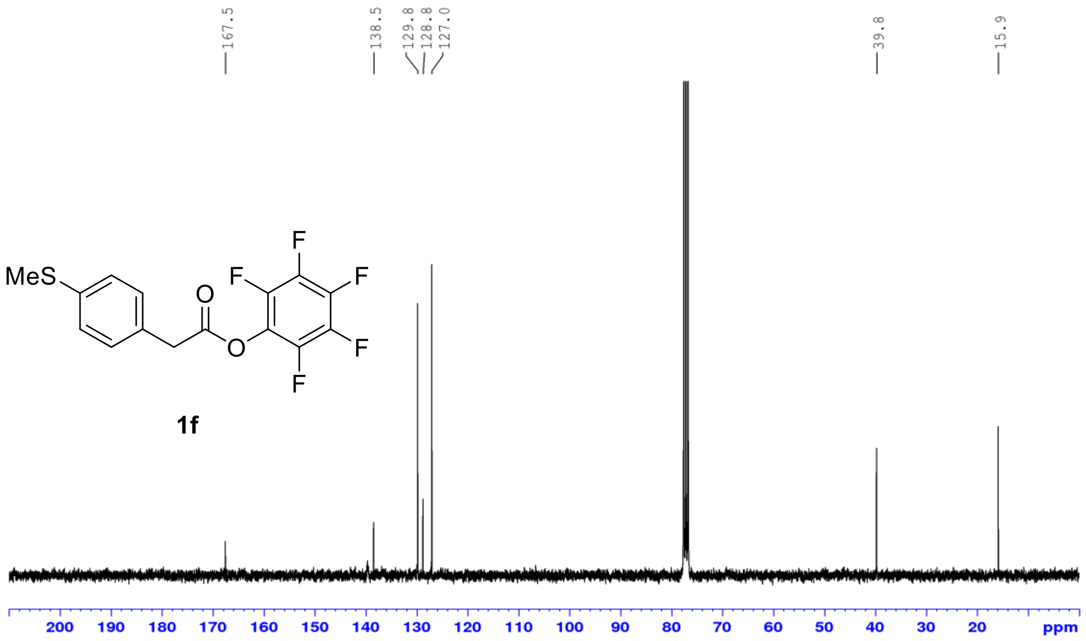

^{19}F NMR (282 MHz, $CDCl_3$, 298 K)

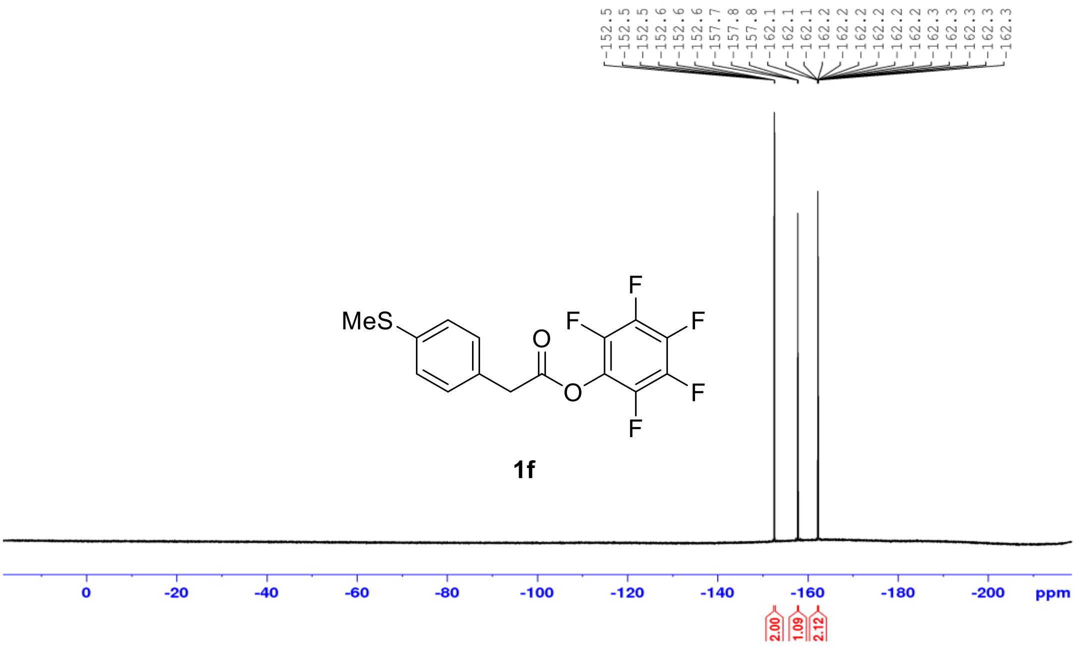

NMR spectra of compound **1p**

^{1}H NMR (300 MHz, $CDCl_3$, 298 K)

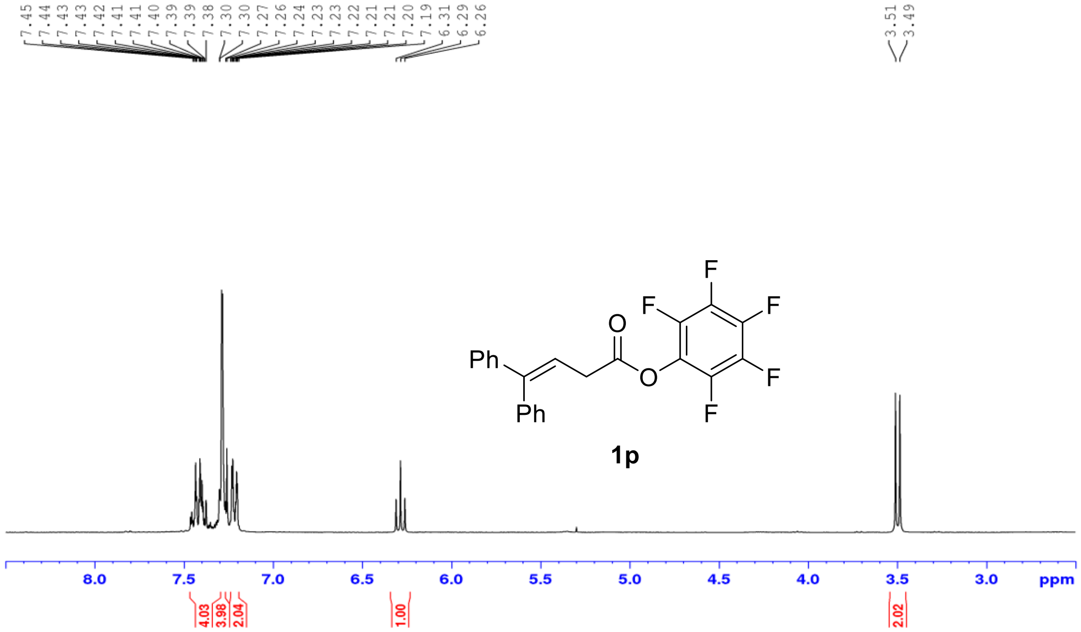

^{13}C NMR (75 MHz, $CDCl_3$, 298 K)

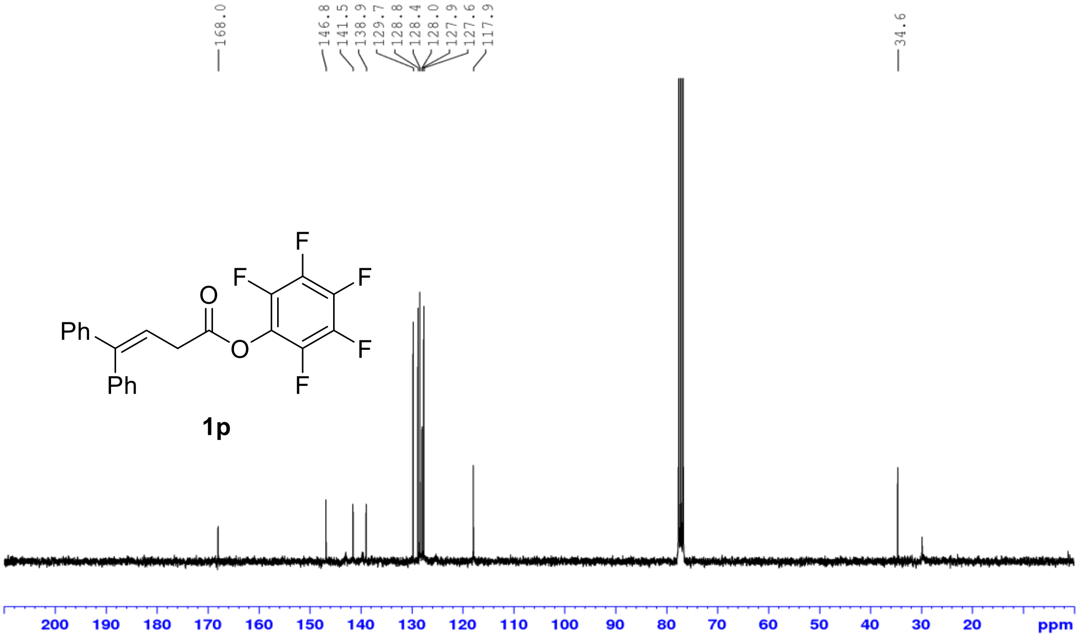

^{19}F NMR (282 MHz, $CDCl_3$, 298 K)

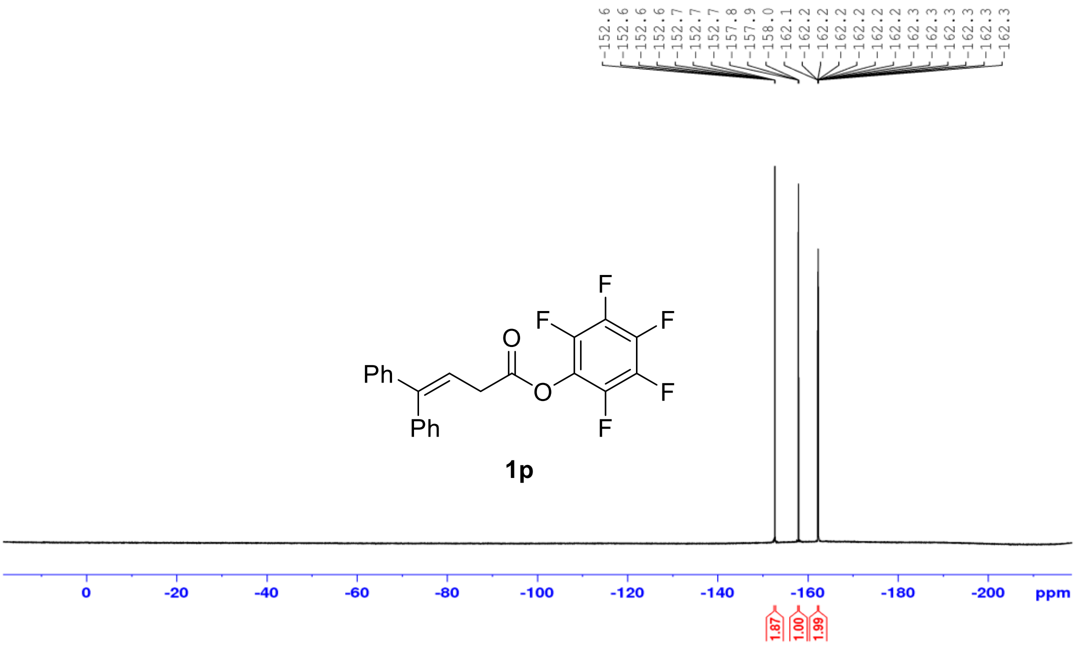

<u>**NMR spectra** of compound **2a**</u>

^{1}H NMR (300 MHz, $CDCl_3$, 298 K)

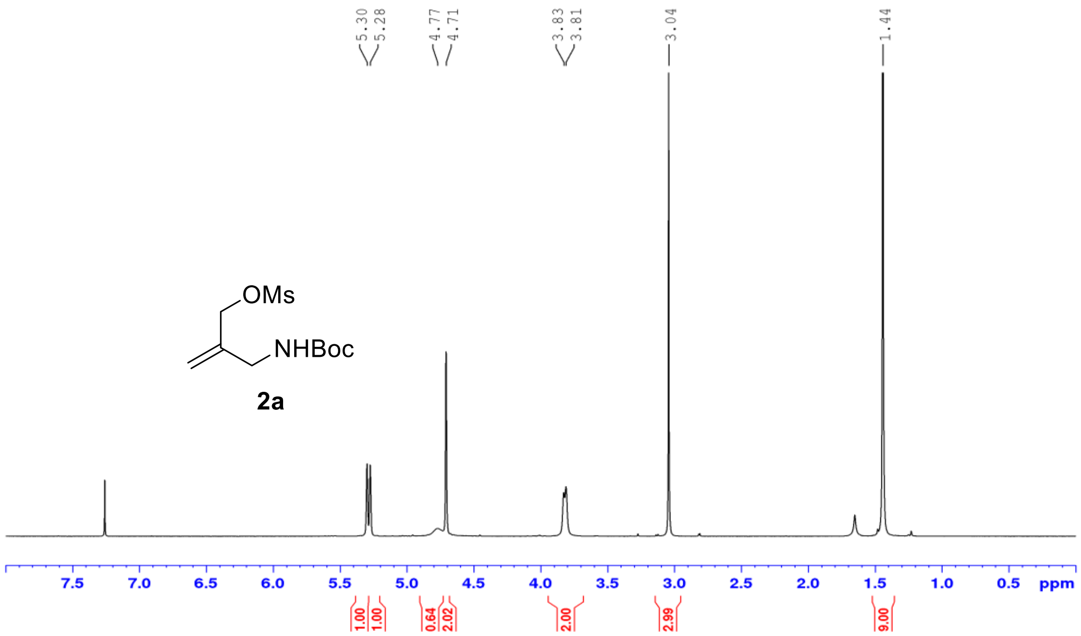

^{13}C NMR (75 MHz, $CDCl_3$, 298 K)

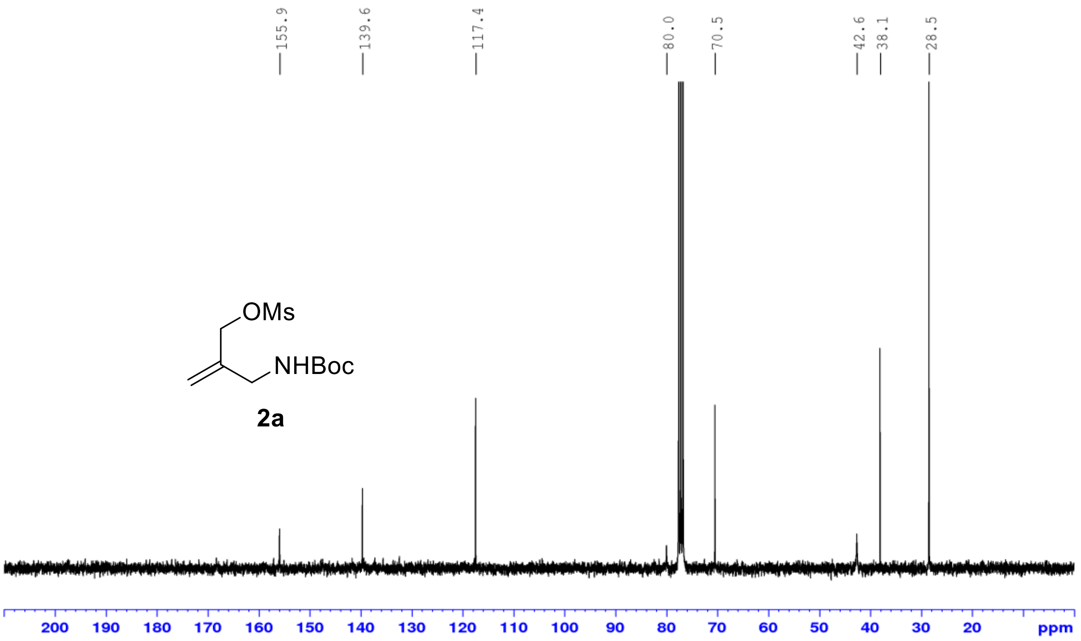

<u>**NMR spectra** of compound **2b**</u>

^{1}H NMR (300 MHz, $CDCl_3$, 298 K)

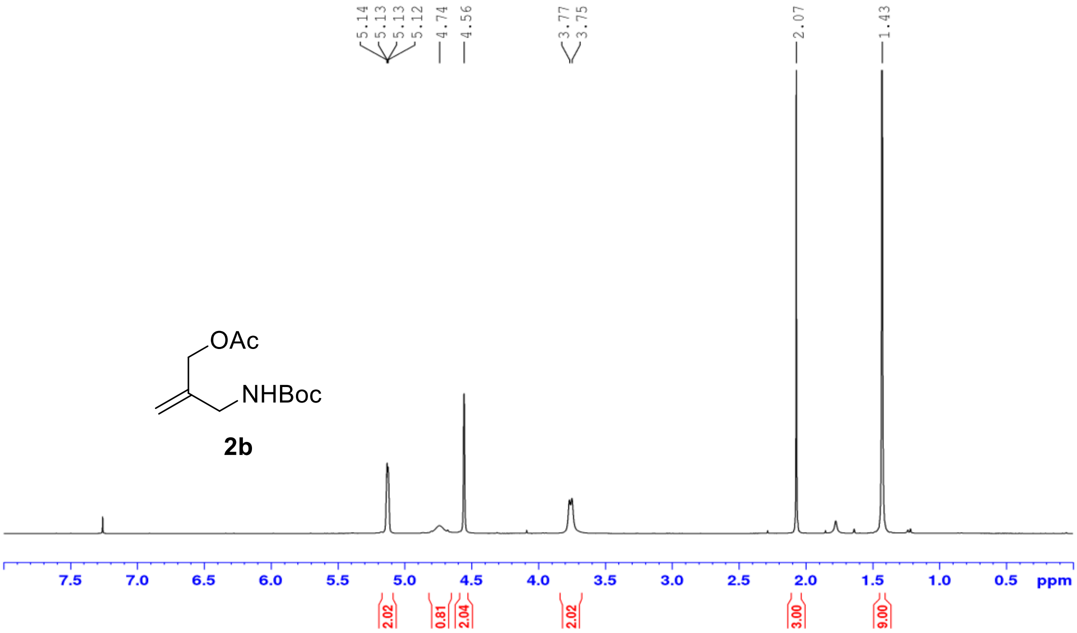

^{13}C NMR (75 MHz, $CDCl_3$, 298 K)

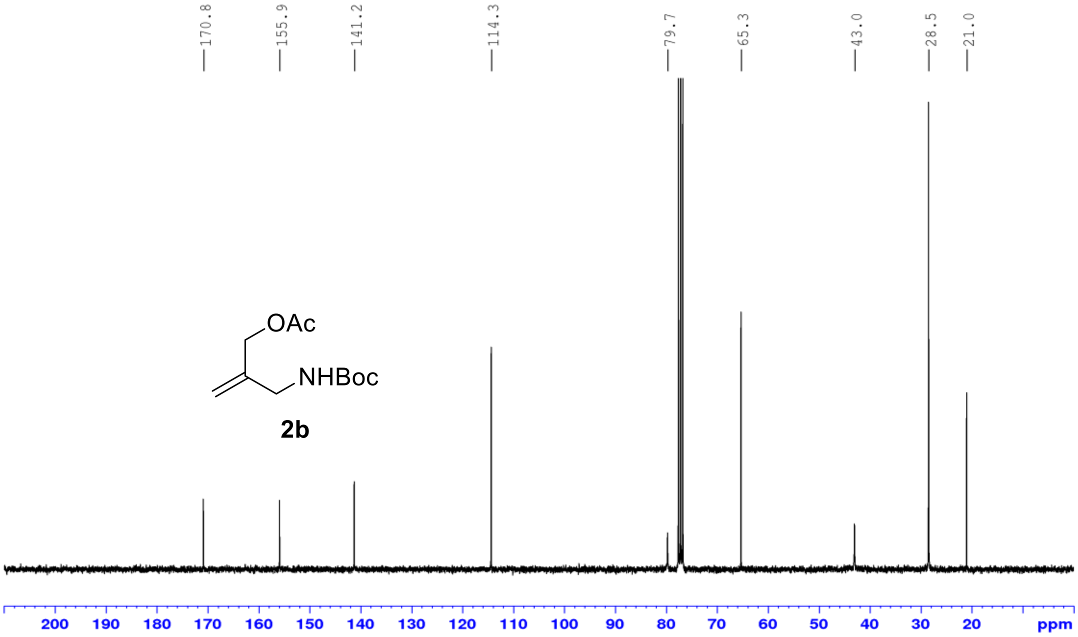

NMR spectra of compound **2c**

^{1}H NMR (300 MHz, $CDCl_3$, 298 K)

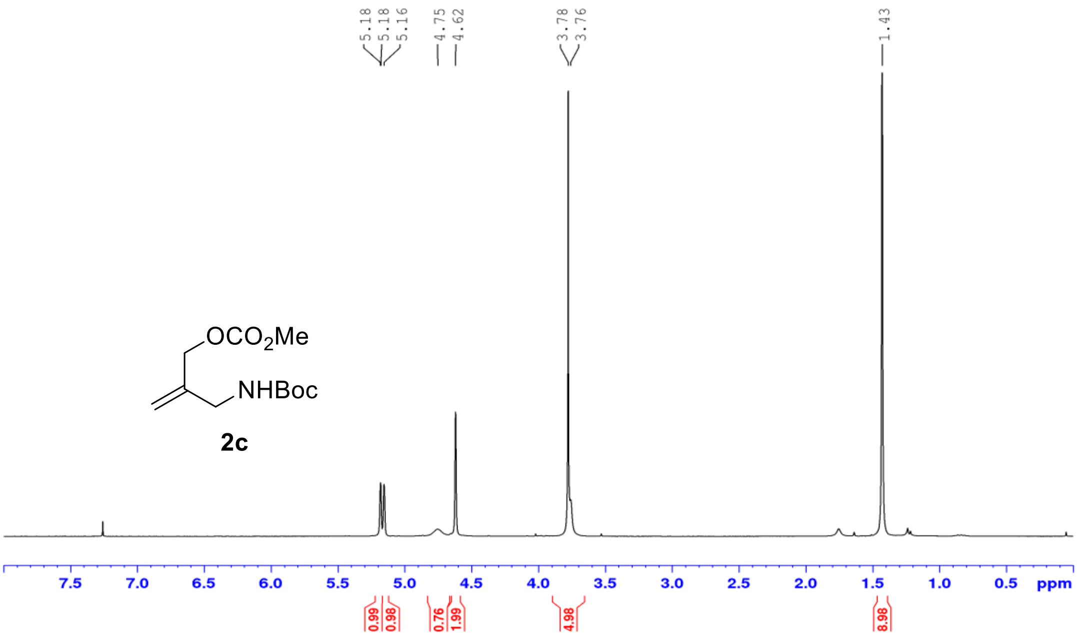

^{13}C NMR (75 MHz, $CDCl_3$, 298 K)

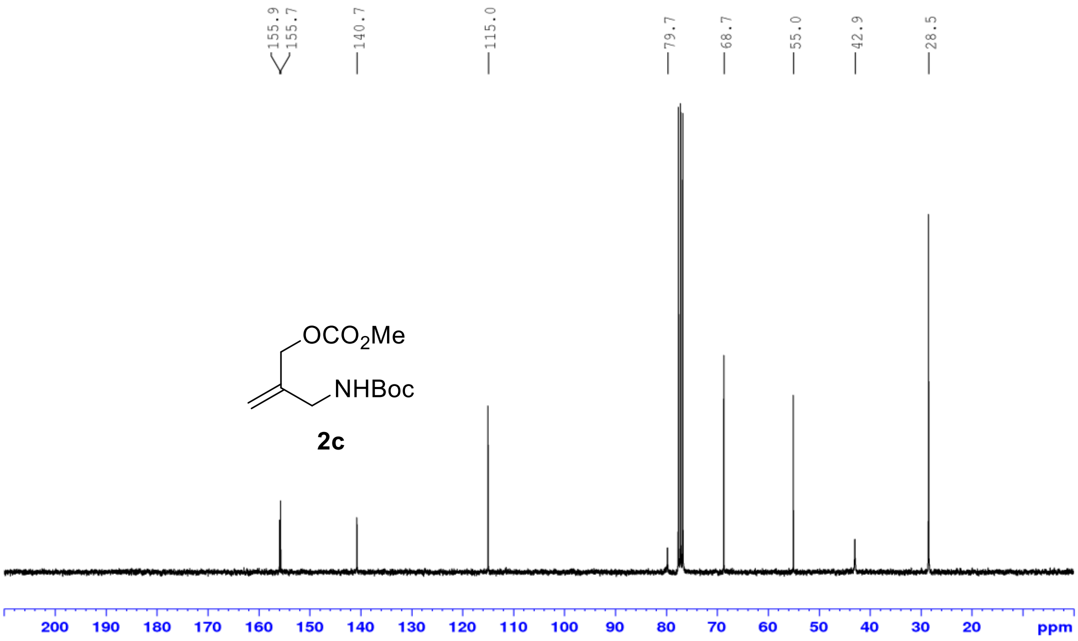

<u>**NMR spectra** of compound **2d**</u>

^{1}H NMR (300 MHz, $CDCl_3$, 298 K)

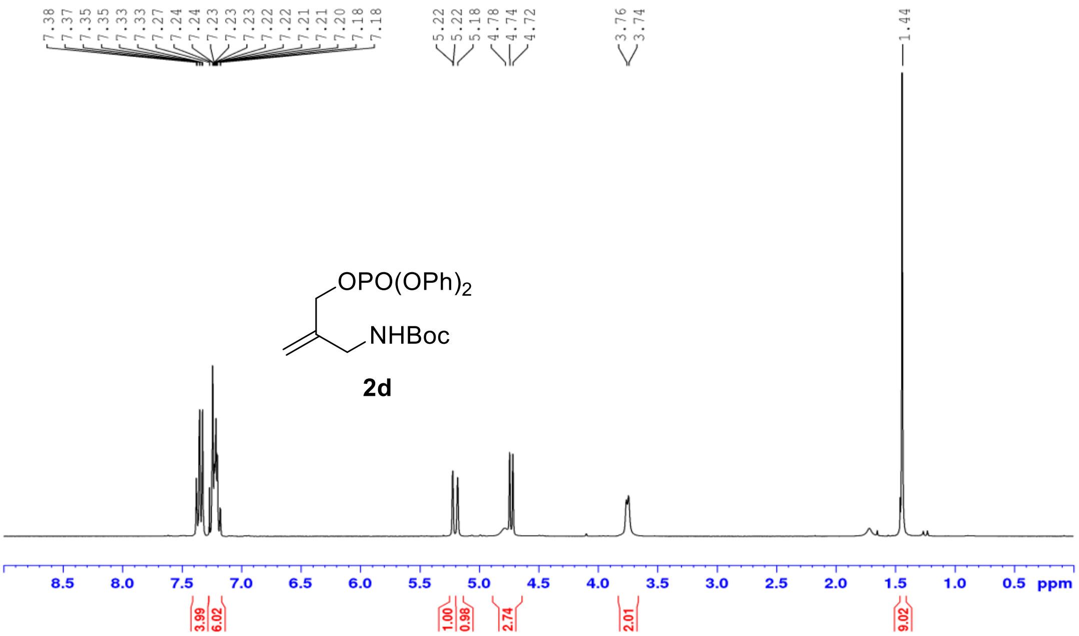

^{13}C NMR (75 MHz, $CDCl_3$, 298 K)

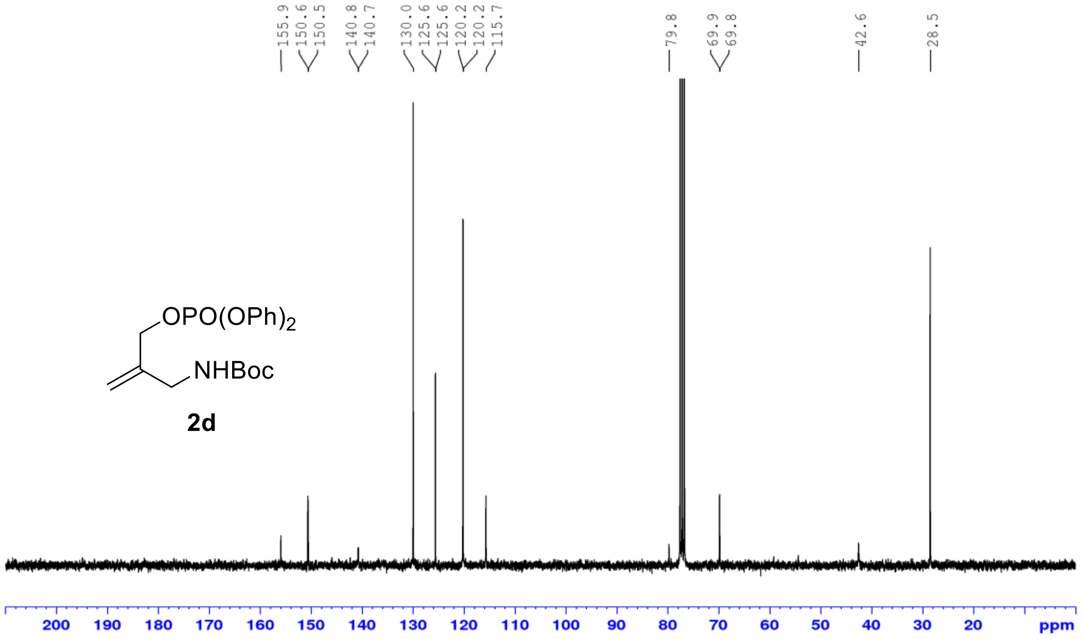

^{31}P NMR (121 MHz, $CDCl_3$, 298 K)

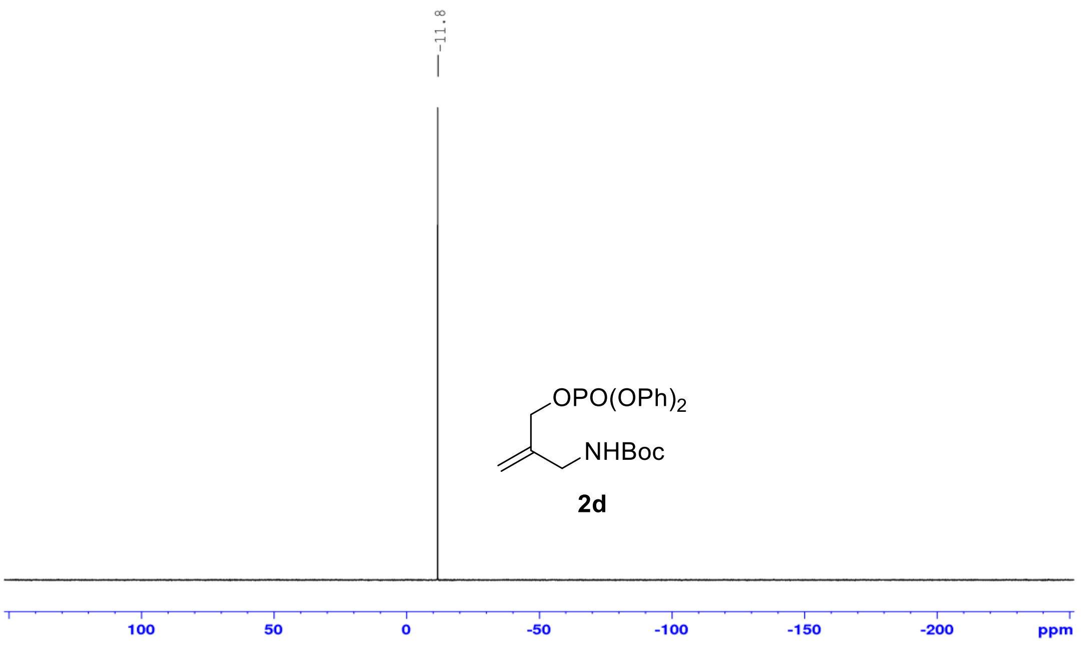

NMR spectra of compound **2e**

^{1}H NMR (300 MHz, $CDCl_3$, 298 K)

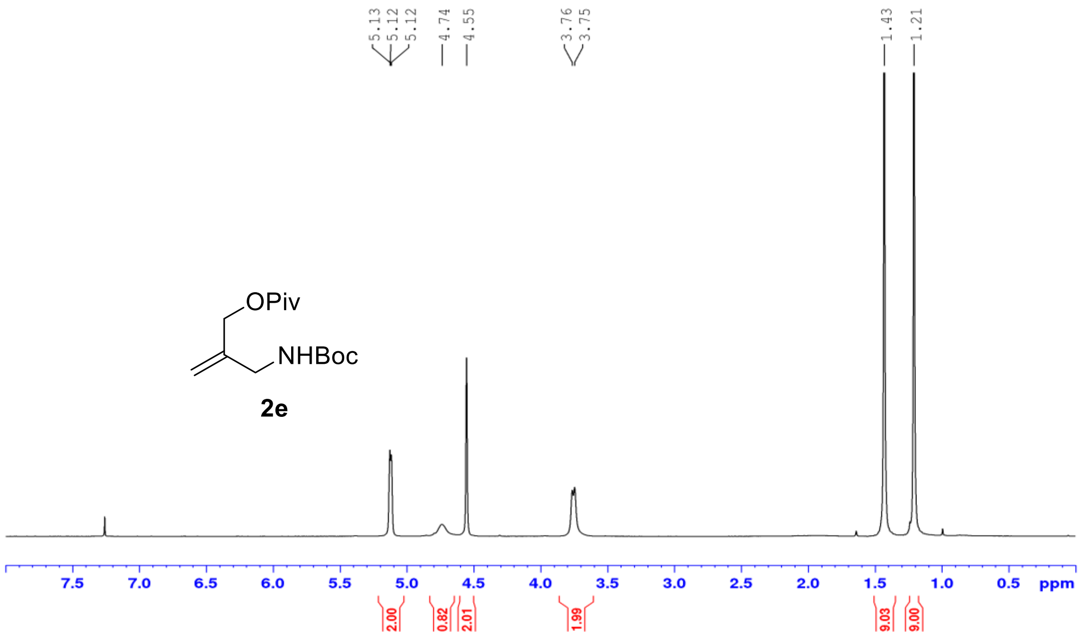

^{13}C NMR (75 MHz, $CDCl_3$, 298 K)

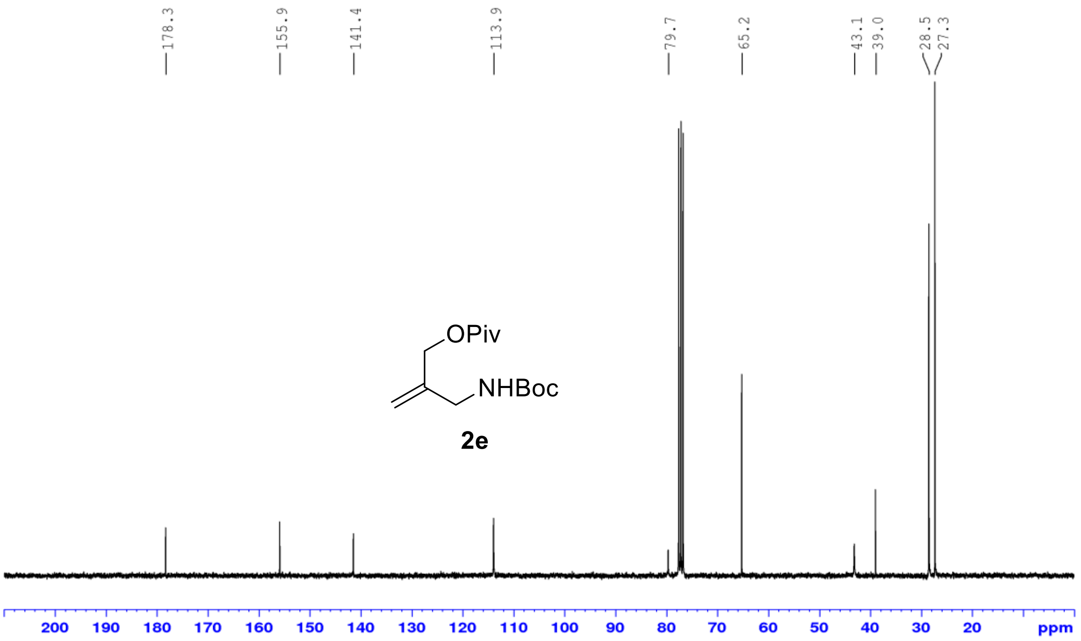

NMR spectra of compound 3a

^{1}H NMR (500 MHz, $CDCl_3$, 298 K)

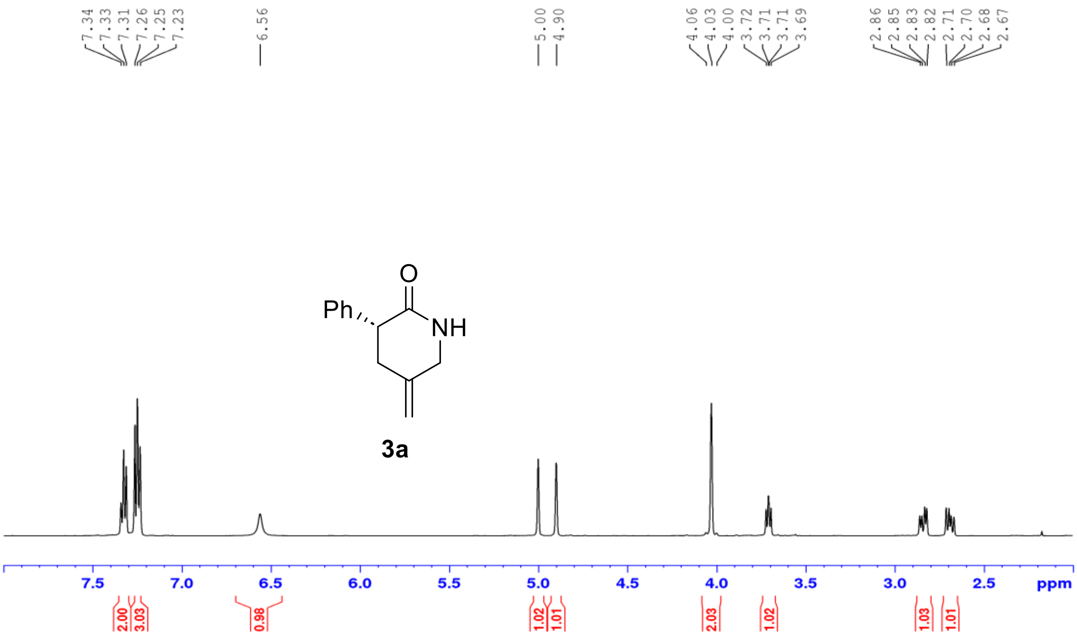

^{13}C NMR (125 MHz, $CDCl_3$, 298 K)

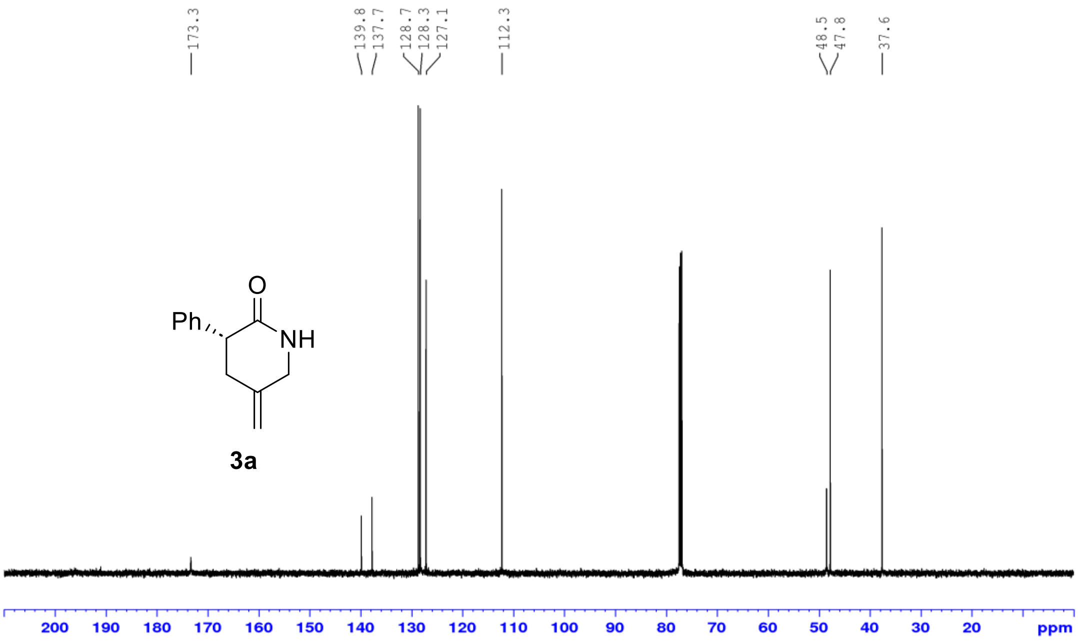

NMR spectra of compound **3b**

^{1}H NMR (300 MHz, $CDCl_3$, 298 K)

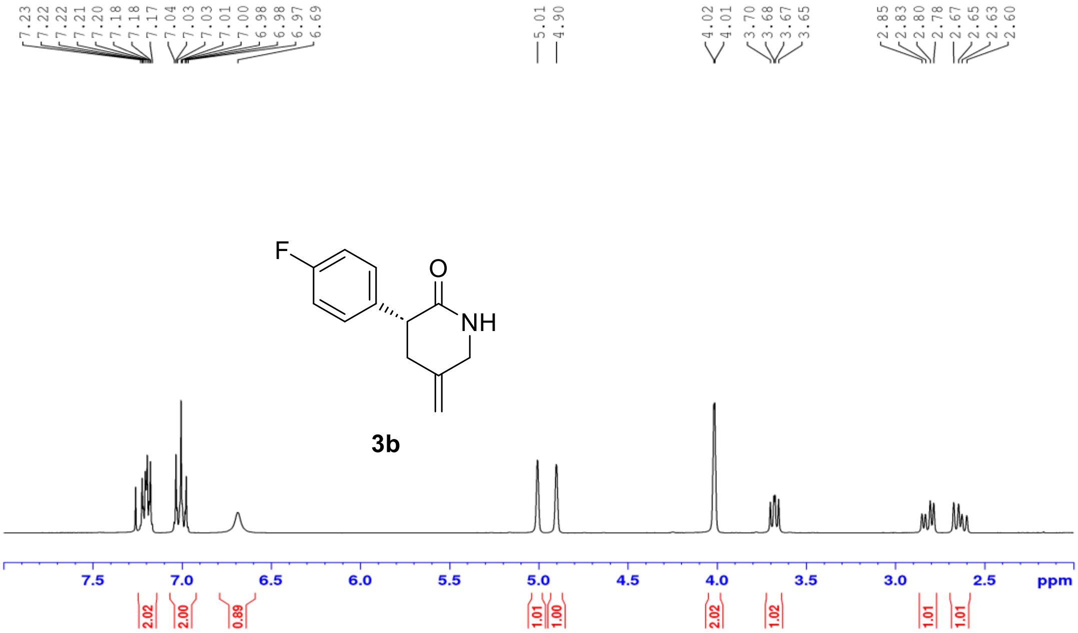

^{13}C NMR (75 MHz, $CDCl_3$, 298 K)

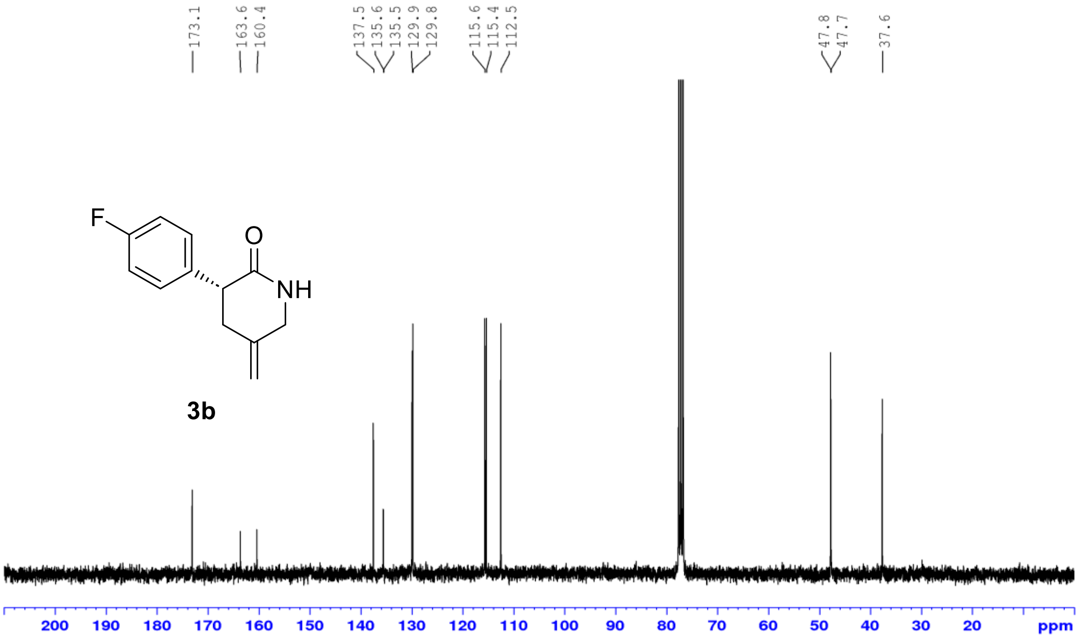

^{19}F NMR (282 MHz, $CDCl_3$, 298 K)

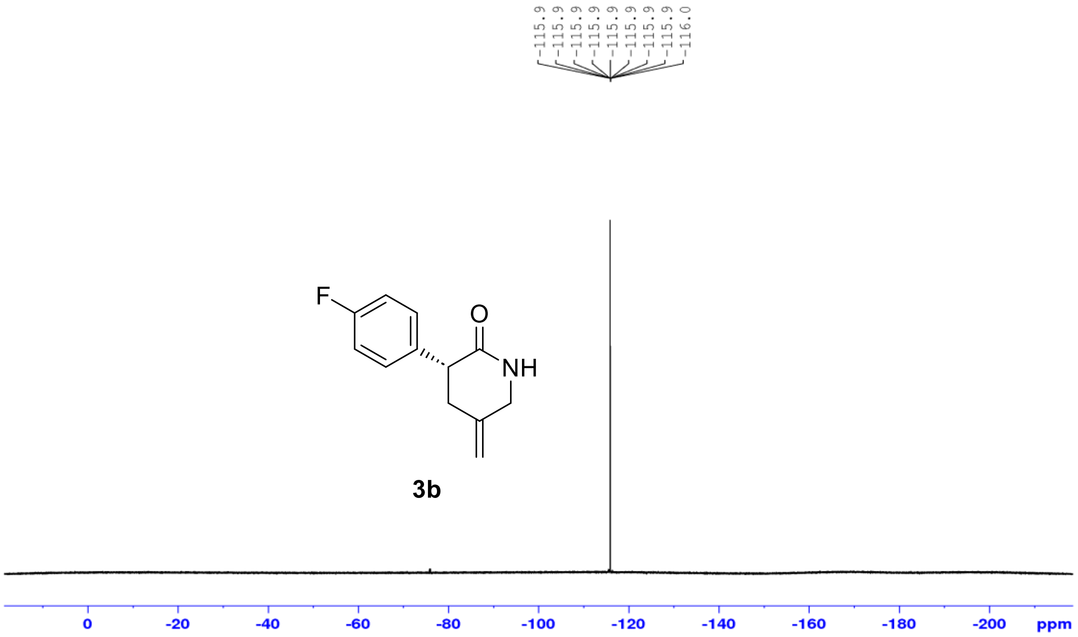

NMR spectra of compound **3c**

^{1}H NMR (300 MHz, $CDCl_3$, 298 K)

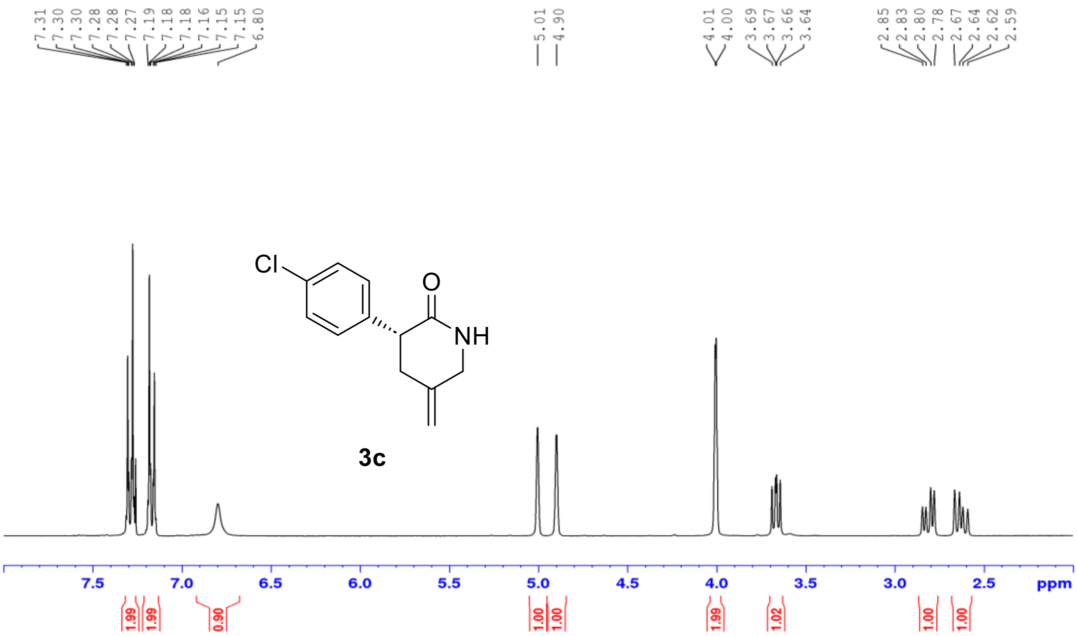

^{13}C NMR (75 MHz, $CDCl_3$, 298 K)

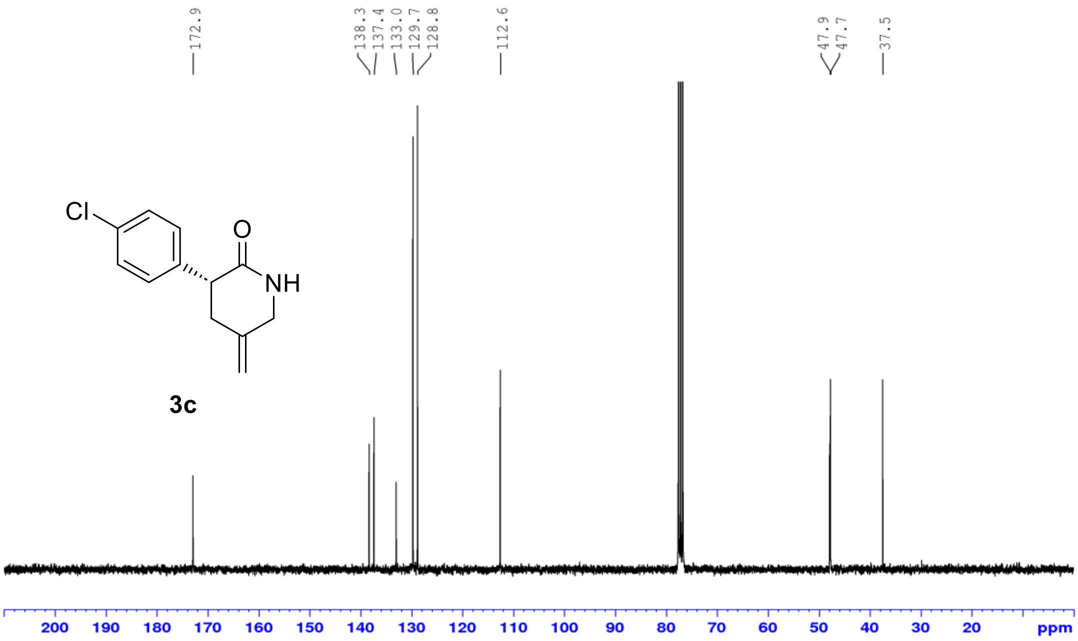

NMR spectra of compound 3d

^{1}H NMR (300 MHz, $CDCl_3$, 298 K)

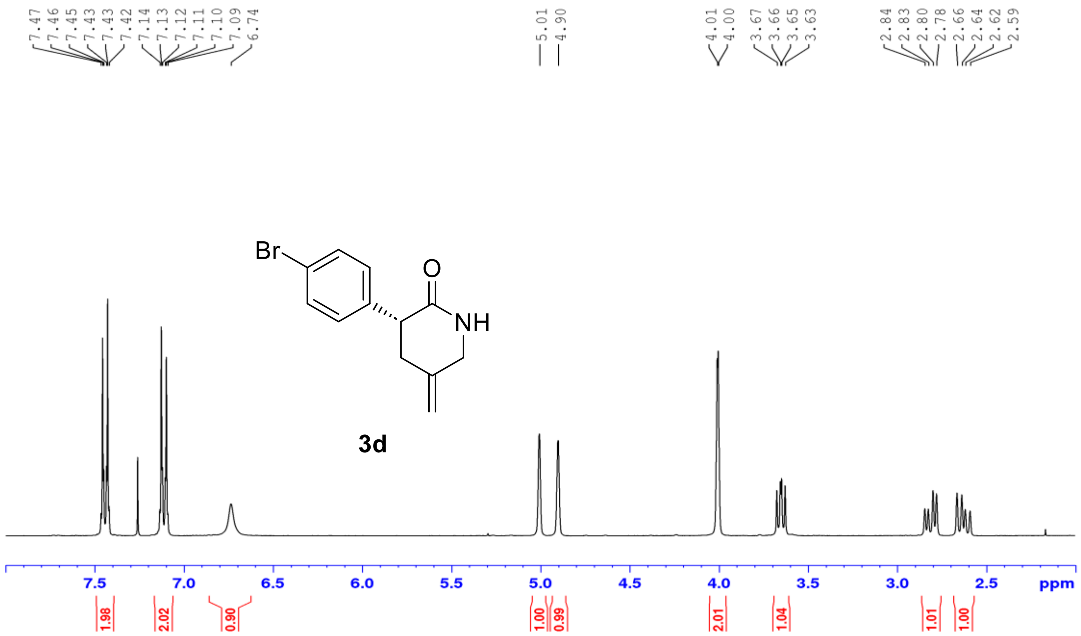

^{13}C NMR (75 MHz, $CDCl_3$, 298 K)

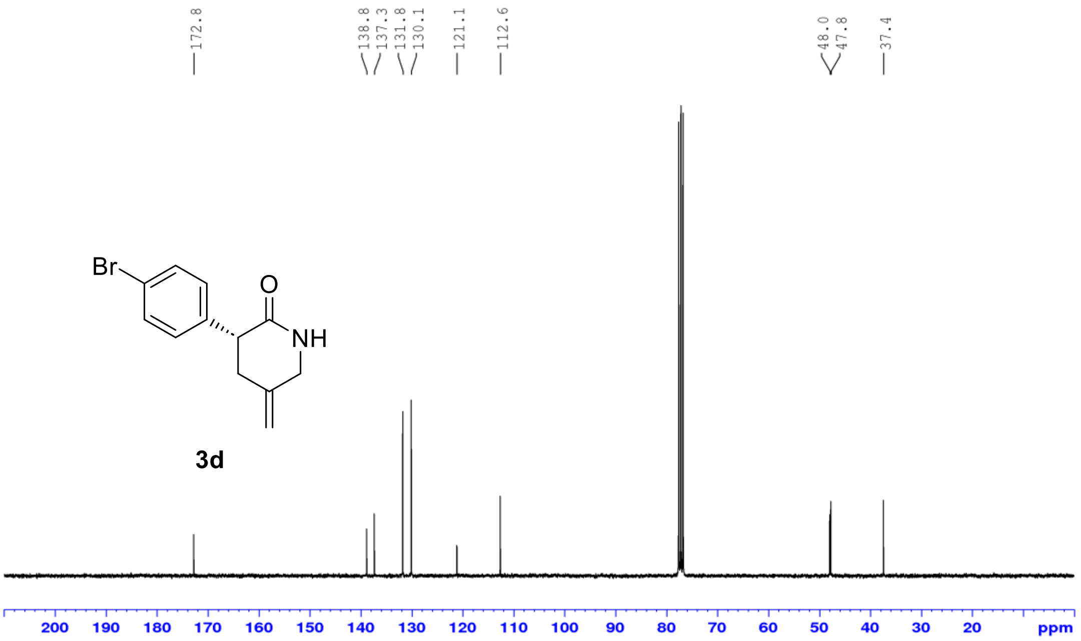

NMR spectra of compound **3e**

^{1}H NMR (300 MHz, $CDCl_3$, 298 K)

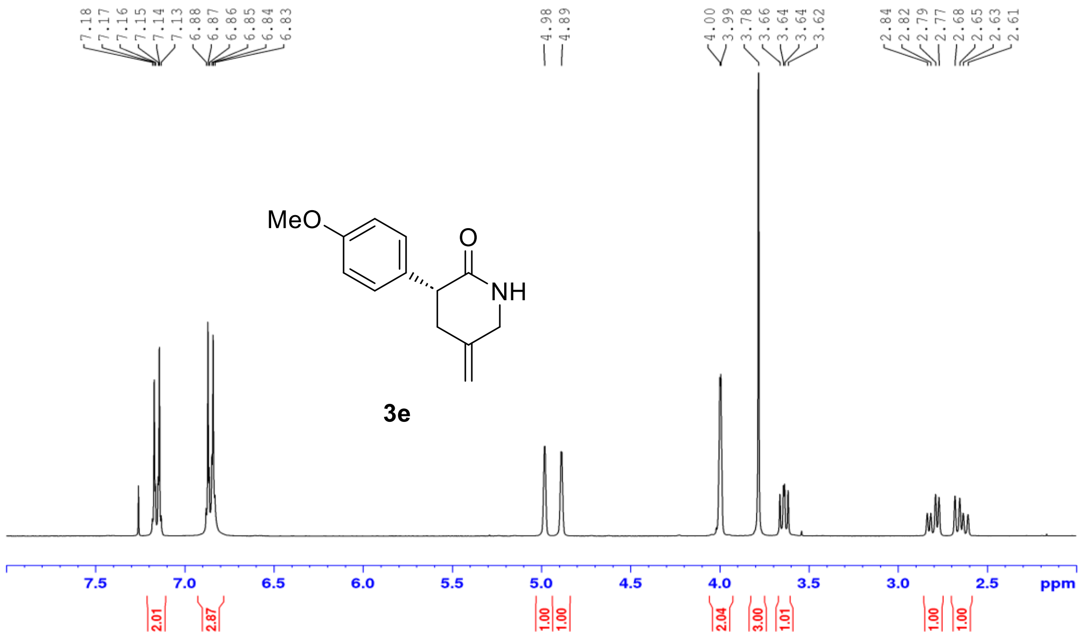

^{13}C NMR (75 MHz, $CDCl_3$, 298 K)

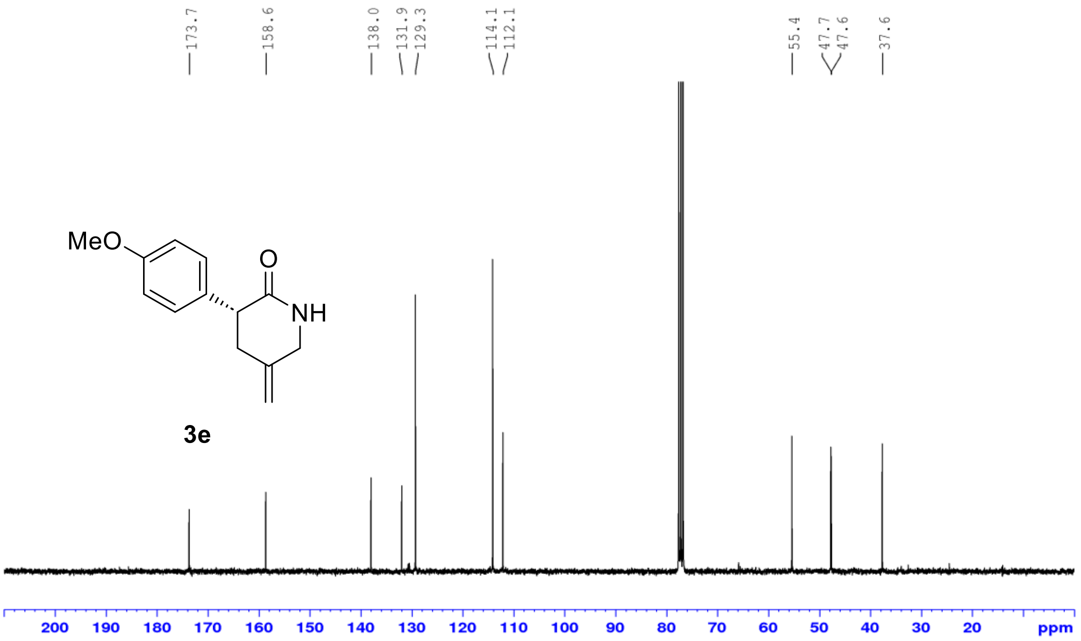

NMR spectra of compound 3f

^{1}H NMR (300 MHz, $CDCl_3$, 298 K)

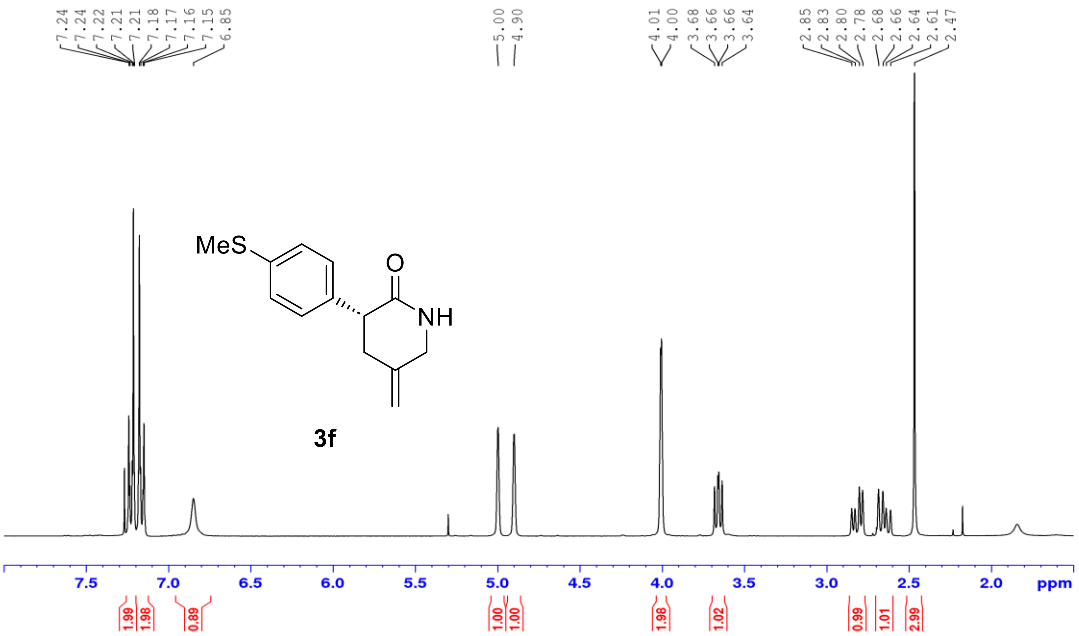

^{13}C NMR (75 MHz, $CDCl_3$, 298 K)

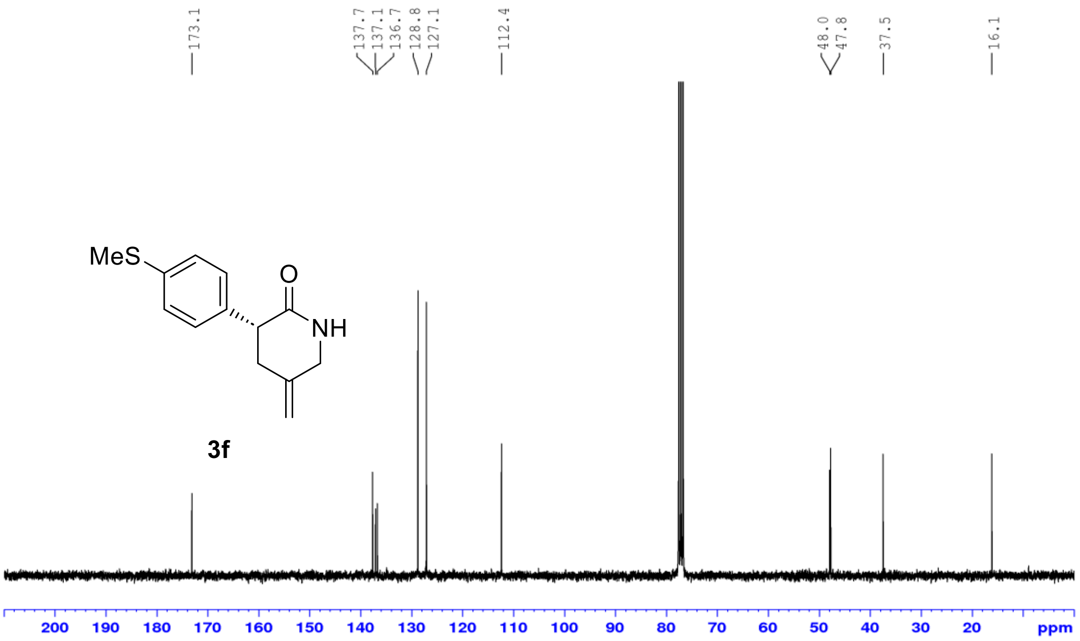

NMR spectra of compound **3g**

^{1}H NMR (300 MHz, $CDCl_3$, 298 K)

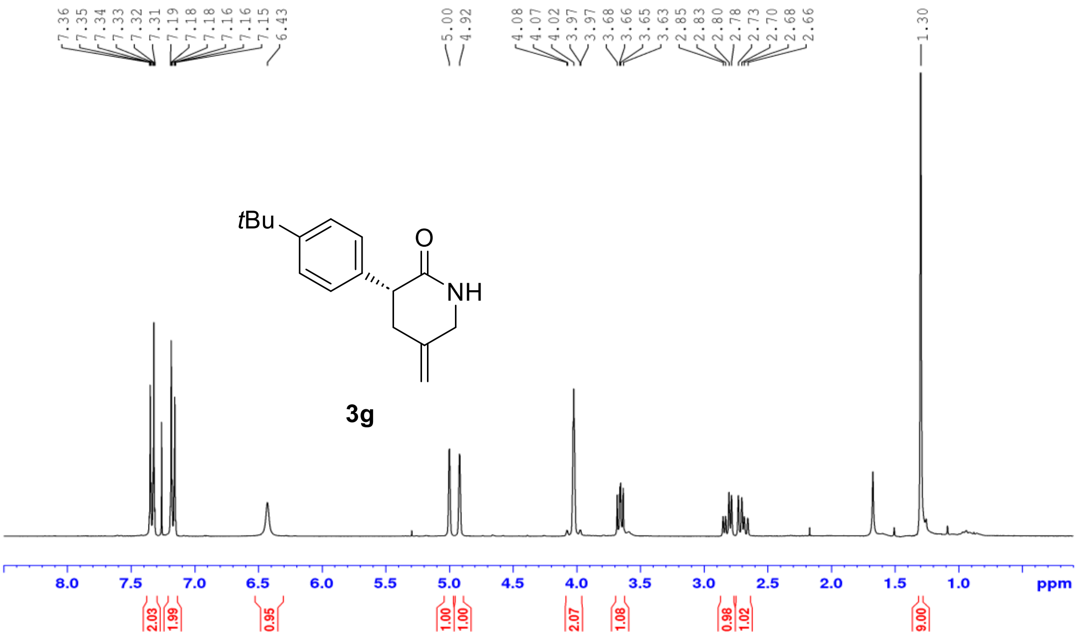

^{13}C NMR (75 MHz, $CDCl_3$, 298 K)

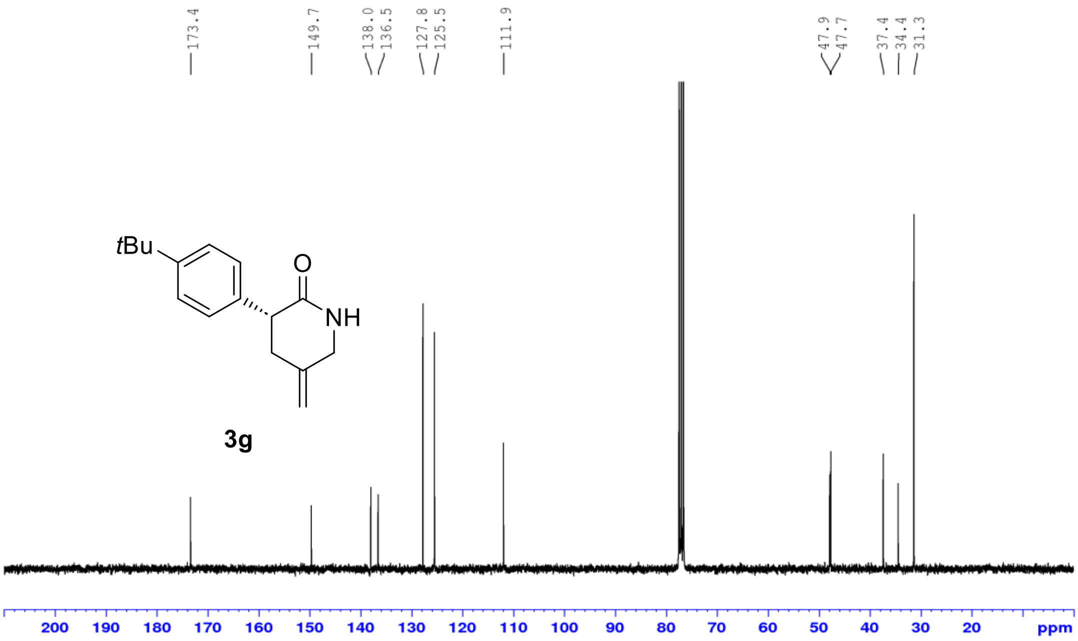

<u>**NMR spectra** of compound **3h**</u>

^{1}H NMR (300 MHz, $CDCl_3$, 298 K)

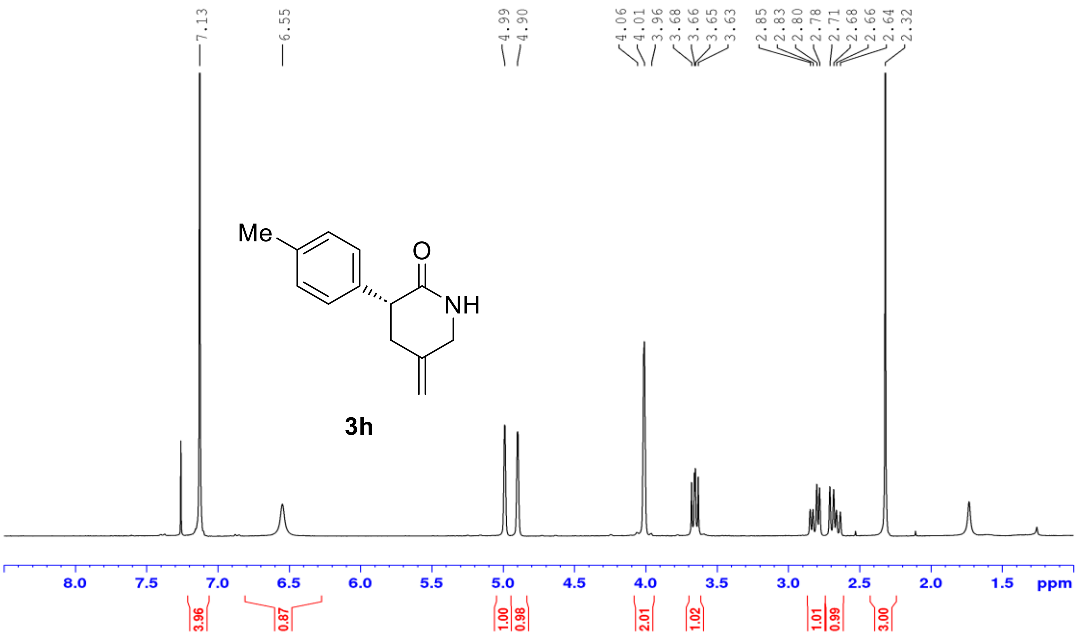

^{13}C NMR (75 MHz, $CDCl_3$, 298 K)

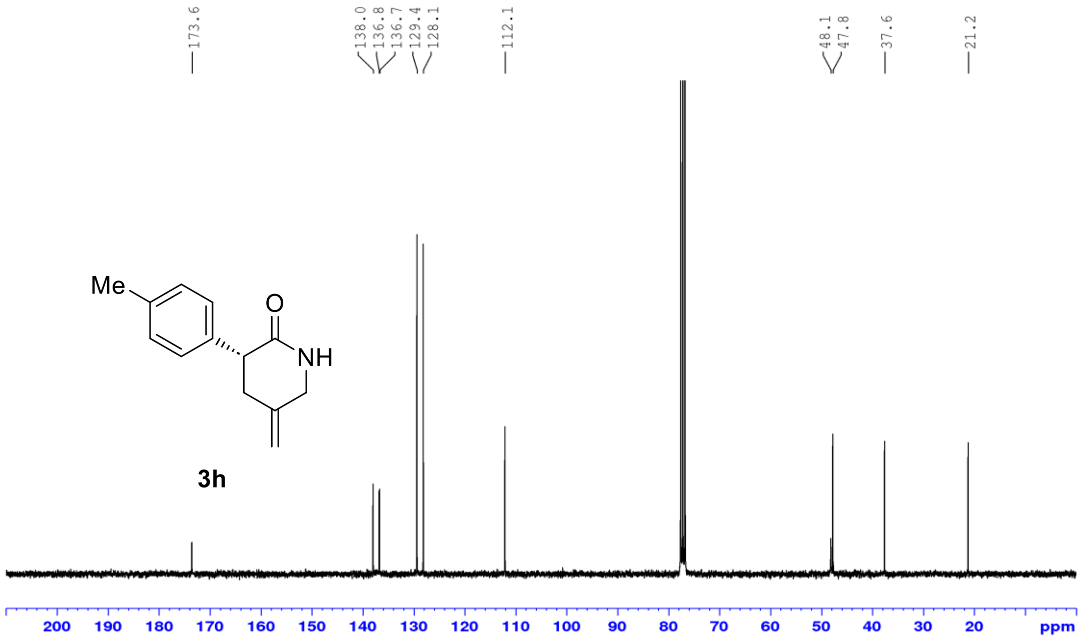

NMR spectra of compound **3i**

^{1}H NMR (500 MHz, $CDCl_3$, 298 K)

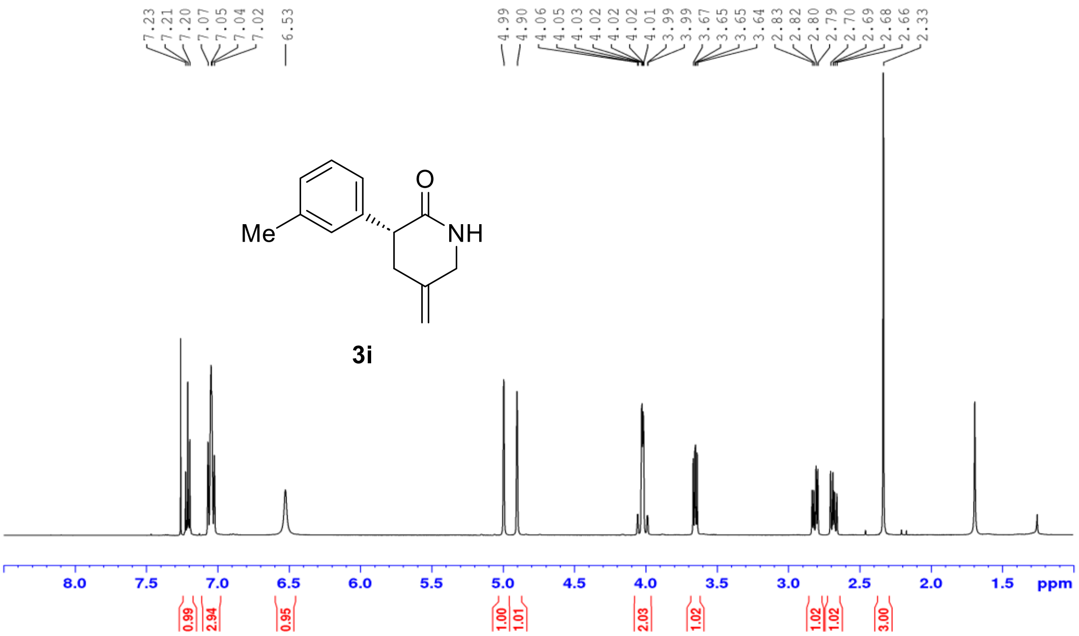

^{13}C NMR (125 MHz, $CDCl_3$, 298 K)

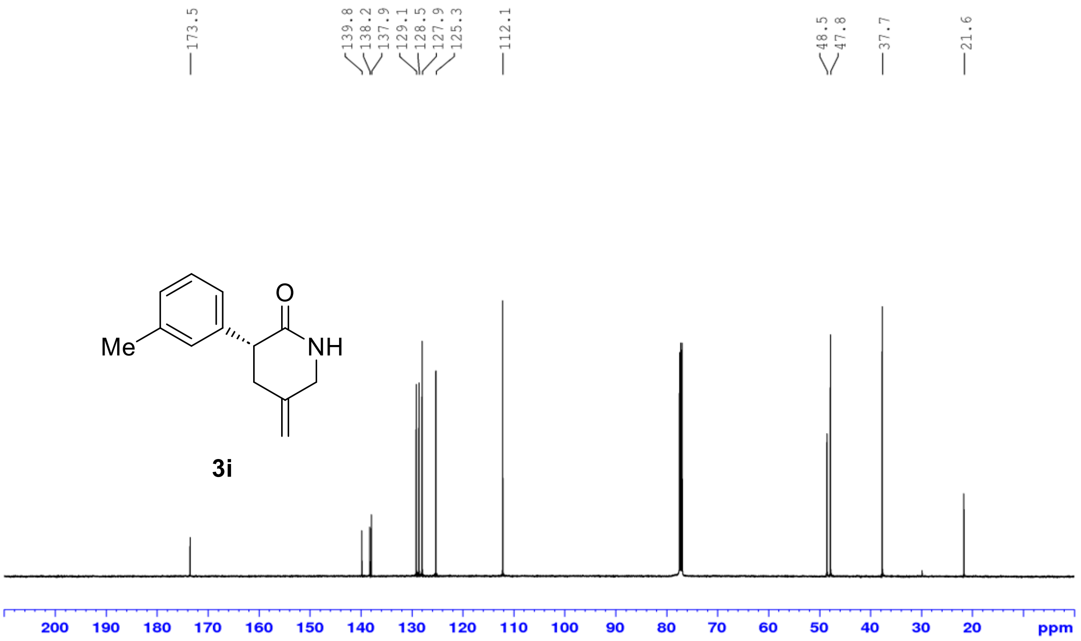

NMR spectra of compound **3j**

^{1}H NMR (500 MHz, $CDCl_3$, 298 K)

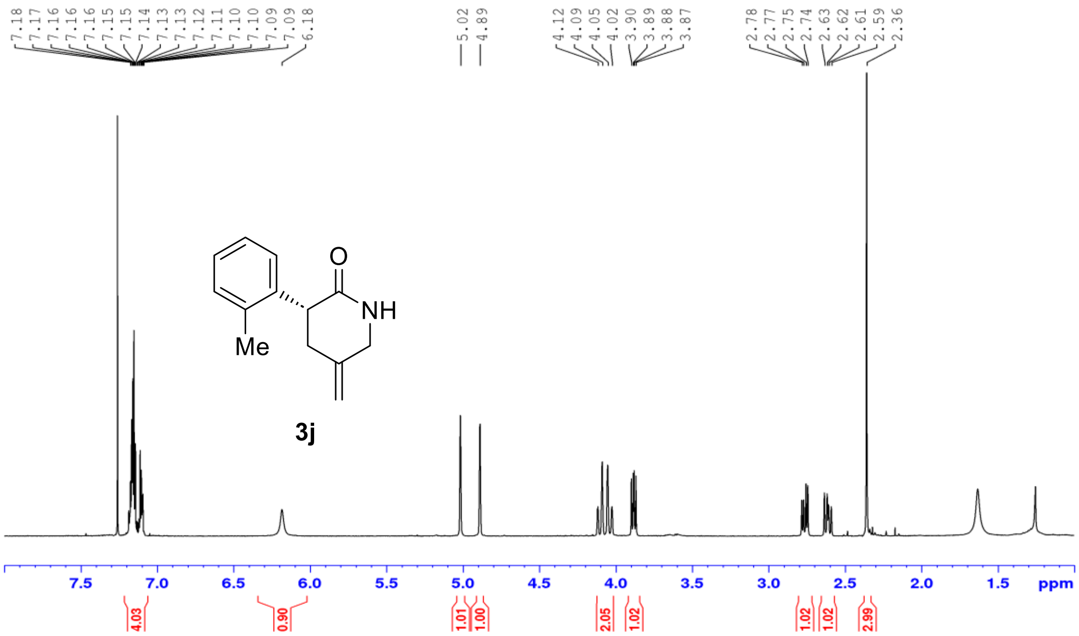

^{13}C NMR (125 MHz, $CDCl_3$, 298 K)

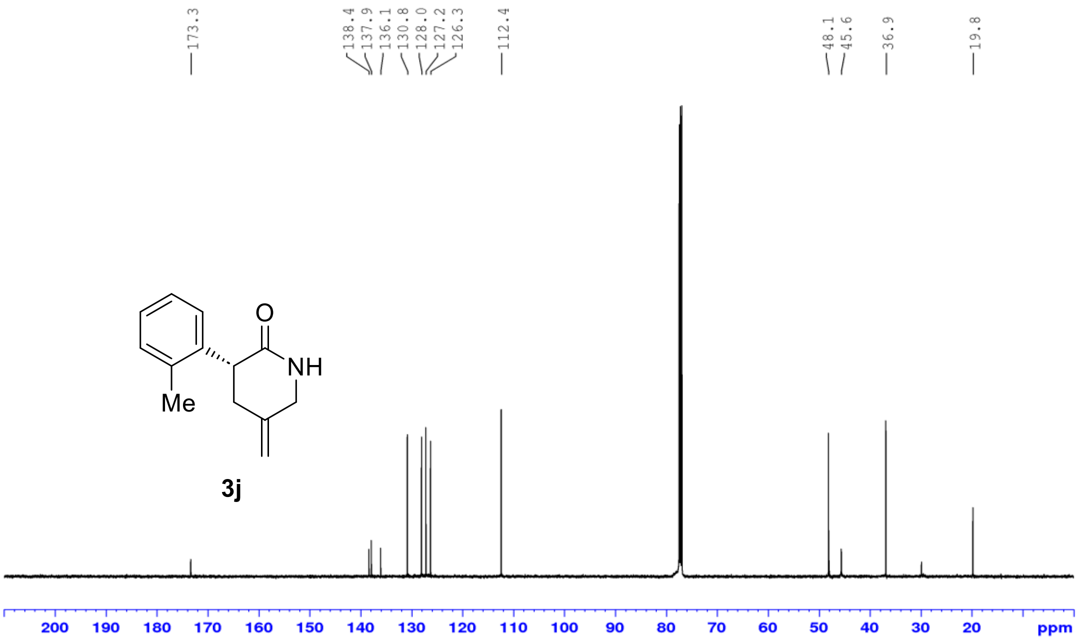

NMR spectra of compound 3k

^{1}H NMR (300 MHz, $CDCl_3$, 298 K)

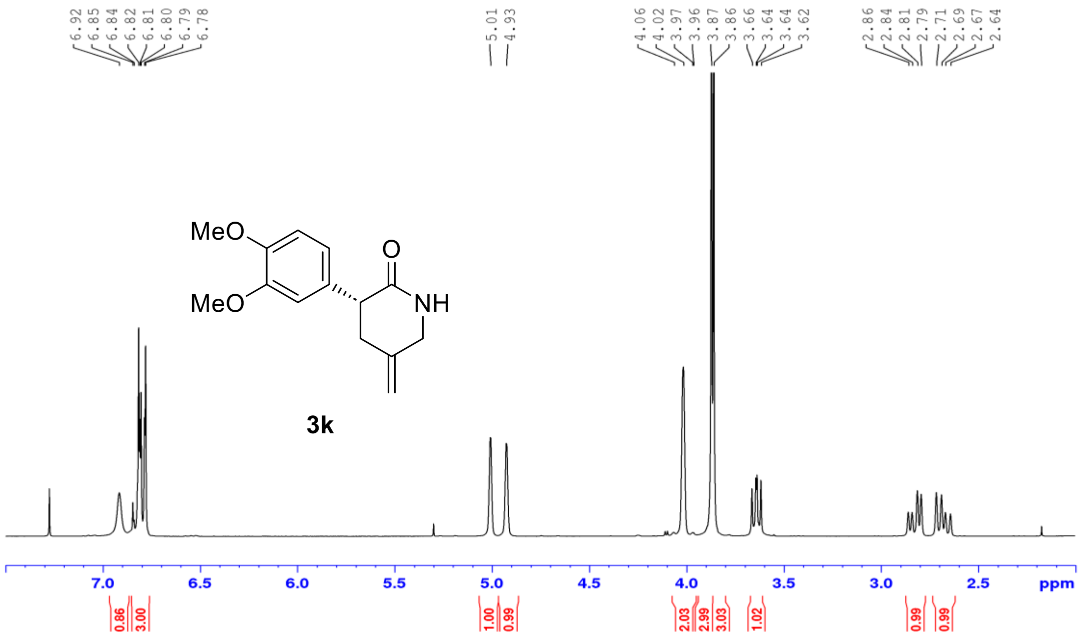

^{13}C NMR (75 MHz, $CDCl_3$, 298 K)

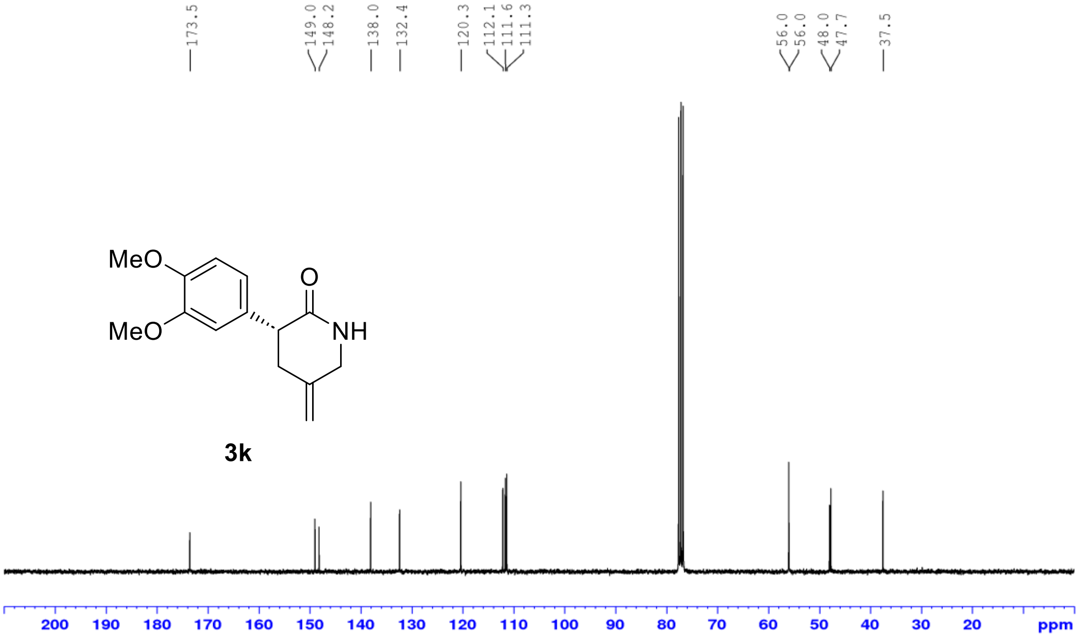

NMR spectra of compound **3l**

^{1}H NMR (500 MHz, $CDCl_3$, 298 K)

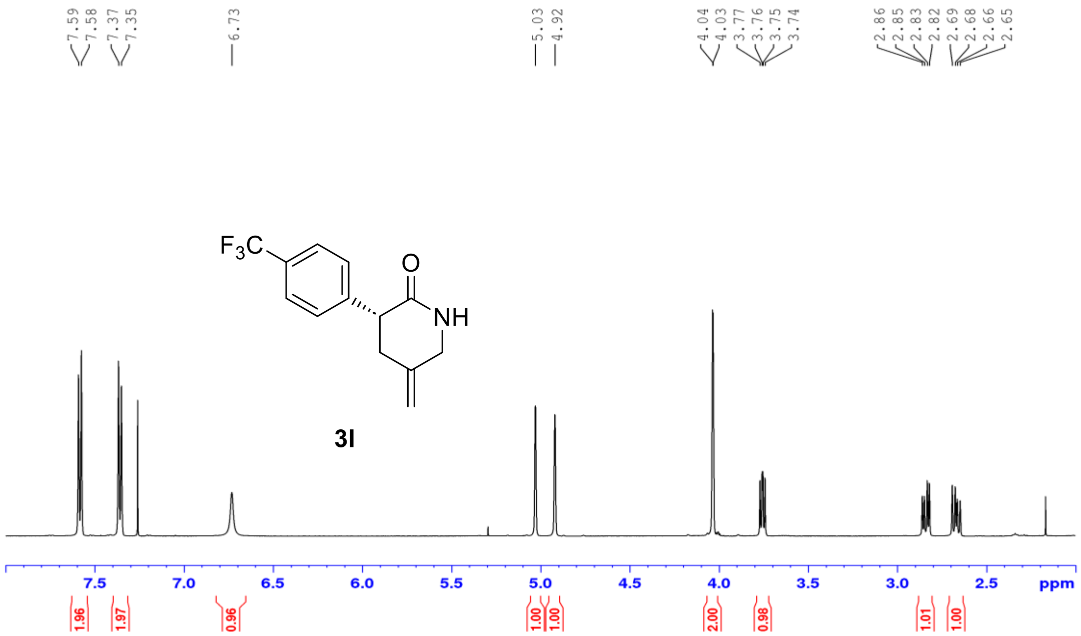

^{13}C NMR (125 MHz, $CDCl_3$, 298 K)

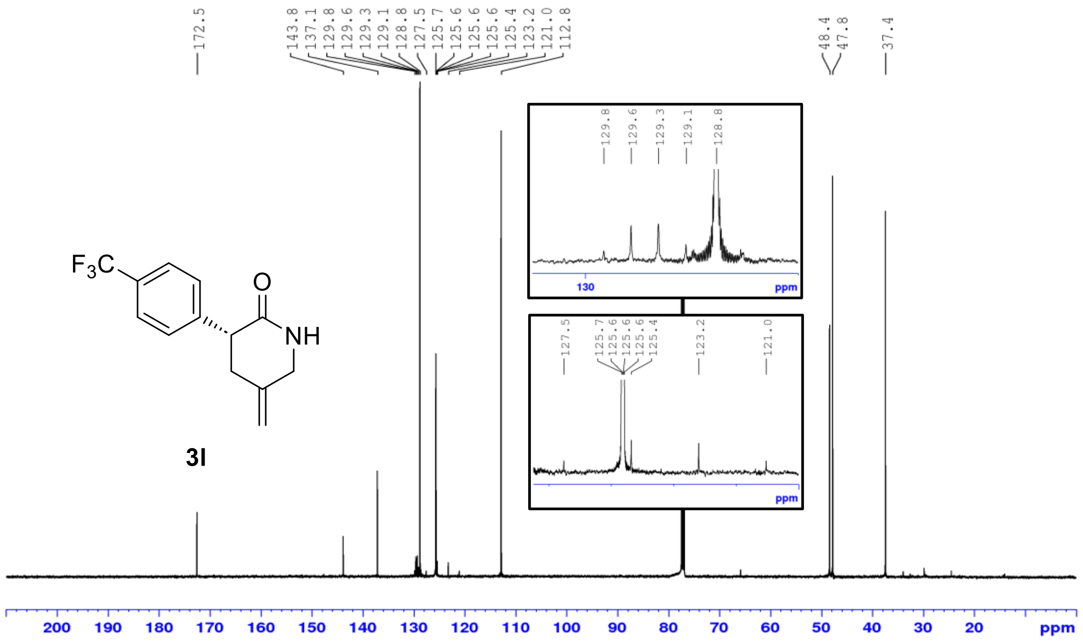

^{19}F NMR (470 MHz, $CDCl_3$, 298 K)

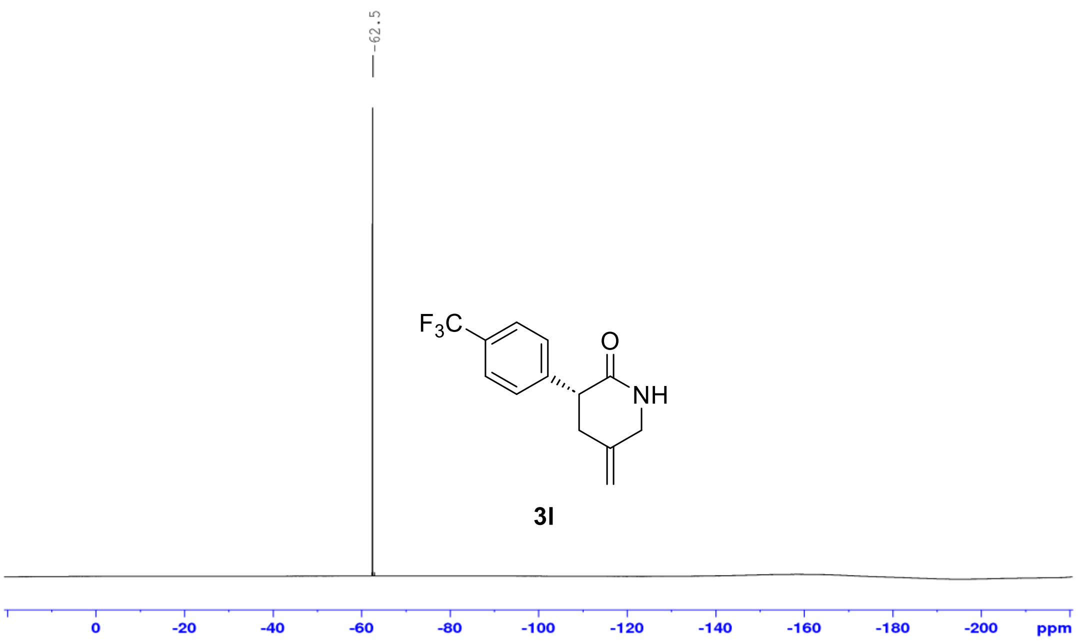

NMR spectra of compound **3m**

[1]**H NMR** (300 MHz, $CDCl_3$, 298 K)

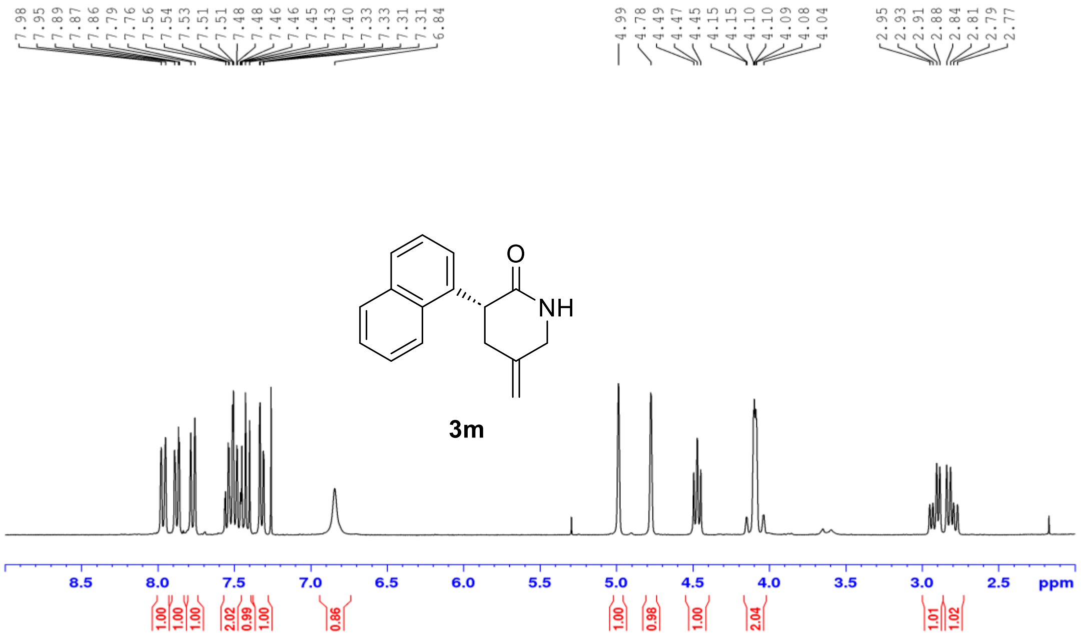

[13]**C NMR** (75 MHz, $CDCl_3$, 298 K)

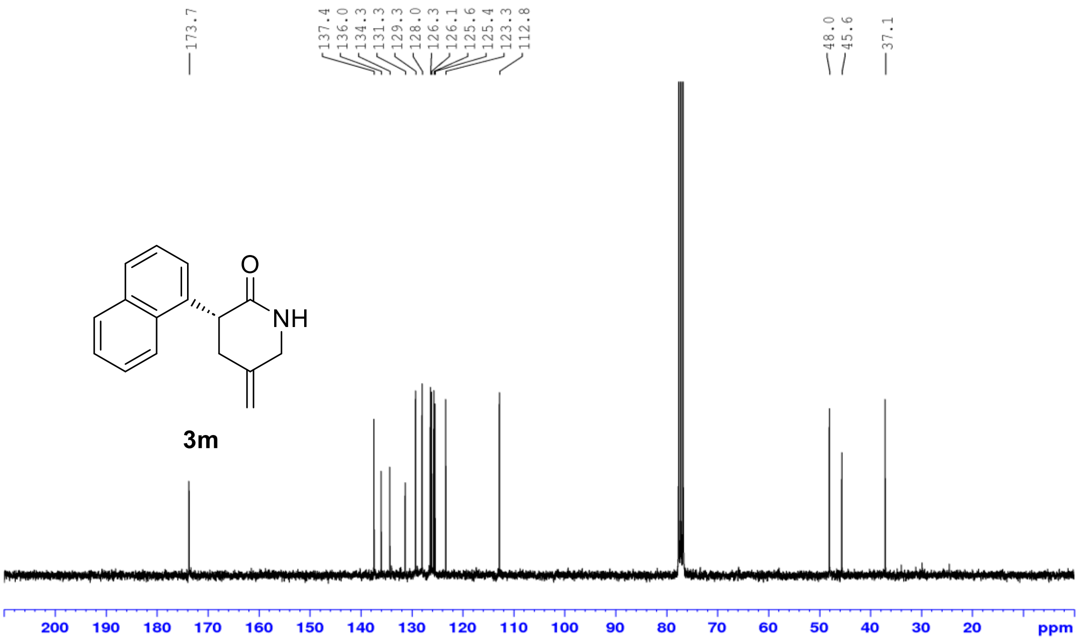

<u>**NMR spectra** of compound **3n**</u>

^{1}H NMR (300 MHz, $CDCl_3$, 298 K)

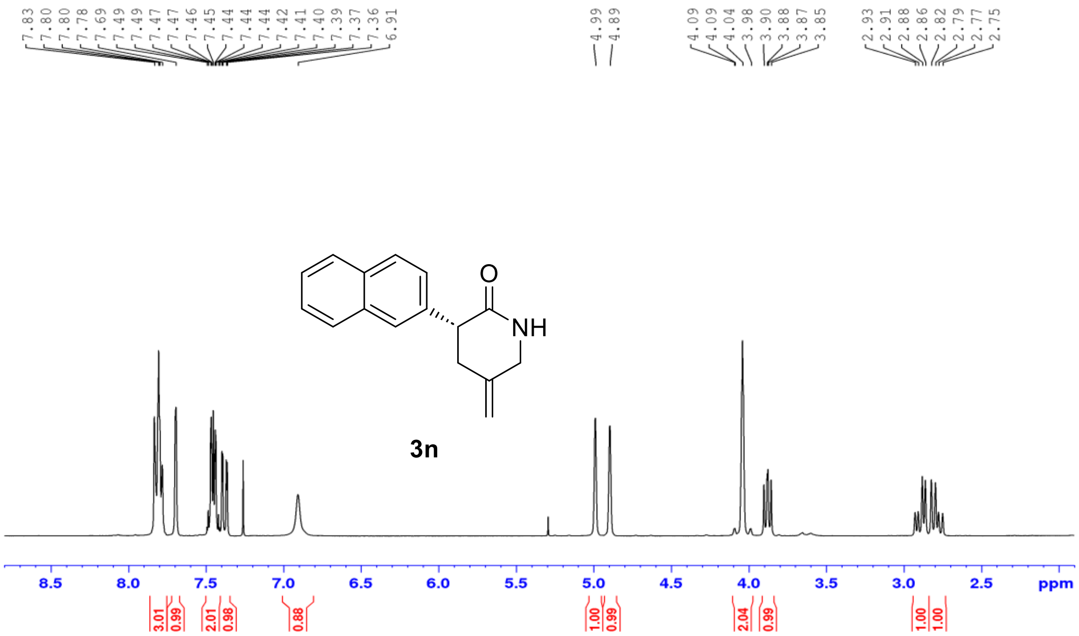

^{13}C NMR (75 MHz, $CDCl_3$, 298 K)

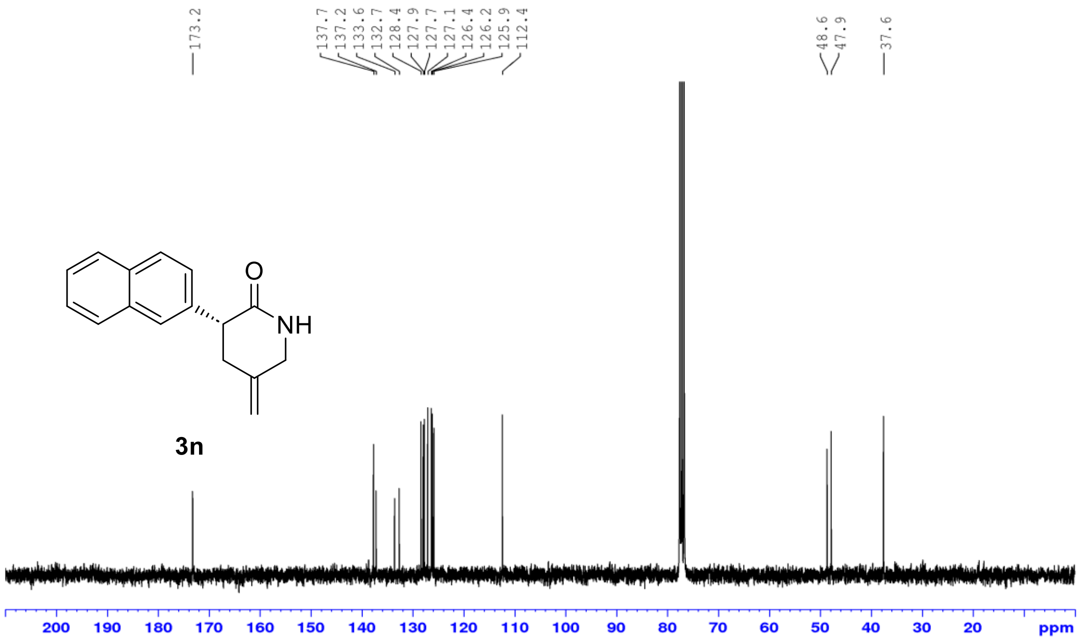

NMR spectra of compound **3o**

^{1}H NMR (300 MHz, $CDCl_3$, 298 K)

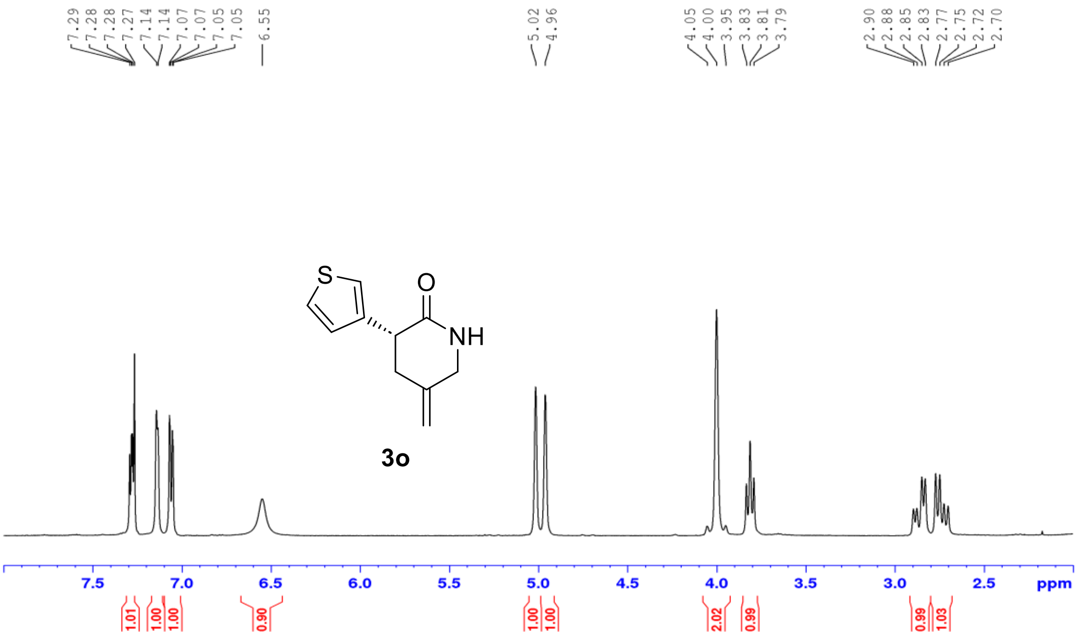

^{13}C NMR (75 MHz, $CDCl_3$, 298 K)

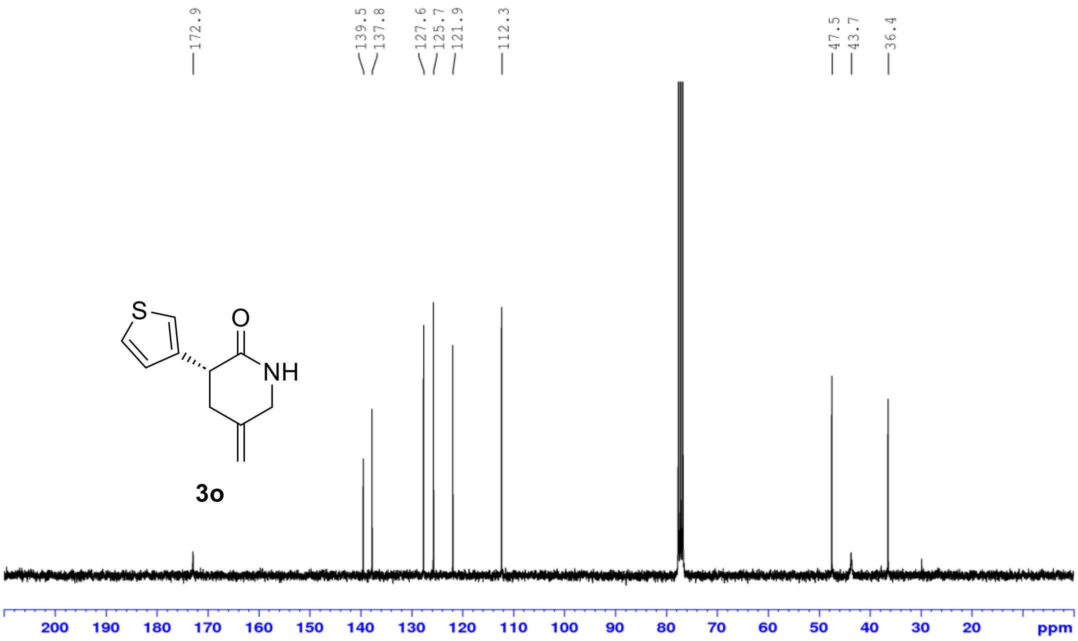

NMR spectra of compound 3p

^{1}H NMR (300 MHz, $CDCl_3$, 298 K)

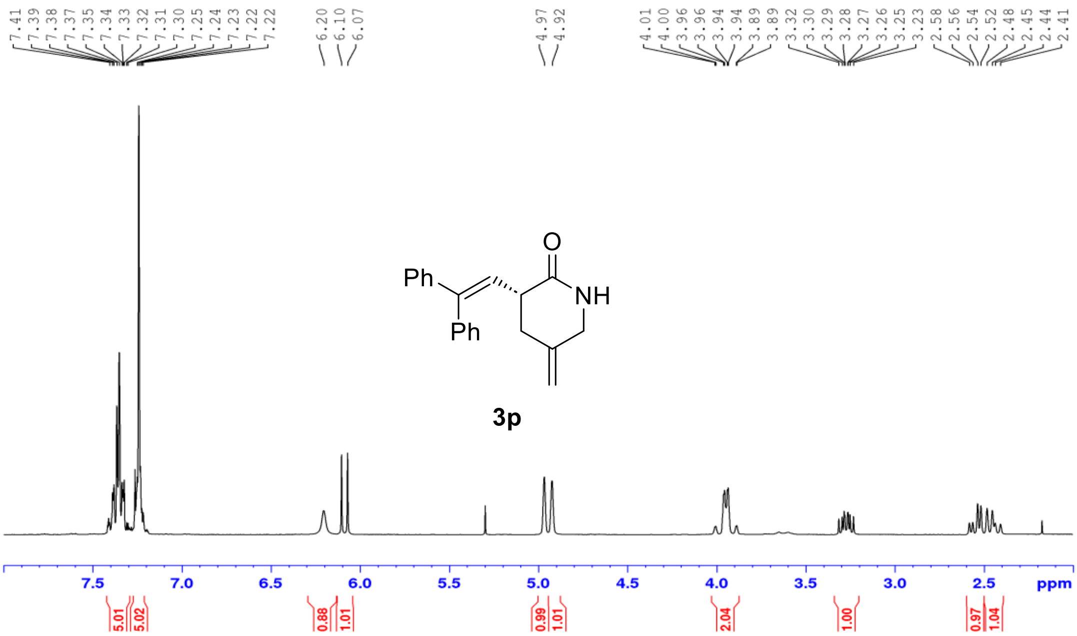

^{13}C NMR (75 MHz, $CDCl_3$, 298 K)

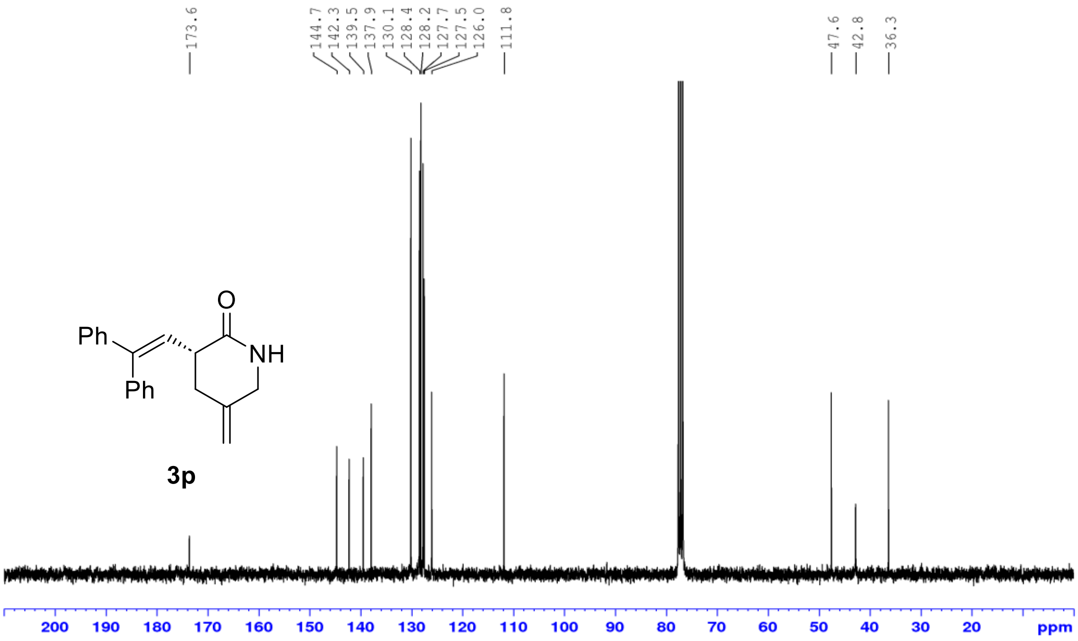

NMR spectra of compound 4a

^{1}H NMR (300 MHz, $CDCl_3$, 298 K)

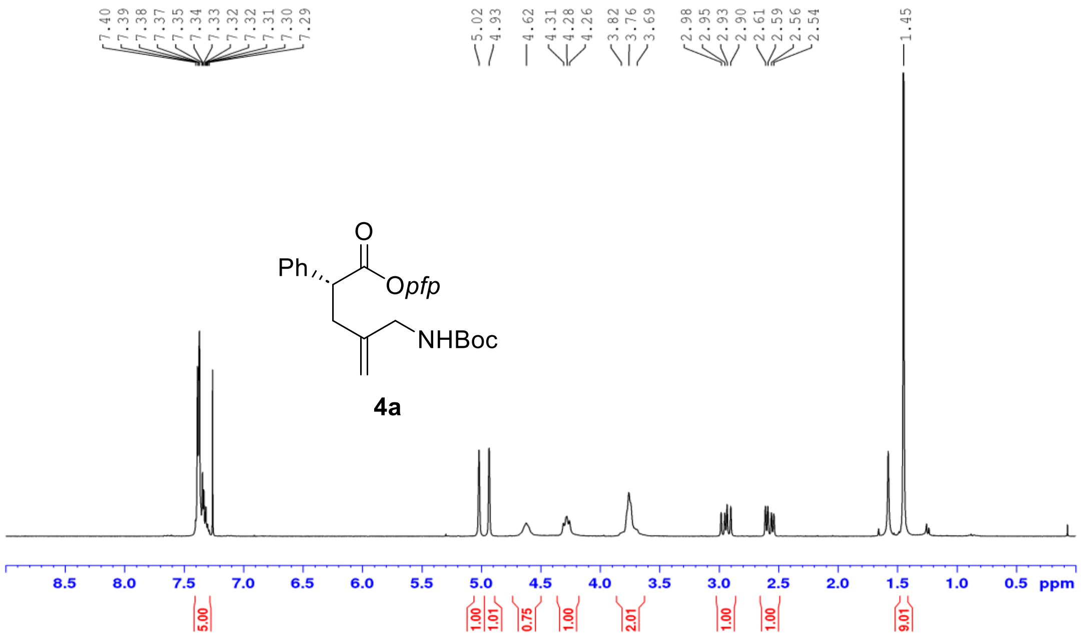

^{13}C NMR (75 MHz, $CDCl_3$, 298 K)

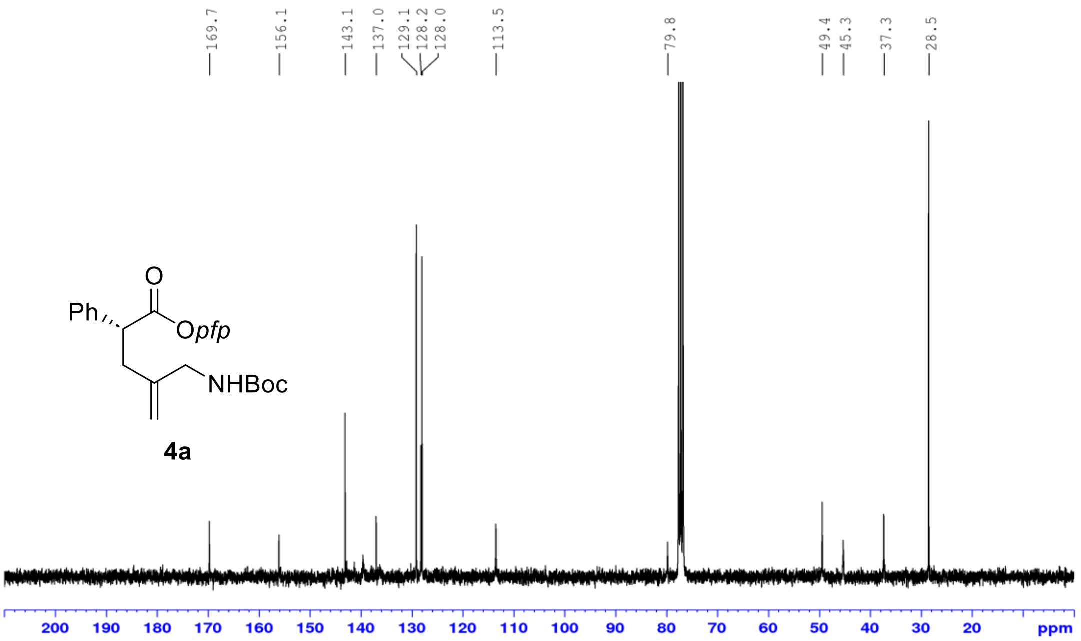

^{19}**F NMR** (282 MHz, CDCl$_3$, 298 K)

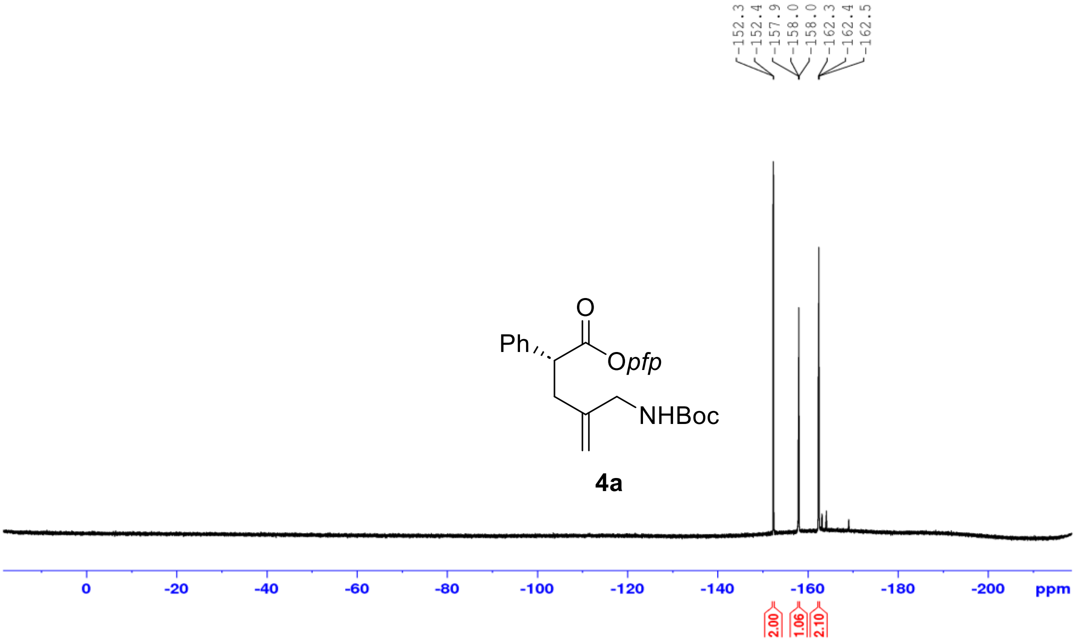

NMR spectra of compound **6a**

^{1}H NMR (300 MHz, $CDCl_3$, 298 K)

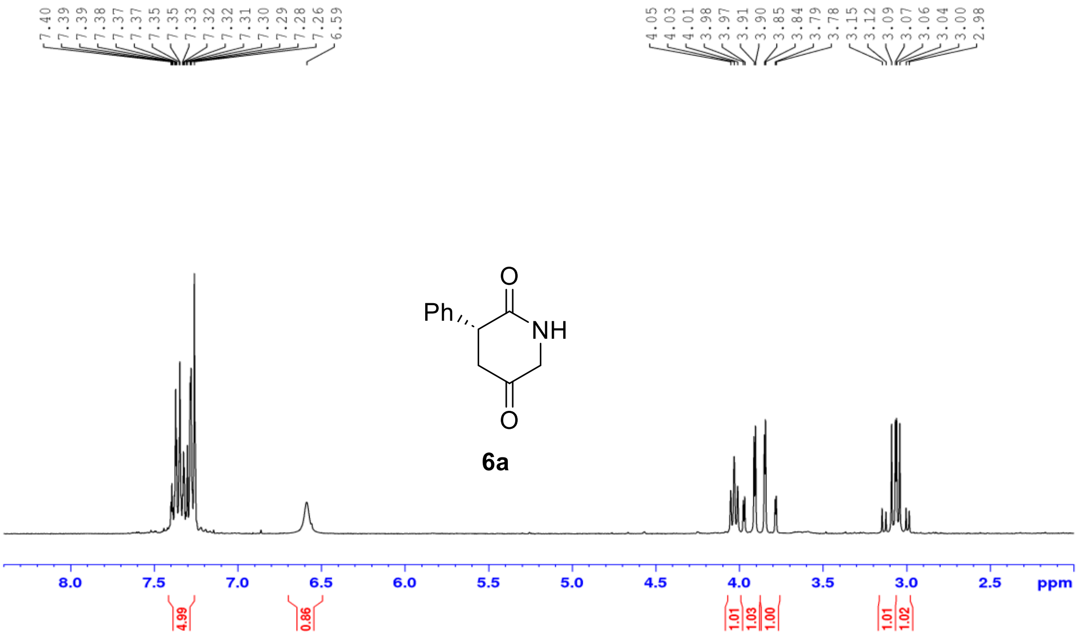

^{13}C NMR (75 MHz, $CDCl_3$, 298 K)

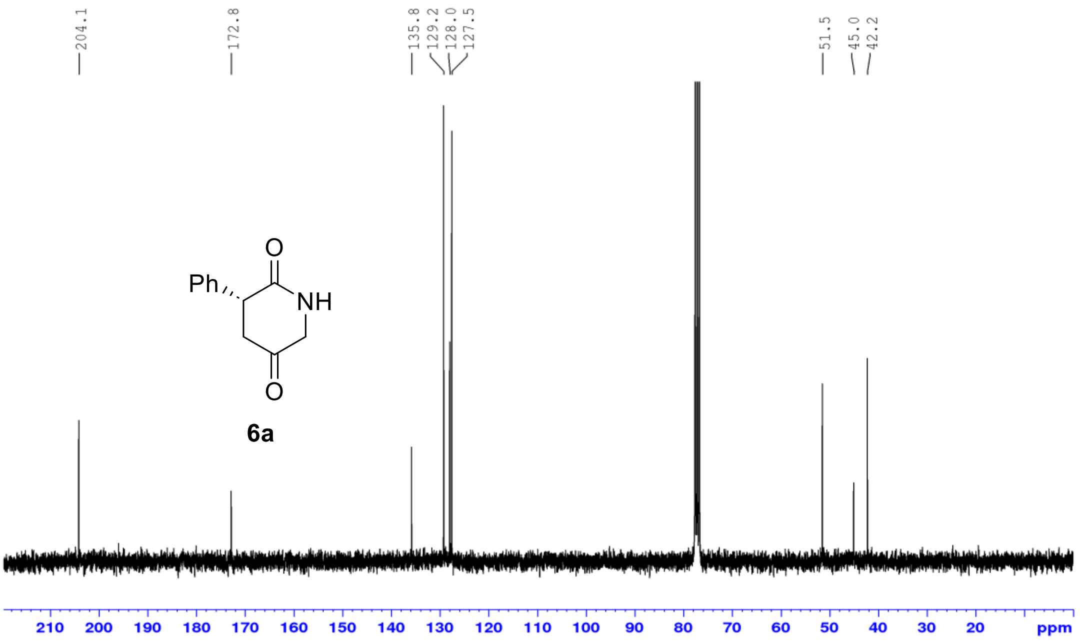

NMR spectra of compound **7a**

^{1}H NMR (300 MHz, DMSO-d_6, 298 K)

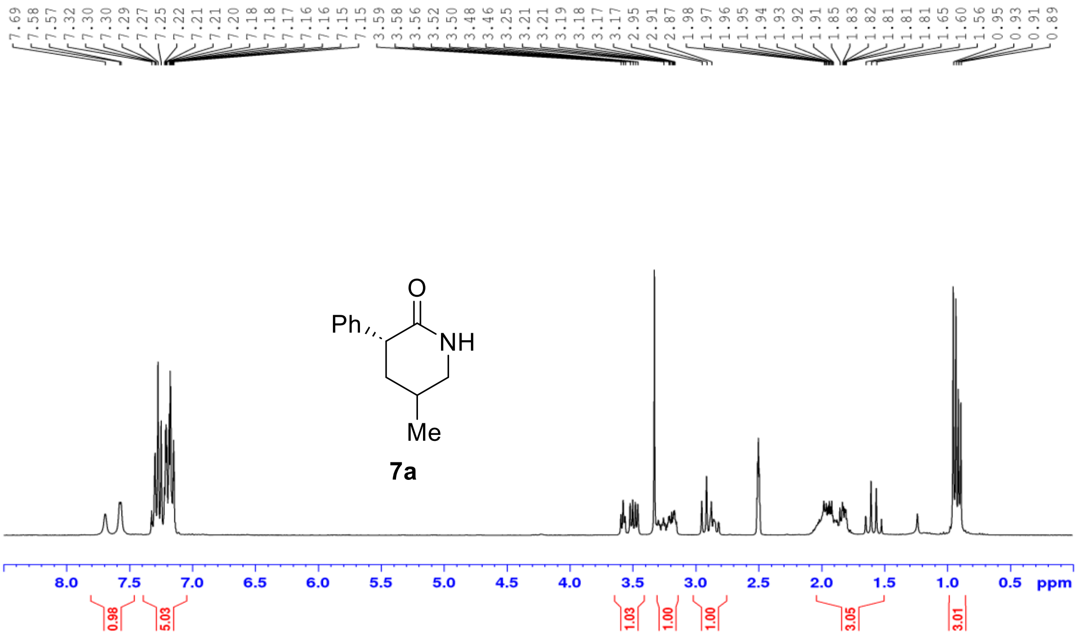

^{13}C NMR (75 MHz, $CDCl_3$, 298 K)

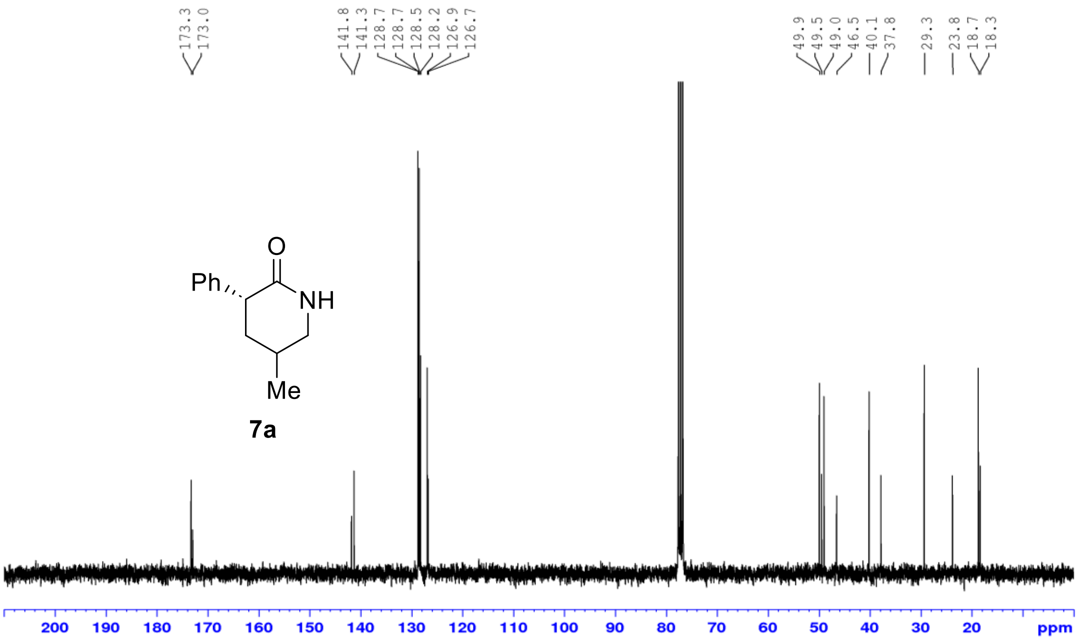

<u>**NMR spectra** of compound **8a**</u>

^{1}H NMR (300 MHz, $CDCl_3$, 298 K)

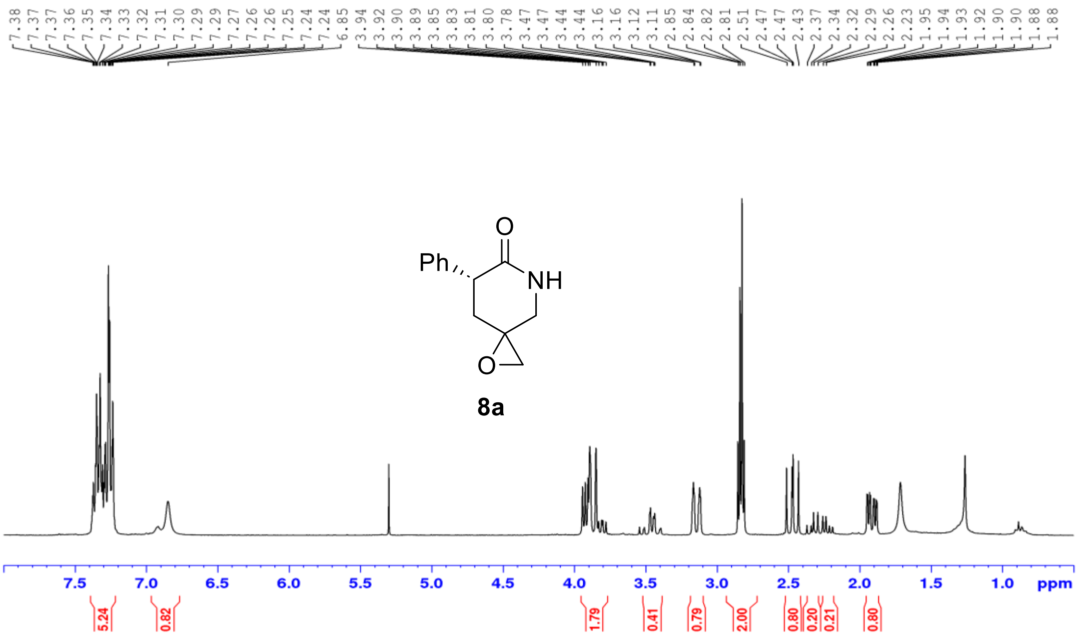

^{13}C NMR (75 MHz, $CDCl_3$, 298 K)

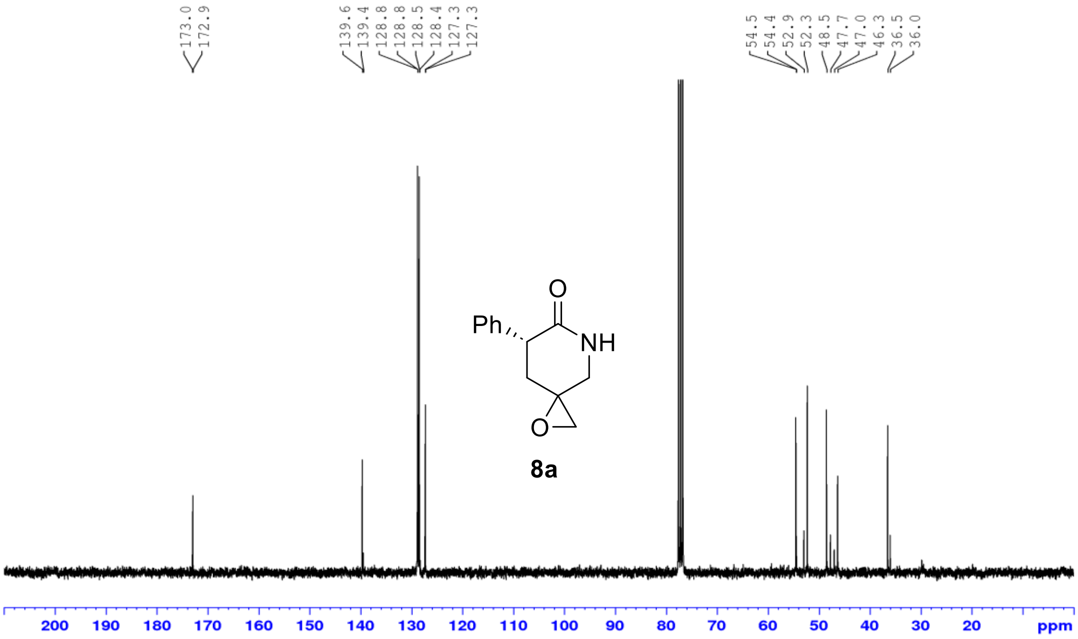

7. Copies of HPLC Chromatograms

<u>HPLC traces of compound 3a</u>

YMC Chiral ART Cellulose-SB, eluent: *n*-hexane:*i*-PrOH = 4/1, 1.0 mL·min^{-1}, 10 °C, λ = 210 nm

Chromatogram

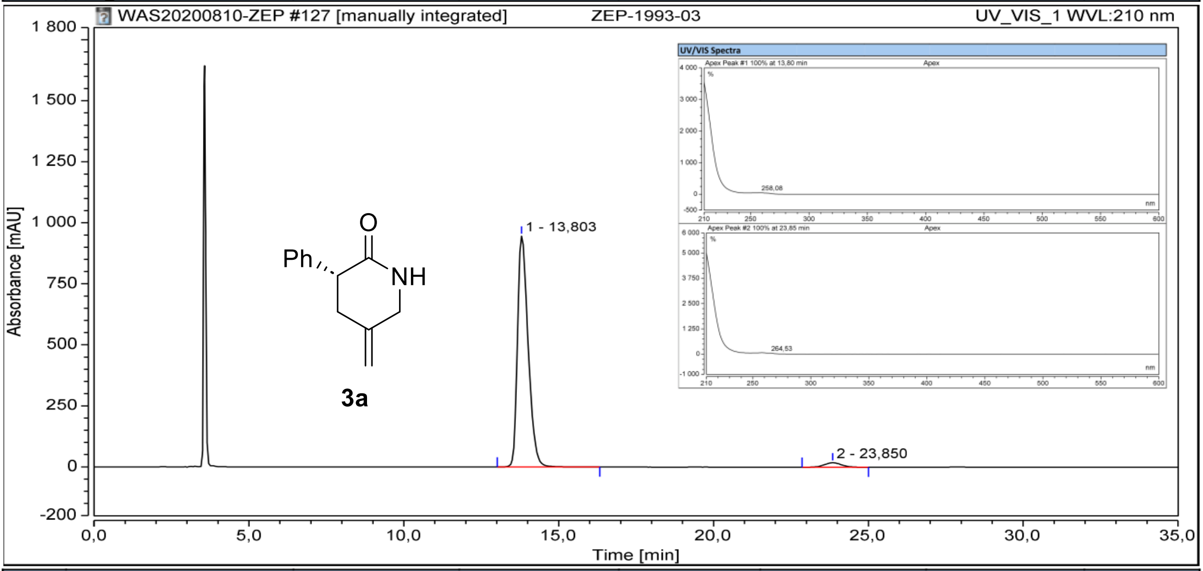

Integration Results

No.	Peak Name	Retention Time min	Area mAU*min	Height mAU	Relative Area %	Relative Height %	Amount
n.a.	Peak 1	n.a.	n.a.	n.a.	n.a.	n.a.	n.a.
1		13,803	378,243	946,541	97,04	98,11	n.a.
2		23,850	11,552	18,255	2,96	1,89	n.a.
**Total:**			**389,795**	**964,796**	**100,00**	**100,00**	

Chromatogram

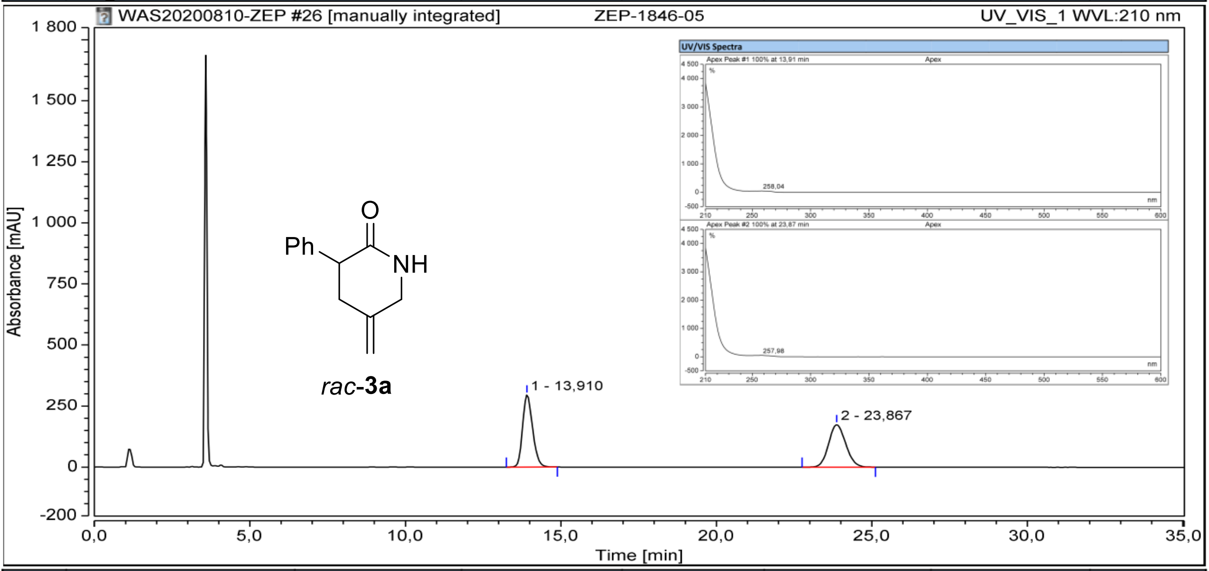

Integration Results

No.	Peak Name	Retention Time min	Area mAU*min	Height mAU	Relative Area %	Relative Height %	Amount
n.a.	Peak 1	n.a.	n.a.	n.a.	n.a.	n.a.	n.a.
1		13,910	110,921	295,904	49,86	62,95	n.a.
2		23,867	111,537	174,158	50,14	37,05	n.a.
**Total:**			**222,458**	**470,062**	**100,00**	**100,00**	

HPLC traces of compound **3b**

YMC Chiral ART Cellulose-SB, eluent: *n*-hexane:*i*-PrOH = 4/1, 1.0 mL·min^{-1}, 10 °C, λ = 210 nm

Chromatogram

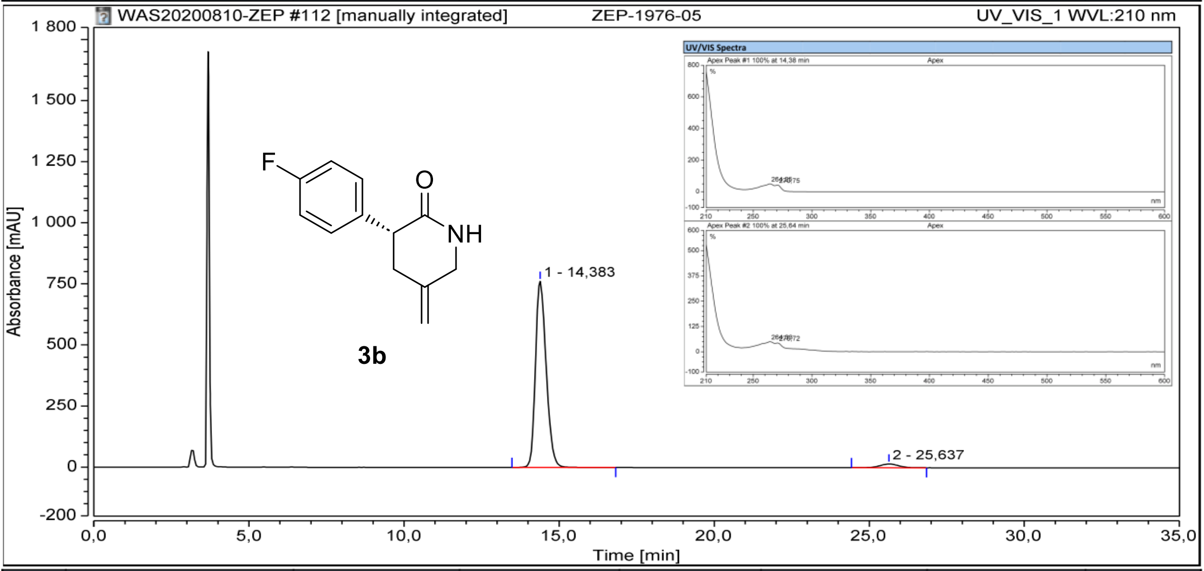

Integration Results

No.	Peak Name	Retention Time min	Area mAU*min	Height mAU	Relative Area %	Relative Height %	Amount
n.a.	Peak 1	n.a.	n.a.	n.a.	n.a.	n.a.	n.a.
1		14,383	307,261	761,997	96,54	98,02	n.a.
2		25,637	11,000	15,392	3,46	1,98	n.a.
**Total:**			**318,261**	**777,389**	**100,00**	**100,00**	

Chromatogram

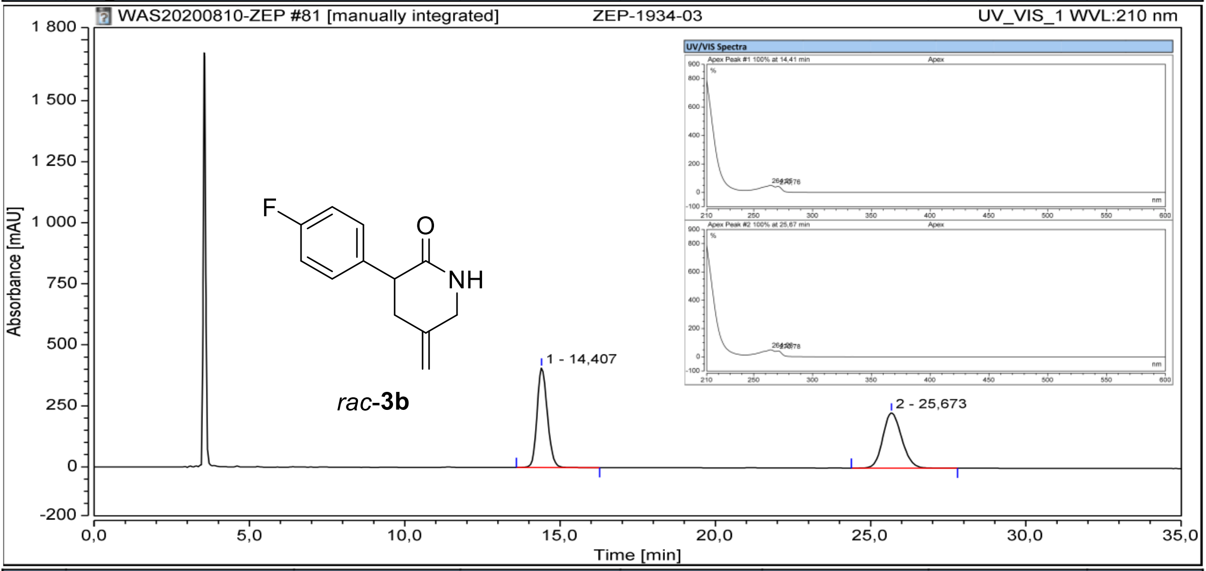

Integration Results

No.	Peak Name	Retention Time min	Area mAU*min	Height mAU	Relative Area %	Relative Height %	Amount
n.a.	Peak 1	n.a.	n.a.	n.a.	n.a.	n.a.	n.a.
1		14,407	158,388	407,967	49,80	64,31	n.a.
2		25,673	159,671	226,415	50,20	35,69	n.a.
**Total:**			**318,059**	**634,382**	**100,00**	**100,00**	

HPLC traces of compound **3c**

YMC Chiral ART Cellulose-SB, eluent: *n*-hexane:*i*-PrOH = 4/1, 1.0 mL·min^{-1}, 10 °C, λ = 210 nm

Chromatogram

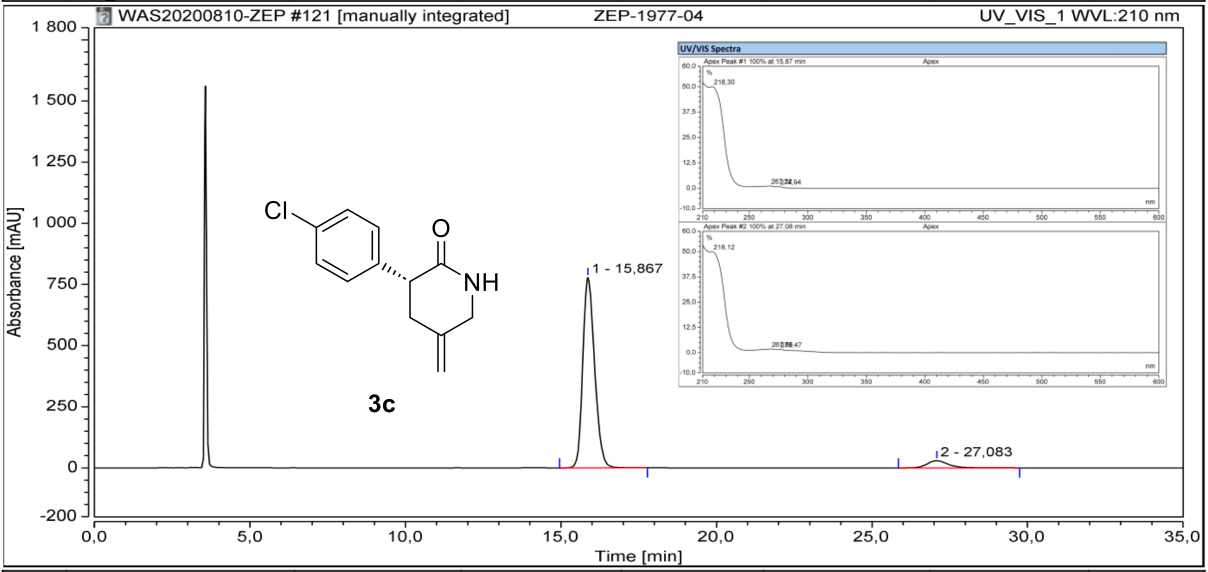

Integration Results

No.	Peak Name	Retention Time min	Area mAU*min	Height mAU	Relative Area %	Relative Height %	Amount
n.a.	Peak 1	n.a.	n.a.	n.a.	n.a.	n.a.	n.a.
1		15,867	343,818	777,976	93,48	96,36	n.a.
2		27,083	23,979	29,354	6,52	3,64	n.a.
**Total:**			**367,797**	**807,330**	**100,00**	**100,00**	

Chromatogram

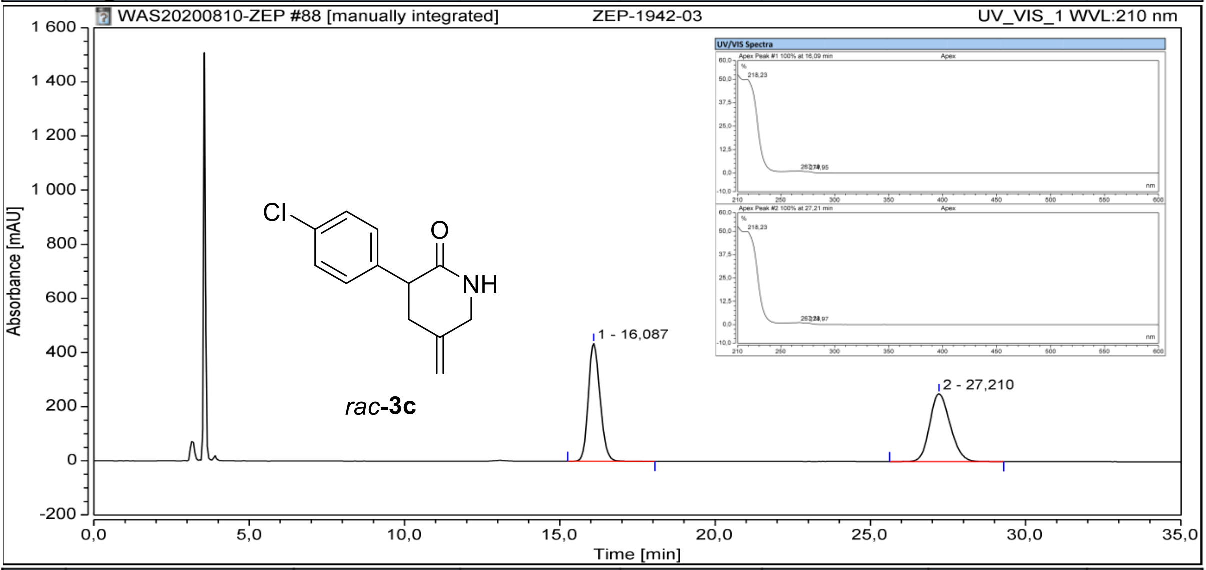

Integration Results

No.	Peak Name	Retention Time min	Area mAU*min	Height mAU	Relative Area %	Relative Height %	Amount
n.a.	Peak 1	n.a.	n.a.	n.a.	n.a.	n.a.	n.a.
1		16,087	191,746	436,283	49,93	63,50	n.a.
2		27,210	192,315	250,774	50,07	36,50	n.a.
**Total:**			**384,061**	**687,057**	**100,00**	**100,00**	

HPLC traces of compound **3d**

YMC Chiral ART Cellulose-SB, eluent: *n*-hexane:*i*-PrOH = 4/1, 1.0 mL·min^{-1}, 10 °C, λ = 210 nm

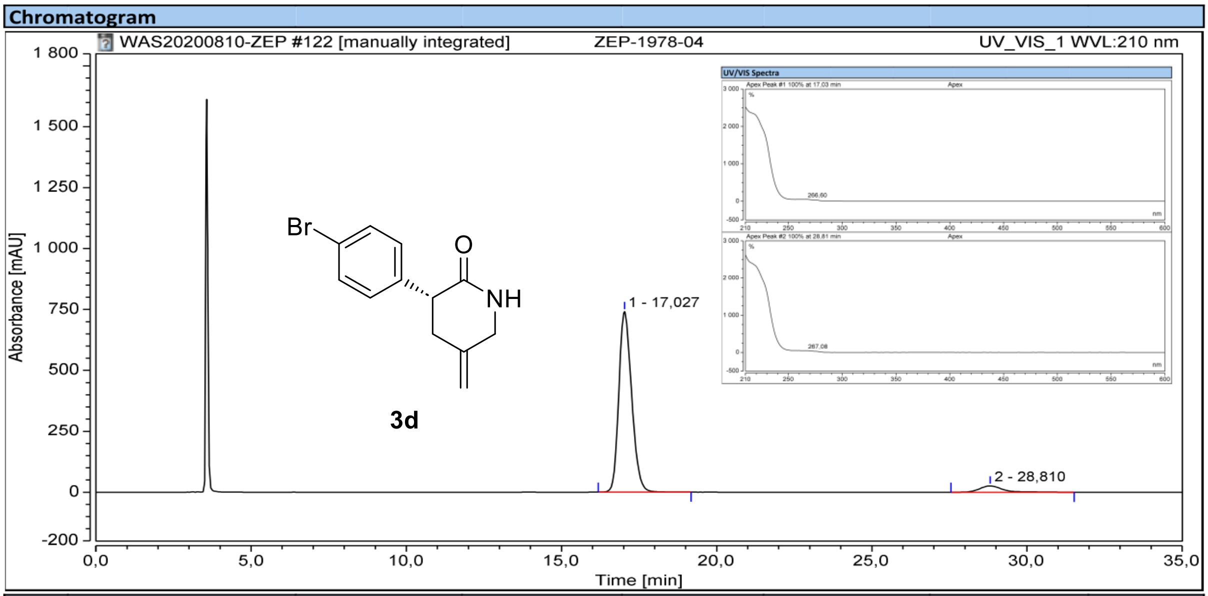

Integration Results

No.	Peak Name	Retention Time min	Area mAU*min	Height mAU	Relative Area %	Relative Height %	Amount
n.a.	Peak 1	n.a.	n.a.	n.a.	n.a.	n.a.	n.a.
1		17,027	356,238	741,684	93,95	96,61	n.a.
2		28,810	22,928	26,049	6,05	3,39	n.a.
**Total:**			**379,166**	**767,733**	**100,00**	**100,00**	

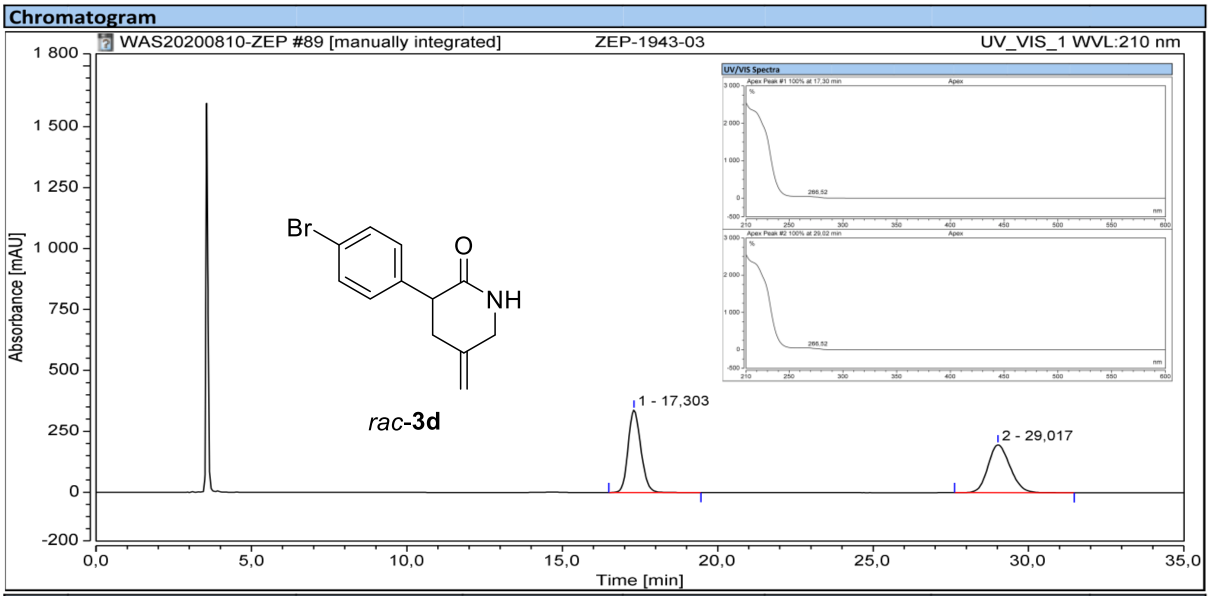

Integration Results

No.	Peak Name	Retention Time min	Area mAU*min	Height mAU	Relative Area %	Relative Height %	Amount
n.a.	Peak 1	n.a.	n.a.	n.a.	n.a.	n.a.	n.a.
1		17,303	161,719	338,504	49,97	63,28	n.a.
2		29,017	161,942	196,401	50,03	36,72	n.a.
**Total:**			**323,661**	**534,905**	**100,00**	**100,00**	

<u>HPLC traces</u> <u>of compound **3e**</u>

CHIRALPAK OD-H, eluent: *n*-hexane:*i*-PrOH = 4/1, 1.0 mL·min^{-1}, 10 °C, λ = 210 nm

Chromatogram

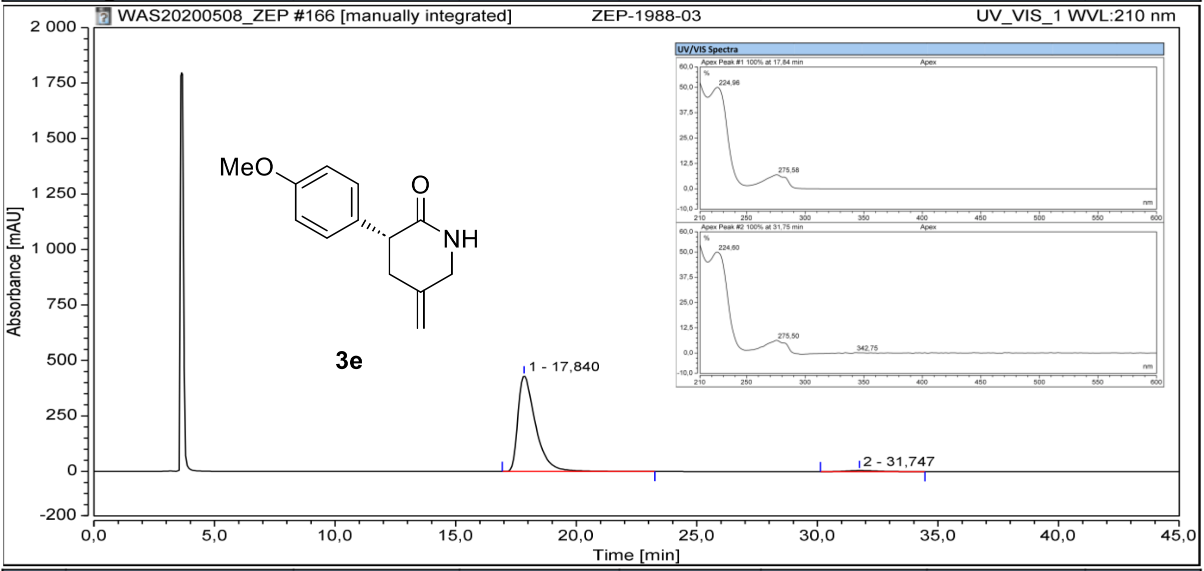

Integration Results

No.	Peak Name	Retention Time<br>min	Area<br>mAU*min	Height<br>mAU	Relative Area<br>%	Relative Height<br>%	Amount
n.a.	Peak 1	n.a.	n.a.	n.a.	n.a.	n.a.	n.a.
1		17,840	366,063	430,838	98,01	98,85	n.a.
2		31,747	7,414	5,019	1,99	1,15	n.a.
**Total:**			**373,477**	**435,858**	**100,00**	**100,00**	

Chromatogram

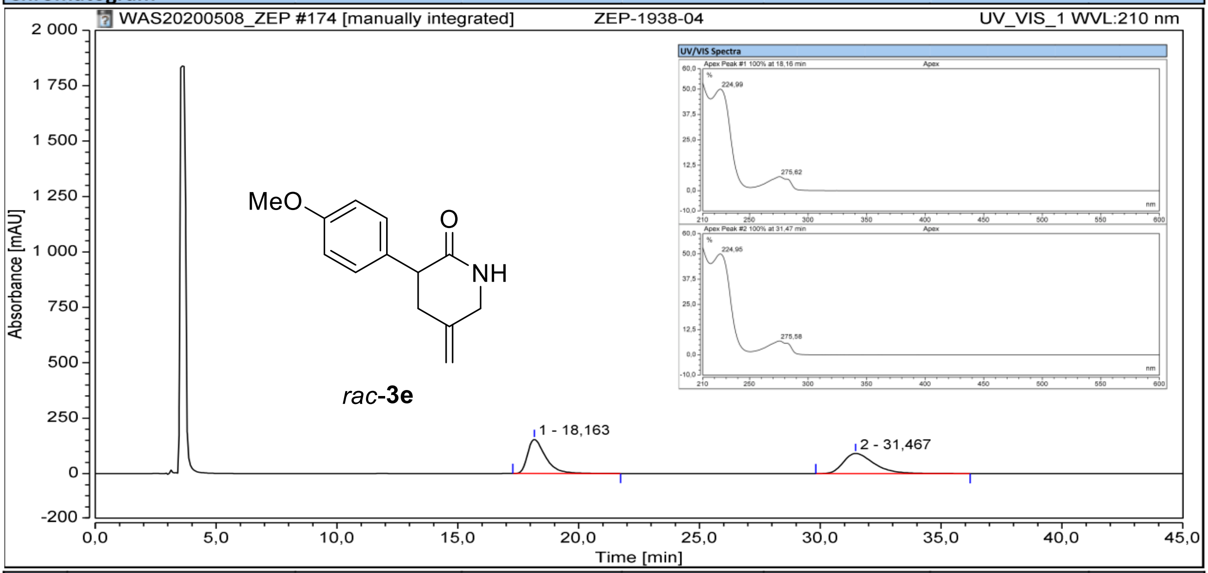

Integration Results

No.	Peak Name	Retention Time<br>min	Area<br>mAU*min	Height<br>mAU	Relative Area<br>%	Relative Height<br>%	Amount
n.a.	Peak 1	n.a.	n.a.	n.a.	n.a.	n.a.	n.a.
1		18,163	133,144	153,777	49,76	62,81	n.a.
2		31,467	134,403	91,059	50,24	37,19	n.a.
**Total:**			**267,547**	**244,835**	**100,00**	**100,00**	

HPLC traces of compound **3f**

CHIRALPAK OD-H, eluent: *n*-hexane:*i*-PrOH = 4/1, 1.0 mL·min^{-1}, 10 °C, λ = 210 nm

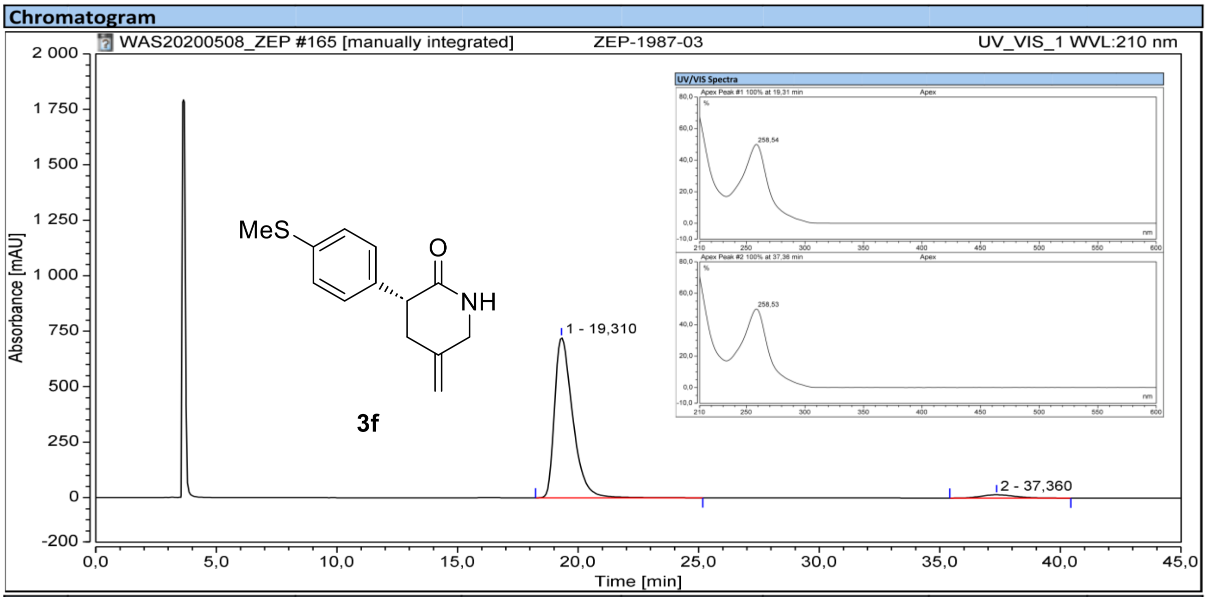

Integration Results							
No.	Peak Name	Retention Time min	Area mAU*min	Height mAU	Relative Area %	Relative Height %	Amount
n.a.	Peak 1	n.a.	n.a.	n.a.	n.a.	n.a.	n.a.
1		19,310	631,116	721,128	96,35	98,02	n.a.
2		37,360	23,876	14,589	3,65	1,98	n.a.
**Total:**			**654,992**	**735,717**	**100,00**	**100,00**	

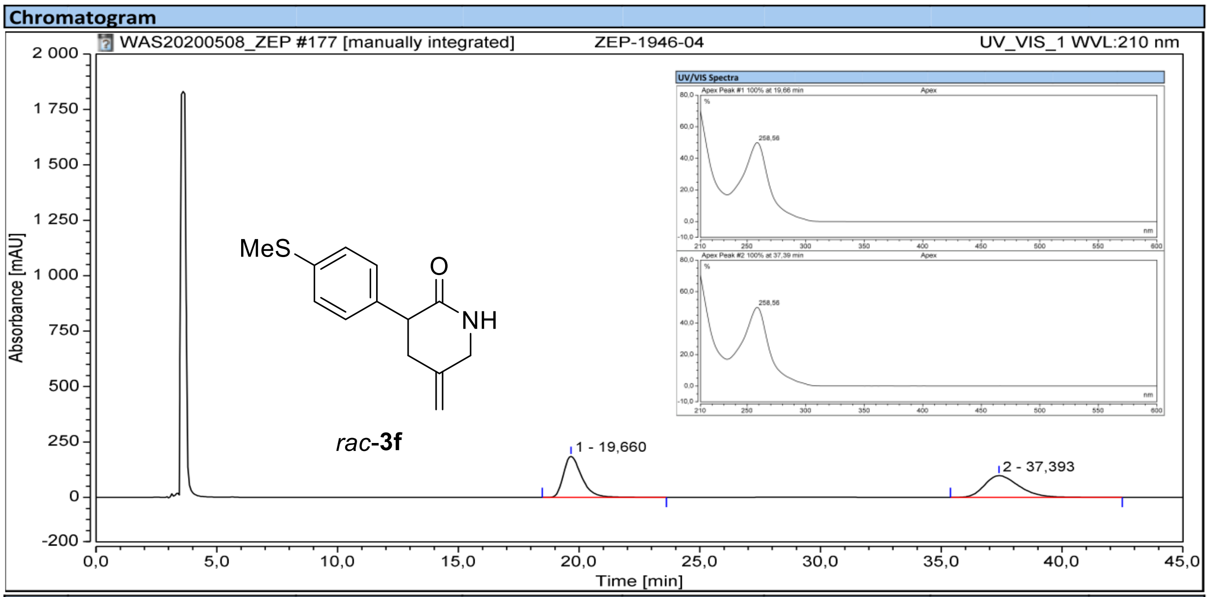

Integration Results							
No.	Peak Name	Retention Time min	Area mAU*min	Height mAU	Relative Area %	Relative Height %	Amount
n.a.	Peak 1	n.a.	n.a.	n.a.	n.a.	n.a.	n.a.
1		19,660	163,587	185,592	49,82	65,36	n.a.
2		37,393	164,785	98,368	50,18	34,64	n.a.
**Total:**			**328,372**	**283,960**	**100,00**	**100,00**	

HPLC traces of compound **3g**

YMC Chiral ART Cellulose-SB, eluent: *n*-hexane:*i*-PrOH = 4/1, 1.0 mL·min^{-1}, 10 °C, λ = 210 nm

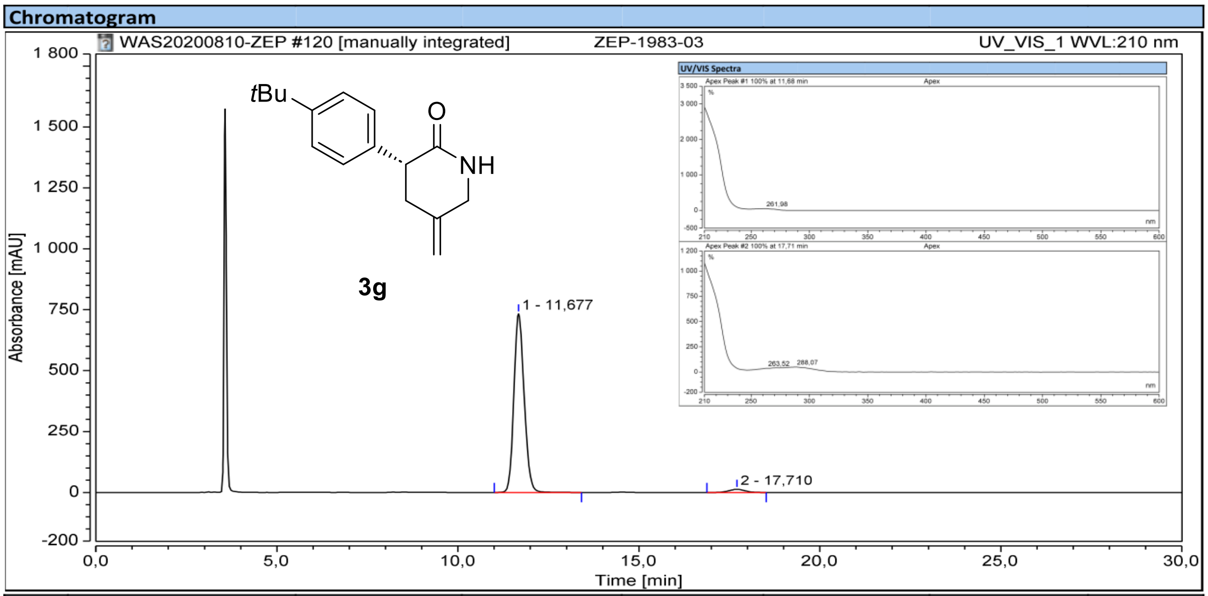

Integration Results

No.	Peak Name	Retention Time min	Area mAU*min	Height mAU	Relative Area %	Relative Height %	Amount
n.a.	Peak 1	n.a.	n.a.	n.a.	n.a.	n.a.	n.a.
1		11,677	242,070	733,137	97,31	98,26	n.a.
2		17,710	6,700	13,013	2,69	1,74	n.a.
**Total:**			**248,770**	**746,150**	**100,00**	**100,00**	

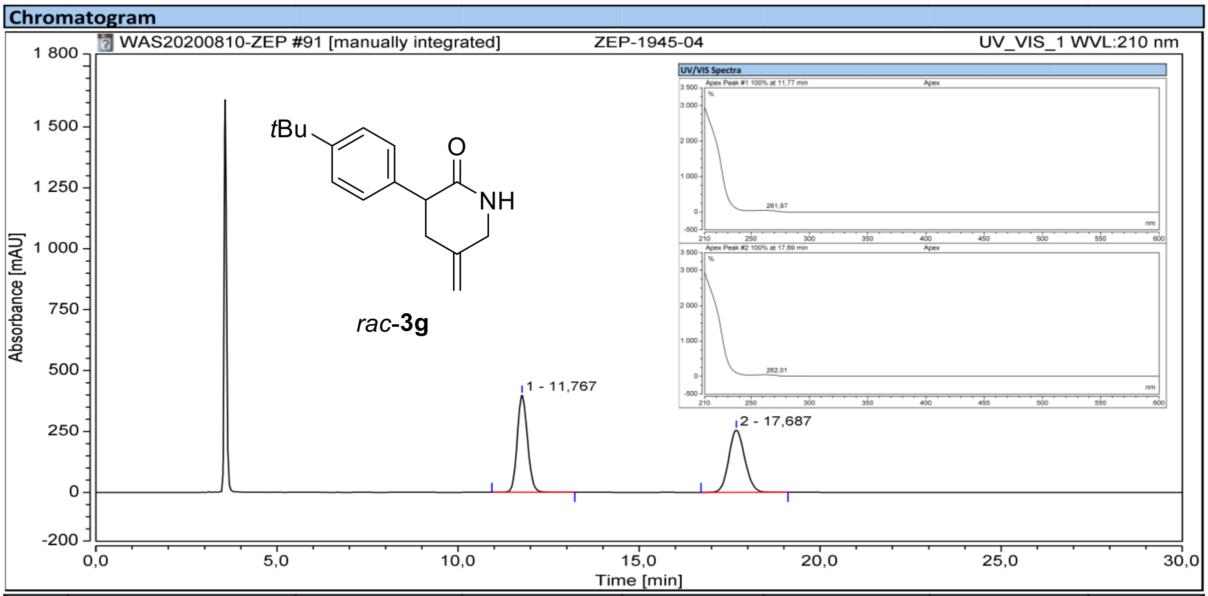

Integration Results

No.	Peak Name	Retention Time min	Area mAU*min	Height mAU	Relative Area %	Relative Height %	Amount
n.a.	Peak 1	n.a.	n.a.	n.a.	n.a.	n.a.	n.a.
1		11,767	131,173	399,213	49,93	60,93	n.a.
2		17,687	131,558	255,977	50,07	39,07	n.a.
**Total:**			**262,731**	**655,190**	**100,00**	**100,00**	

<u>**HPLC traces** of compound **3h**</u>

YMC Chiral ART Cellulose-SB, eluent: *n*-hexane:*i*-PrOH = 4/1, 1.0 mL·min^{-1}, 10 °C, λ = 210 nm

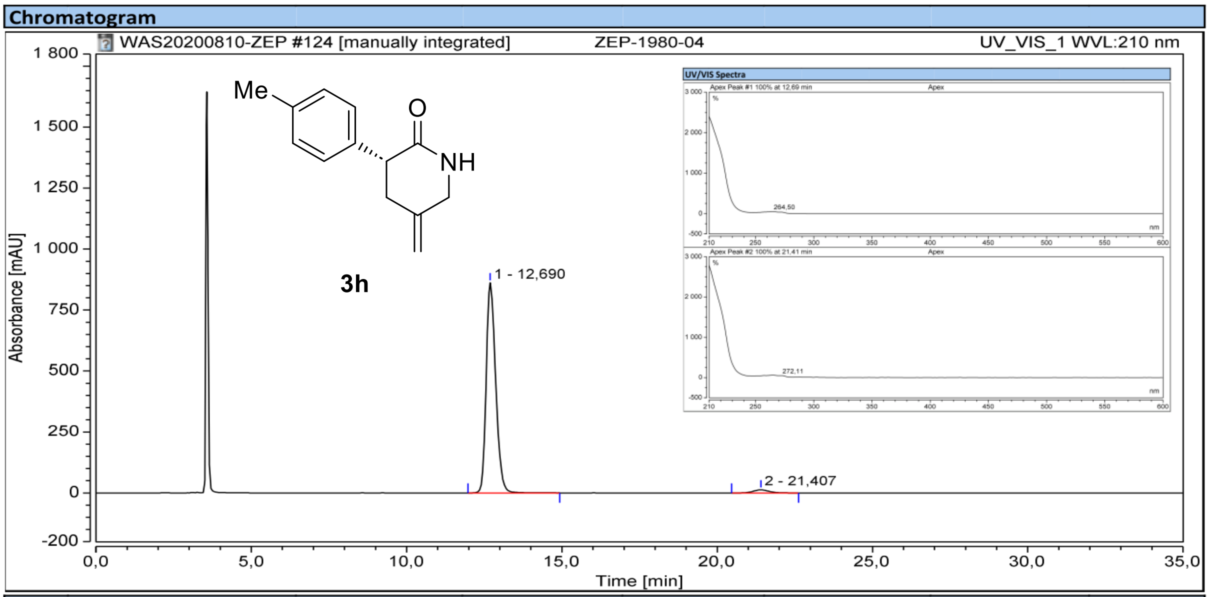

Integration Results

No.	Peak Name	Retention Time min	Area mAU*min	Height mAU	Relative Area %	Relative Height %	Amount
n.a.	Peak 1	n.a.	n.a.	n.a.	n.a.	n.a.	n.a.
1		12,690	306,247	861,627	97,57	98,54	n.a.
2		21,407	7,615	12,788	2,43	1,46	n.a.
**Total:**			**313,862**	**874,415**	**100,00**	**100,00**	

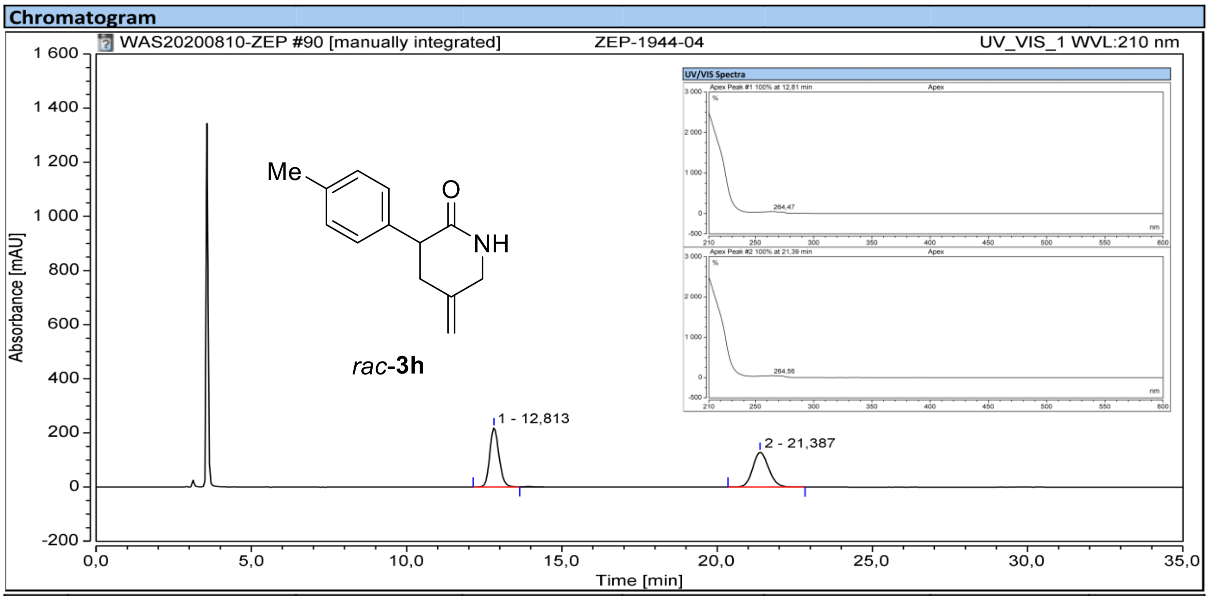

Integration Results

No.	Peak Name	Retention Time min	Area mAU*min	Height mAU	Relative Area %	Relative Height %	Amount
n.a.	Peak 1	n.a.	n.a.	n.a.	n.a.	n.a.	n.a.
1		12,813	75,949	218,835	49,87	62,96	n.a.
2		21,387	76,357	128,754	50,13	37,04	n.a.
**Total:**			**152,306**	**347,589**	**100,00**	**100,00**	

HPLC traces of compound **3i**

YMC Chiral ART Cellulose-SB, eluent: *n*-hexane:*i*-PrOH = 4/1, 1.0 mL·min^{-1}, 10 °C, λ = 210 nm

Chromatogram

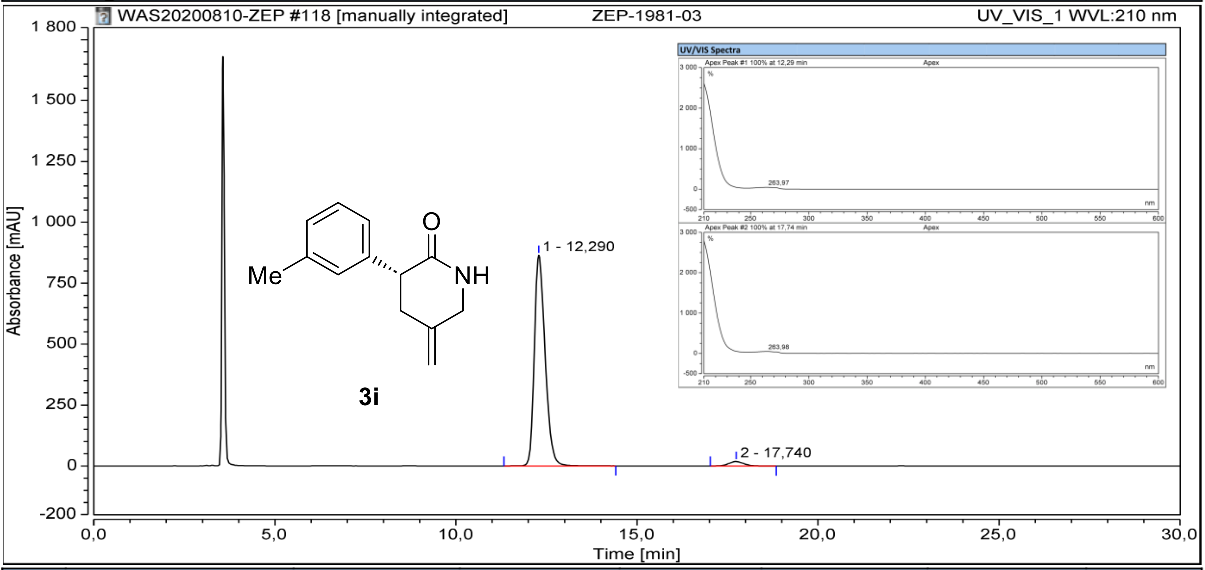

Integration Results

No.	Peak Name	Retention Time min	Area mAU*min	Height mAU	Relative Area %	Relative Height %	Amount
n.a.	Peak 1	n.a.	n.a.	n.a.	n.a.	n.a.	n.a.
1		12,290	300,175	864,546	97,11	97,90	n.a.
2		17,740	8,944	18,518	2,89	2,10	n.a.
**Total:**			**309,119**	**883,065**	**100,00**	**100,00**	

Chromatogram

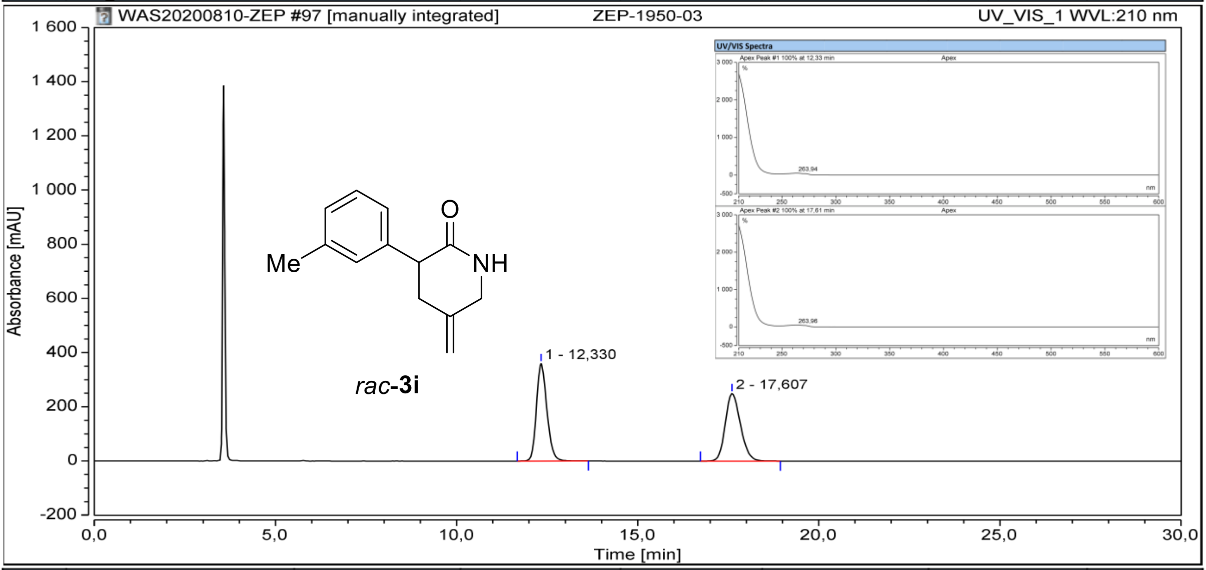

Integration Results

No.	Peak Name	Retention Time min	Area mAU*min	Height mAU	Relative Area %	Relative Height %	Amount
n.a.	Peak 1	n.a.	n.a.	n.a.	n.a.	n.a.	n.a.
1		12,330	120,994	359,539	49,92	59,05	n.a.
2		17,607	121,375	249,350	50,08	40,95	n.a.
**Total:**			**242,368**	**608,889**	**100,00**	**100,00**	

HPLC traces of compound **3j**

YMC Chiral ART Cellulose-SB, eluent: *n*-hexane:*i*-PrOH = 4/1, 1.0 mL·min^{-1}, 10 °C, λ = 210 nm

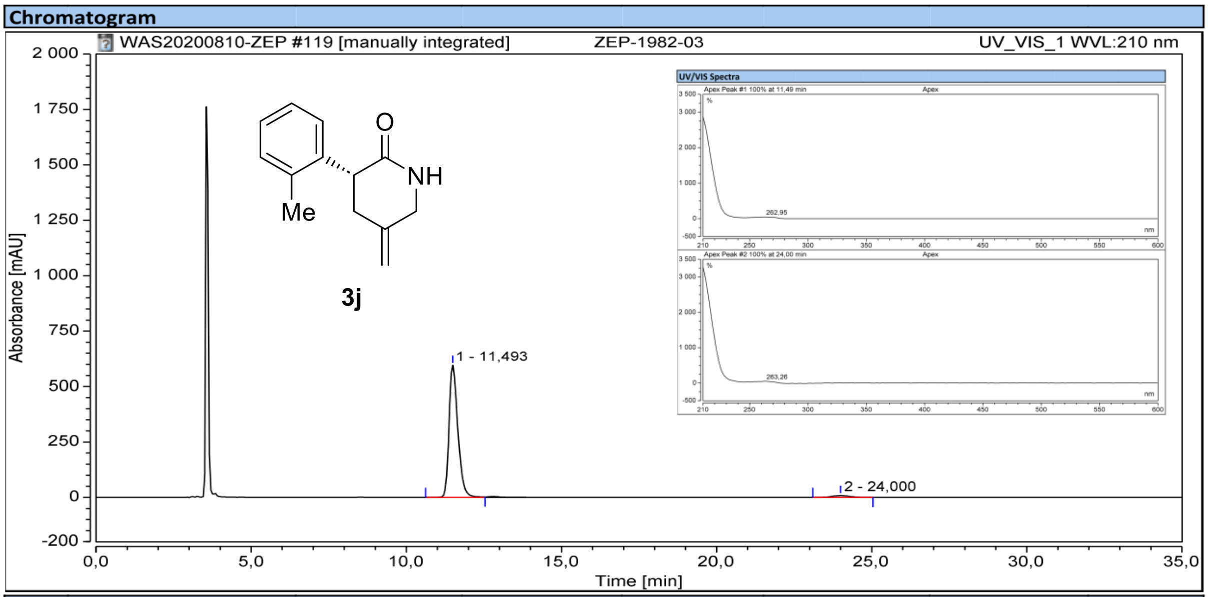

Integration Results

No.	Peak Name	Retention Time<br>min	Area<br>mAU*min	Height<br>mAU	Relative Area<br>%	Relative Height<br>%	Amount
n.a.	Peak 1	n.a.	n.a.	n.a.	n.a.	n.a.	n.a.
1		11,493	197,222	596,287	97,20	98,59	n.a.
2		24,000	5,688	8,507	2,80	1,41	n.a.
**Total:**			**202,911**	**604,795**	**100,00**	**100,00**	

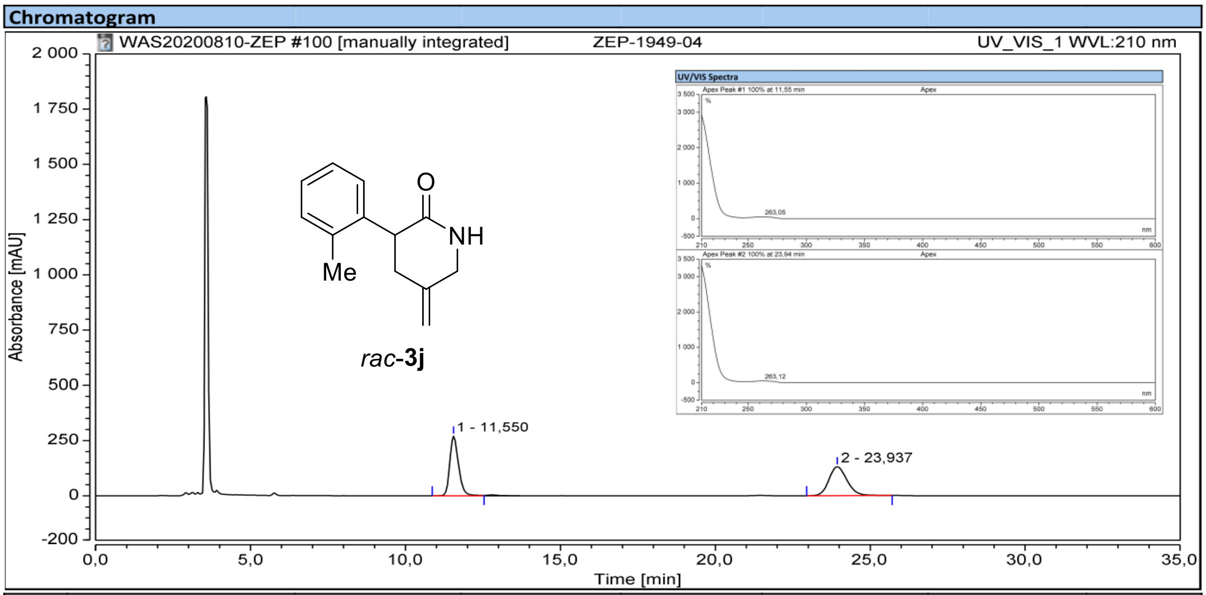

Integration Results

No.	Peak Name	Retention Time<br>min	Area<br>mAU*min	Height<br>mAU	Relative Area<br>%	Relative Height<br>%	Amount
n.a.	Peak 1	n.a.	n.a.	n.a.	n.a.	n.a.	n.a.
1		11,550	89,355	270,464	50,18	67,40	n.a.
2		23,937	88,697	130,844	49,82	32,60	n.a.
**Total:**			**178,052**	**401,307**	**100,00**	**100,00**	

<u>**HPLC traces** of compound **3k**</u>

CHIRALPAK OD-H, eluent: *n*-hexane:*i*-PrOH = 2/1, 1.0 mL·min^{-1}, 10 °C, λ = 210 nm

Chromatogram

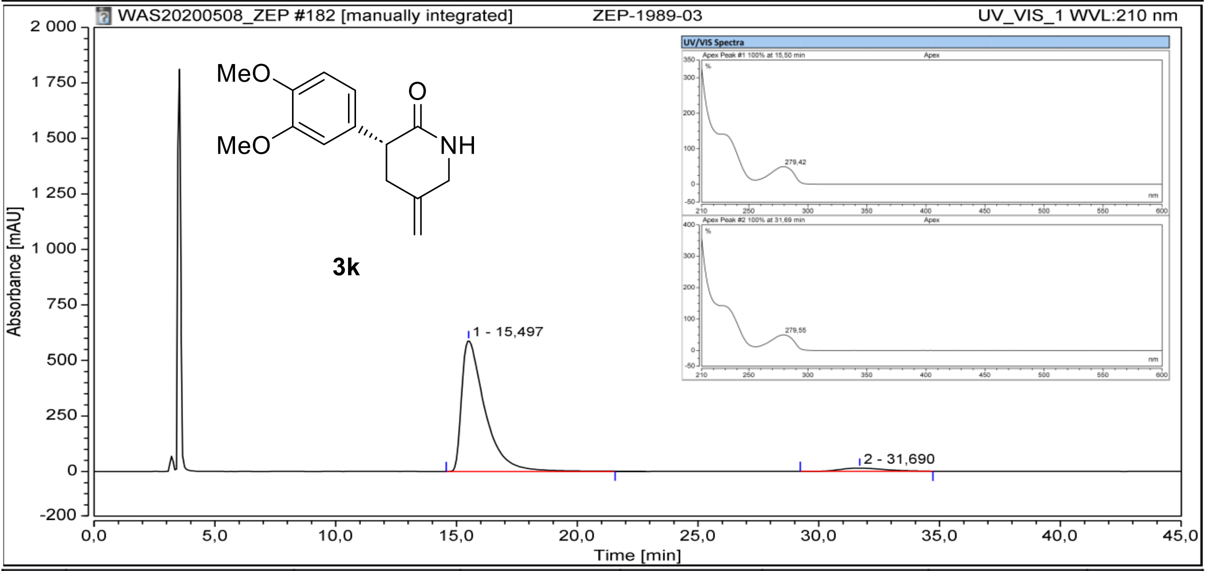

Integration Results

No.	Peak Name	Retention Time min	Area mAU*min	Height mAU	Relative Area %	Relative Height %	Amount
n.a.	Peak 1	n.a.	n.a.	n.a.	n.a.	n.a.	n.a.
1		15,497	660,427	588,713	95,48	97,57	n.a.
2		31,690	31,300	14,665	4,52	2,43	n.a.
**Total:**			**691,727**	**603,378**	**100,00**	**100,00**	

Chromatogram

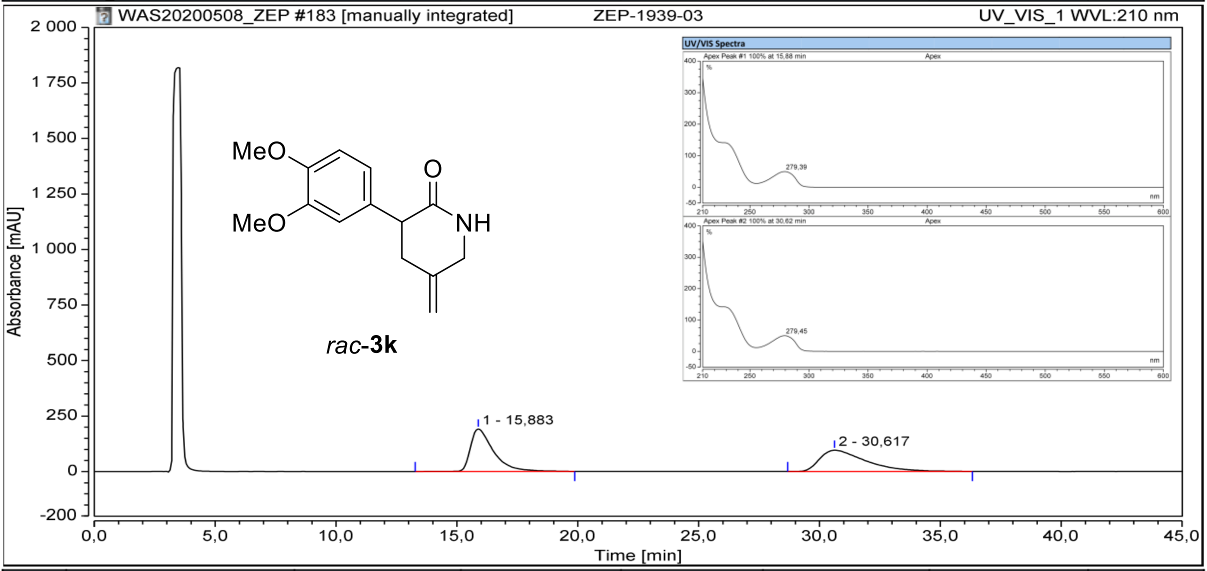

Integration Results

No.	Peak Name	Retention Time min	Area mAU*min	Height mAU	Relative Area %	Relative Height %	Amount
n.a.	Peak 1	n.a.	n.a.	n.a.	n.a.	n.a.	n.a.
1		15,883	213,351	191,350	50,22	66,75	n.a.
2		30,617	211,496	95,300	49,78	33,25	n.a.
**Total:**			**424,847**	**286,650**	**100,00**	**100,00**	

<u>**HPLC traces** of compound **3l**</u>

YMC Chiral ART Cellulose-SB, eluent: *n*-hexane:*i*-PrOH = 4/1, 1.0 mL·min^{-1}, 10 °C, λ = 210 nm

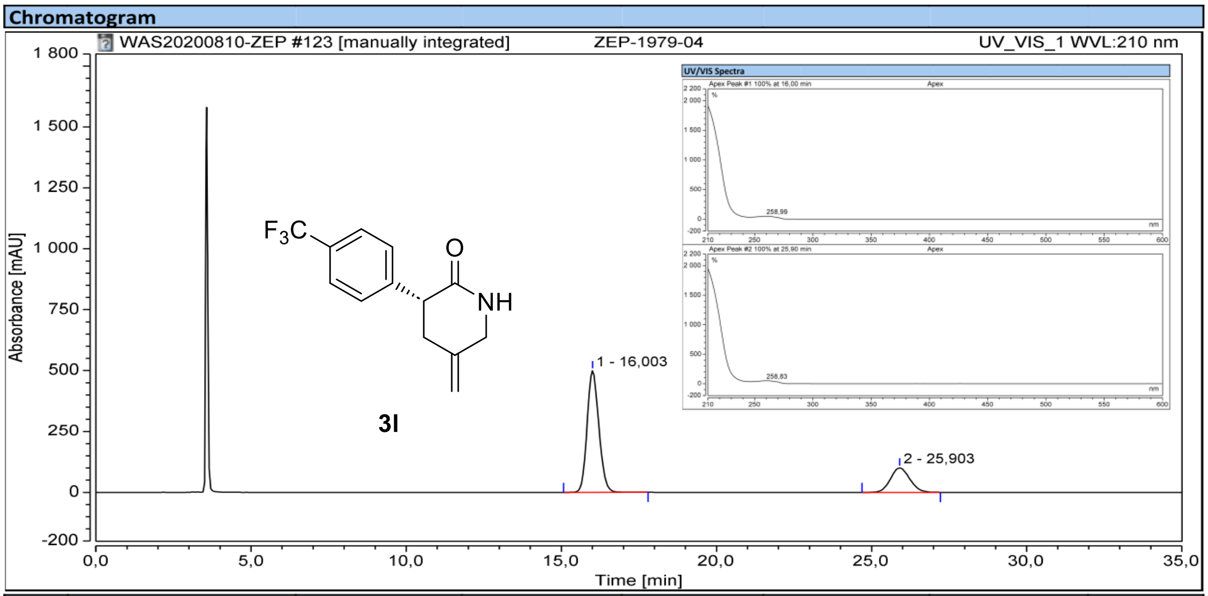

Integration Results							
No.	Peak Name	Retention Time min	Area mAU*min	Height mAU	Relative Area %	Relative Height %	Amount
n.a.	Peak 1	n.a.	n.a.	n.a.	n.a.	n.a.	n.a.
1		16,003	220,287	499,420	75,12	83,25	n.a.
2		25,903	72,962	100,469	24,88	16,75	n.a.
**Total:**			**293,250**	**599,889**	**100,00**	**100,00**	

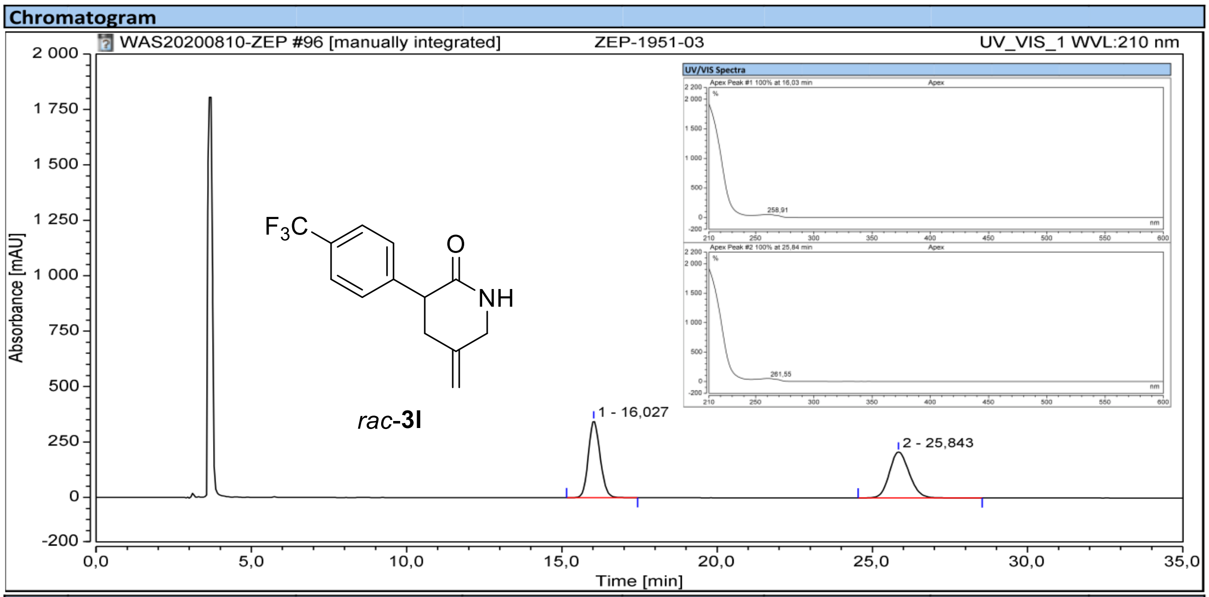

Integration Results							
No.	Peak Name	Retention Time min	Area mAU*min	Height mAU	Relative Area %	Relative Height %	Amount
n.a.	Peak 1	n.a.	n.a.	n.a.	n.a.	n.a.	n.a.
1		16,027	152,477	346,009	49,86	62,53	n.a.
2		25,843	153,322	207,316	50,14	37,47	n.a.
**Total:**			**305,799**	**553,325**	**100,00**	**100,00**	

HPLC traces of compound **3m**

CHIRALPAK OD-H, eluent: *n*-hexane:*i*-PrOH = 4/1, 1.0 mL·min^{-1}, 10 °C, λ = 210 nm

Chromatogram

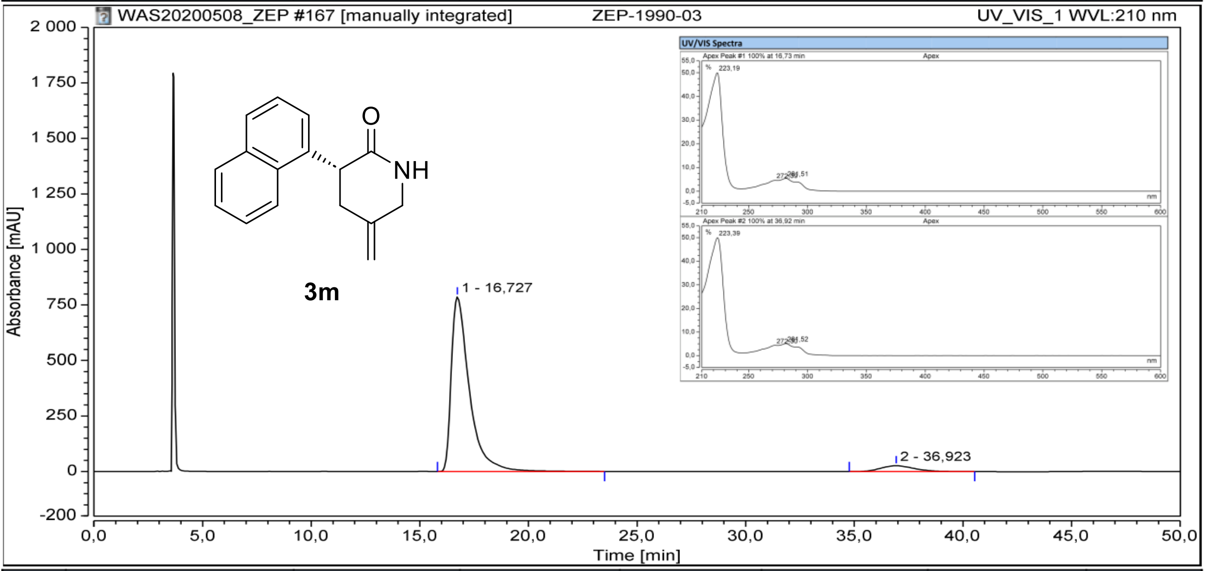

Integration Results

No.	Peak Name	Retention Time min	Area mAU*min	Height mAU	Relative Area %	Relative Height %	Amount
n.a.	Peak 1	n.a.	n.a.	n.a.	n.a.	n.a.	n.a.
1		16,727	759,417	786,282	93,95	96,73	n.a.
2		36,923	48,903	26,599	6,05	3,27	n.a.
**Total:**			**808,320**	**812,880**	**100,00**	**100,00**	

Chromatogram

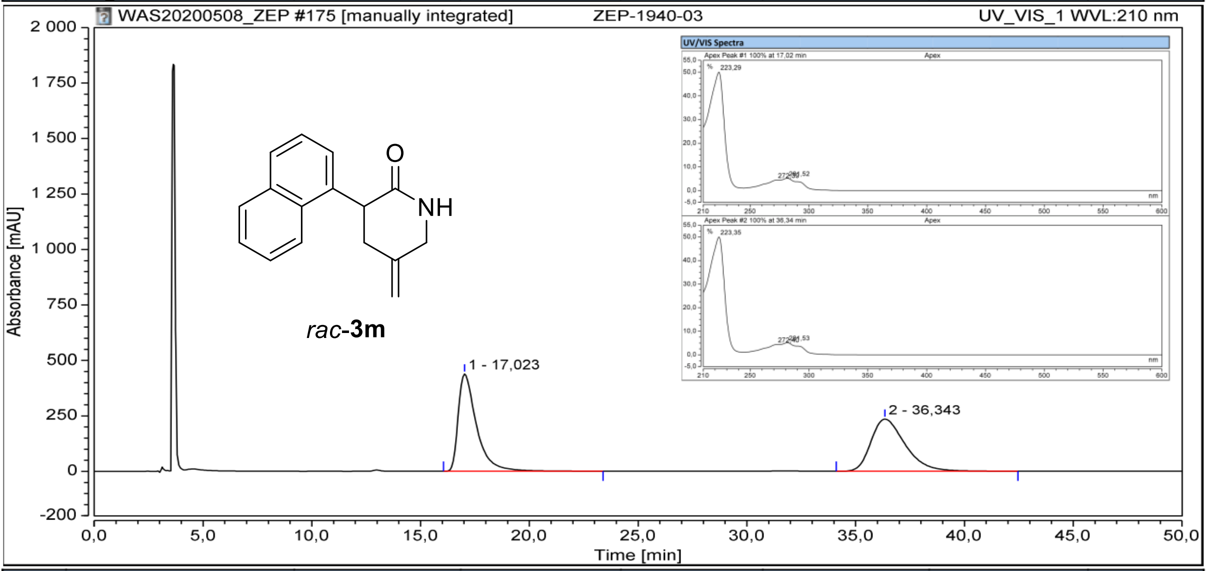

Integration Results

No.	Peak Name	Retention Time min	Area mAU*min	Height mAU	Relative Area %	Relative Height %	Amount
n.a.	Peak 1	n.a.	n.a.	n.a.	n.a.	n.a.	n.a.
1		17,023	428,120	438,475	49,98	65,11	n.a.
2		36,343	428,445	234,949	50,02	34,89	n.a.
**Total:**			**856,565**	**673,424**	**100,00**	**100,00**	

<u>HPLC traces</u> <u>of compound **3n**</u>

CHIRALPAK OD-H, eluent: *n*-hexane:*i*-PrOH = 4/1, 1.0 mL·min^{-1}, 10 °C, λ = 210 nm

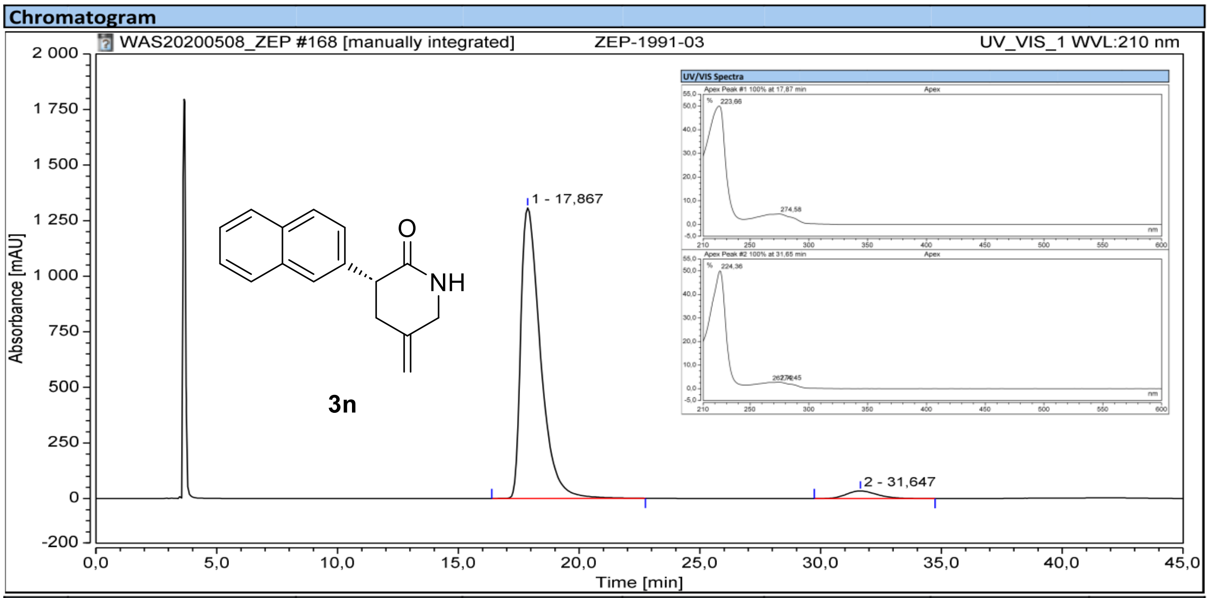

Integration Results

No.	Peak Name	Retention Time min	Area mAU*min	Height mAU	Relative Area %	Relative Height %	Amount
n.a.	Peak 1	n.a.	n.a.	n.a.	n.a.	n.a.	n.a.
1		17,867	1228,638	1308,005	96,00	97,44	n.a.
2		31,647	51,182	34,351	4,00	2,56	n.a.
**Total:**			**1279,820**	**1342,355**	**100,00**	**100,00**	

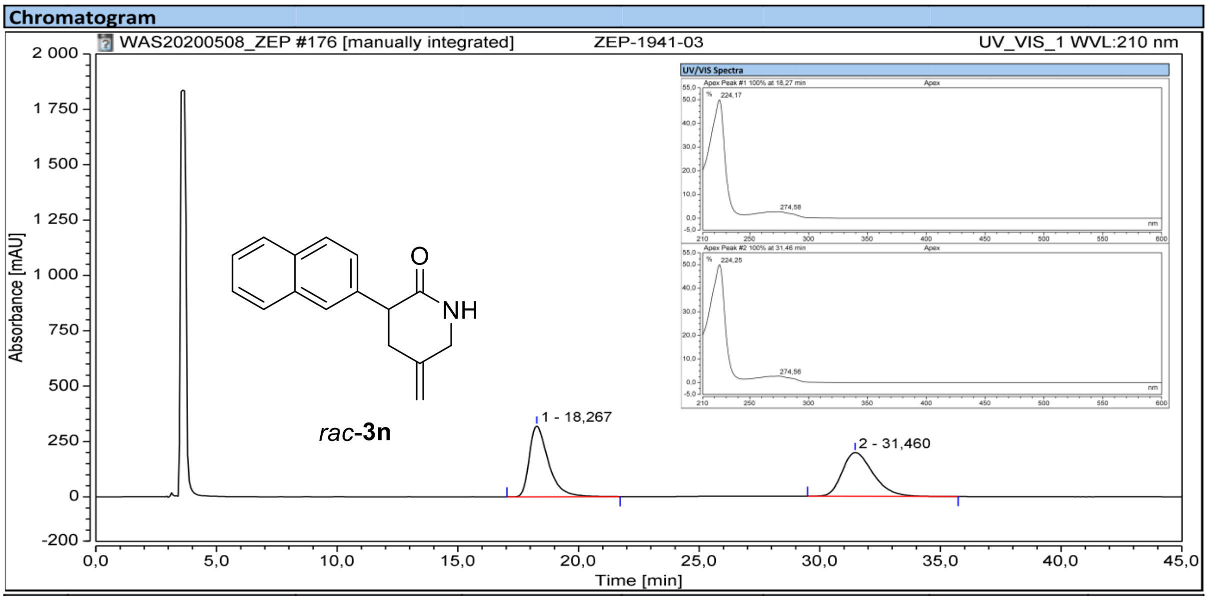

Integration Results

No.	Peak Name	Retention Time min	Area mAU*min	Height mAU	Relative Area %	Relative Height %	Amount
n.a.	Peak 1	n.a.	n.a.	n.a.	n.a.	n.a.	n.a.
1		18,267	294,086	319,785	50,03	61,75	n.a.
2		31,460	293,756	198,074	49,97	38,25	n.a.
**Total:**			**587,842**	**517,860**	**100,00**	**100,00**	

HPLC traces of compound **3o**

CHIRALPAK OD-H, eluent: *n*-hexane:*i*-PrOH = 4/1, 1.0 mL·min^{-1}, 10 °C, λ = 210 nm

Chromatogram

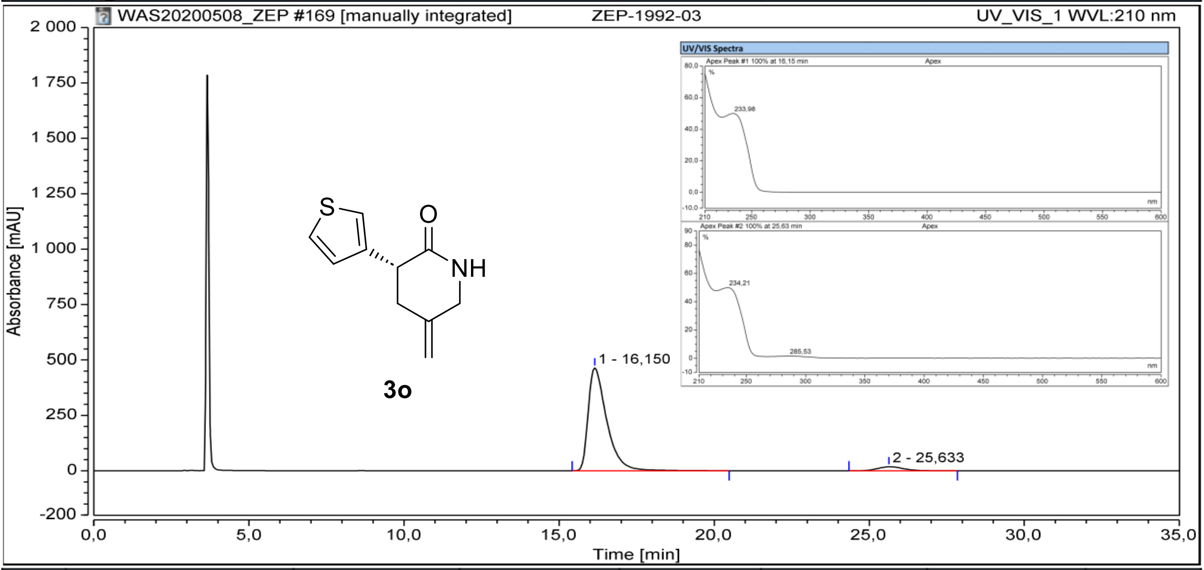

Integration Results

No.	Peak Name	Retention Time min	Area mAU*min	Height mAU	Relative Area %	Relative Height %	Amount
n.a.	Peak 1	n.a.	n.a.	n.a.	n.a.	n.a.	n.a.
1		16,150	322,505	463,900	94,54	96,16	n.a.
2		25,633	18,610	18,547	5,46	3,84	n.a.
**Total:**			**341,115**	**482,447**	**100,00**	**100,00**	

Chromatogram

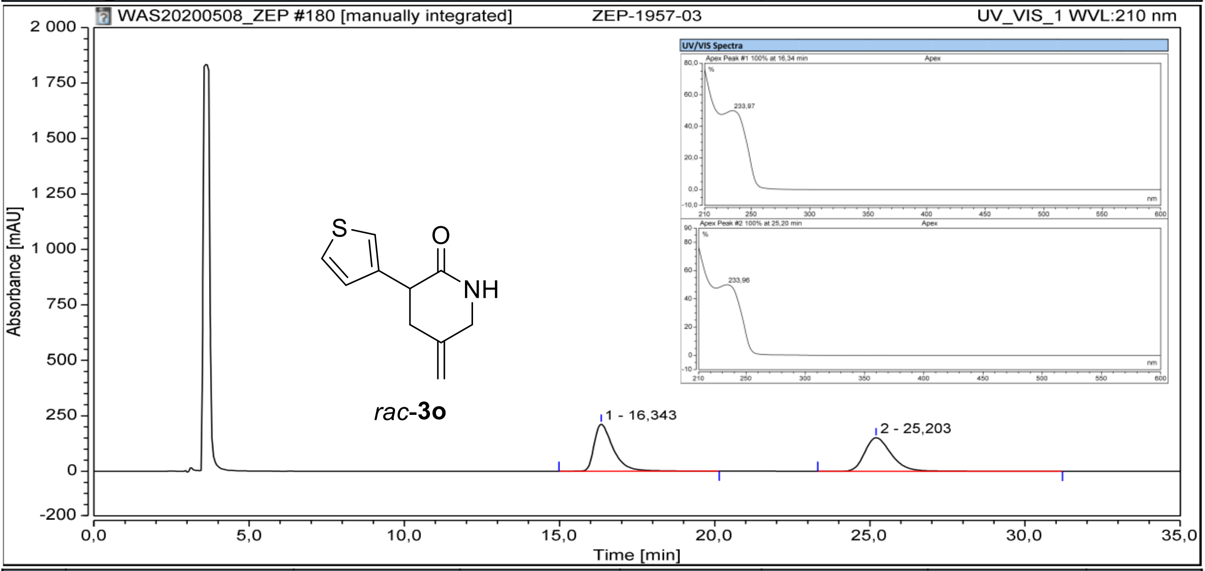

Integration Results

No.	Peak Name	Retention Time min	Area mAU*min	Height mAU	Relative Area %	Relative Height %	Amount
n.a.	Peak 1	n.a.	n.a.	n.a.	n.a.	n.a.	n.a.
1		16,343	151,951	212,096	50,14	58,37	n.a.
2		25,203	151,092	151,250	49,86	41,63	n.a.
**Total:**			**303,043**	**363,346**	**100,00**	**100,00**	

HPLC traces of compound **3p**

YMC Chiral ART Cellulose-SB, eluent: *n*-hexane:*i*-PrOH = 4/1, 1.0 mL·min^{-1}, 10 °C, λ = 210 nm

Chromatogram

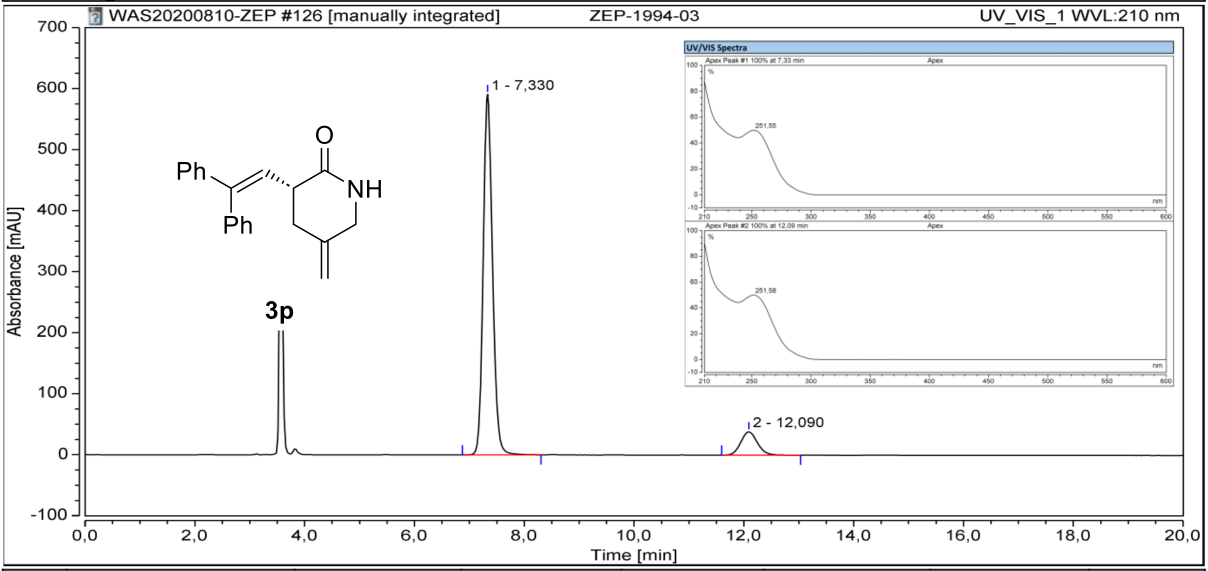

Integration Results

No.	Peak Name	Retention Time min	Area mAU*min	Height mAU	Relative Area %	Relative Height %	Amount
n.a.	Peak 1	n.a.	n.a.	n.a.	n.a.	n.a.	n.a.
1		7,330	115,415	590,774	89,60	93,92	n.a.
2		12,090	13,390	38,266	10,40	6,08	n.a.
**Total:**			**128,806**	**629,039**	**100,00**	**100,00**	

Chromatogram

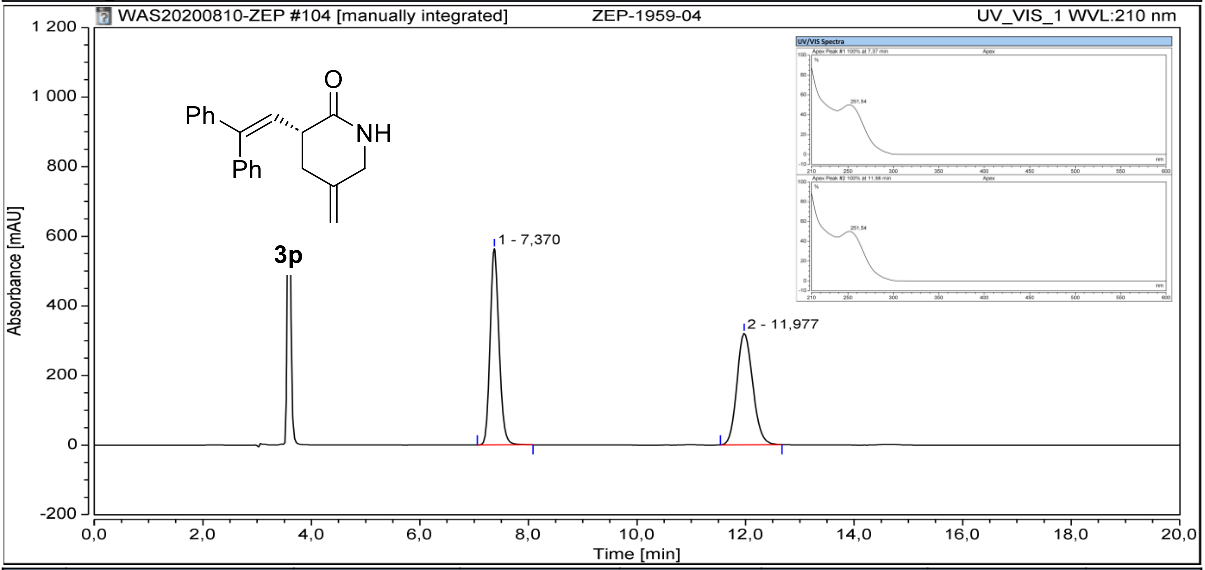

Integration Results

No.	Peak Name	Retention Time min	Area mAU*min	Height mAU	Relative Area %	Relative Height %	Amount
n.a.	Peak 1	n.a.	n.a.	n.a.	n.a.	n.a.	n.a.
1		7,370	108,293	563,484	49,97	63,74	n.a.
2		11,977	108,423	320,494	50,03	36,26	n.a.
**Total:**			**216,716**	**883,978**	**100,00**	**100,00**	

HPLC traces of compound **4a**

YMC Chiral ART Amylose-SA, eluent: *n*-hexane:*i*-PrOH = 50/1, 0.7 mL·min^{-1}, 10 °C, λ = 210 nm

Chromatogram

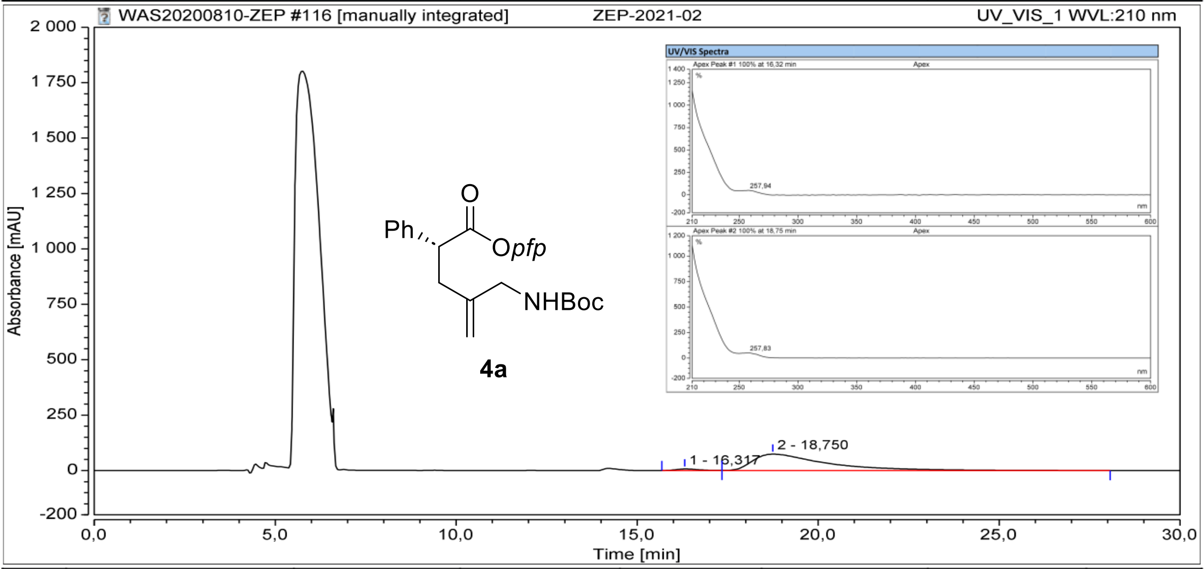

Integration Results

No.	Peak Name	Retention Time min	Area mAU*min	Height mAU	Relative Area %	Relative Height %	Amount
n.a.	Peak 1	n.a.	n.a.	n.a.	n.a.	n.a.	n.a.
1		16,317	5,544	7,289	3,02	8,93	n.a.
2		18,750	177,998	74,332	96,98	91,07	n.a.
**Total:**			**183,543**	**81,620**	**100,00**	**100,00**	

Chromatogram

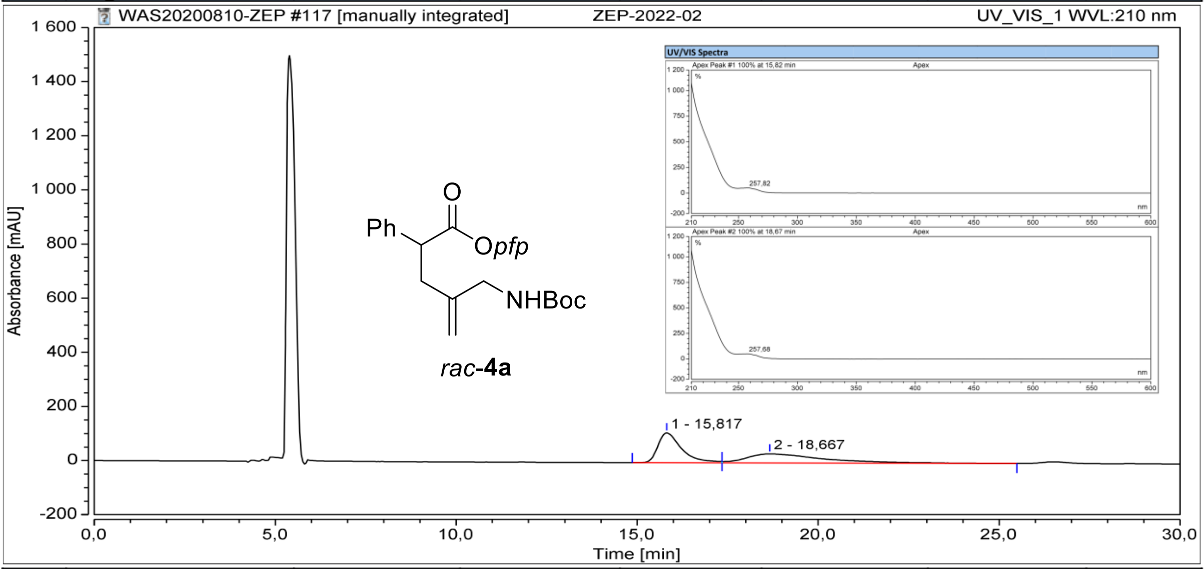

Integration Results

No.	Peak Name	Retention Time min	Area mAU*min	Height mAU	Relative Area %	Relative Height %	Amount
n.a.	Peak 1	n.a.	n.a.	n.a.	n.a.	n.a.	n.a.
1		15,817	83,478	110,176	50,35	76,65	n.a.
2		18,667	82,315	33,566	49,65	23,35	n.a.
**Total:**			**165,793**	**143,742**	**100,00**	**100,00**	

HPLC traces of compound **6a**

YMC Chiral ART Cellulose-SB, eluent: *n*-hexane:*i*-PrOH = 1/1, 1.0 mL·min^{-1}, 10 °C, λ = 210 nm

Chromatogram

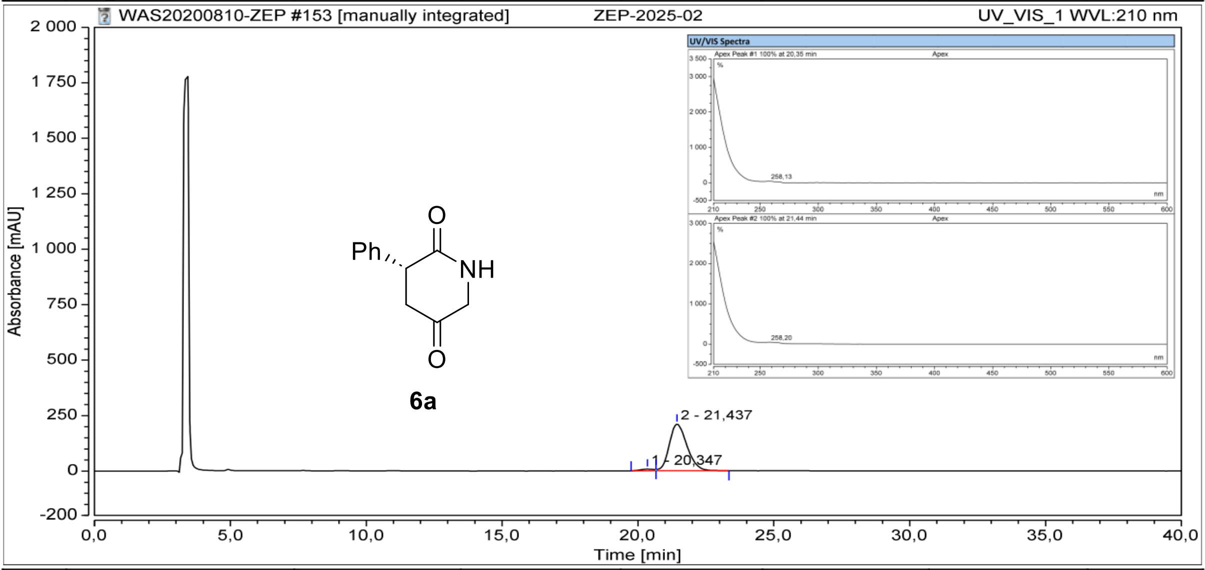

Integration Results

No.	Peak Name	Retention Time min	Area mAU*min	Height mAU	Relative Area %	Relative Height %	Amount
n.a.	Peak 1	n.a.	n.a.	n.a.	n.a.	n.a.	n.a.
1		20,347	4,415	7,682	2,71	3,53	n.a.
2		21,437	158,635	210,171	97,29	96,47	n.a.
**Total:**			**163,050**	**217,852**	**100,00**	**100,00**	

Chromatogram

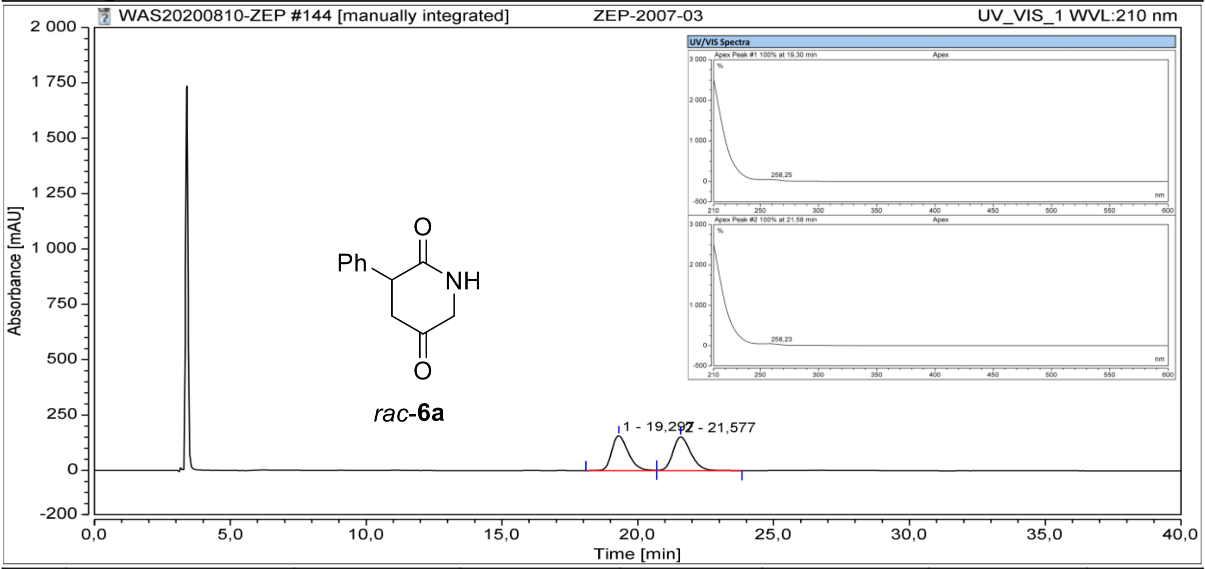

Integration Results

No.	Peak Name	Retention Time min	Area mAU*min	Height mAU	Relative Area %	Relative Height %	Amount
n.a.	Peak 1	n.a.	n.a.	n.a.	n.a.	n.a.	n.a.
1		19,297	110,292	157,274	48,77	50,88	n.a.
2		21,577	115,862	151,818	51,23	49,12	n.a.
**Total:**			**226,155**	**309,092**	**100,00**	**100,00**	

HPLC traces of compound 7a

YMC Chiral ART Cellulose-SB, eluent: *n*-hexane:*i*-PrOH = 4/1, 0.5 mL·min^{-1}, 10 °C, λ = 210 nm

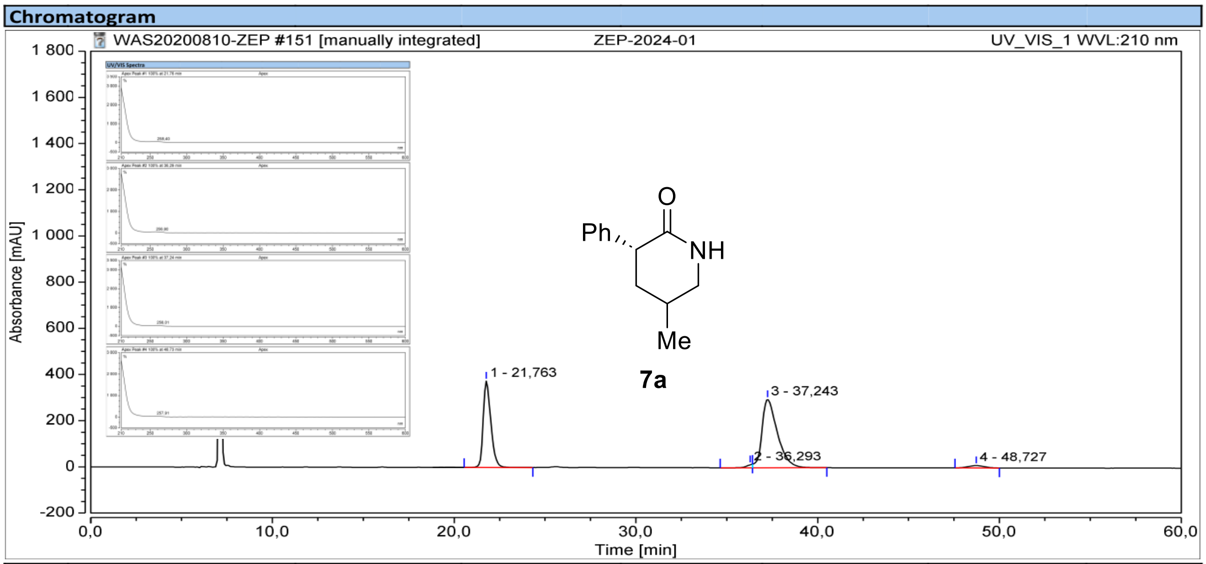

Integration Results

No.	Peak Name	Retention Time min	Area mAU*min	Height mAU	Relative Area %	Relative Height %	Amount
n.a.	Peak 1	n.a.	n.a.	n.a.	n.a.	n.a.	n.a.
1		21,763	184,573	374,338	37,48	53,95	n.a.
2		36,293	5,231	13,164	1,06	1,90	n.a.
3		37,243	292,283	296,147	59,35	42,68	n.a.
4		48,727	10,378	10,260	2,11	1,48	n.a.
**Total:**			**492,464**	**693,908**	**100,00**	**100,00**	

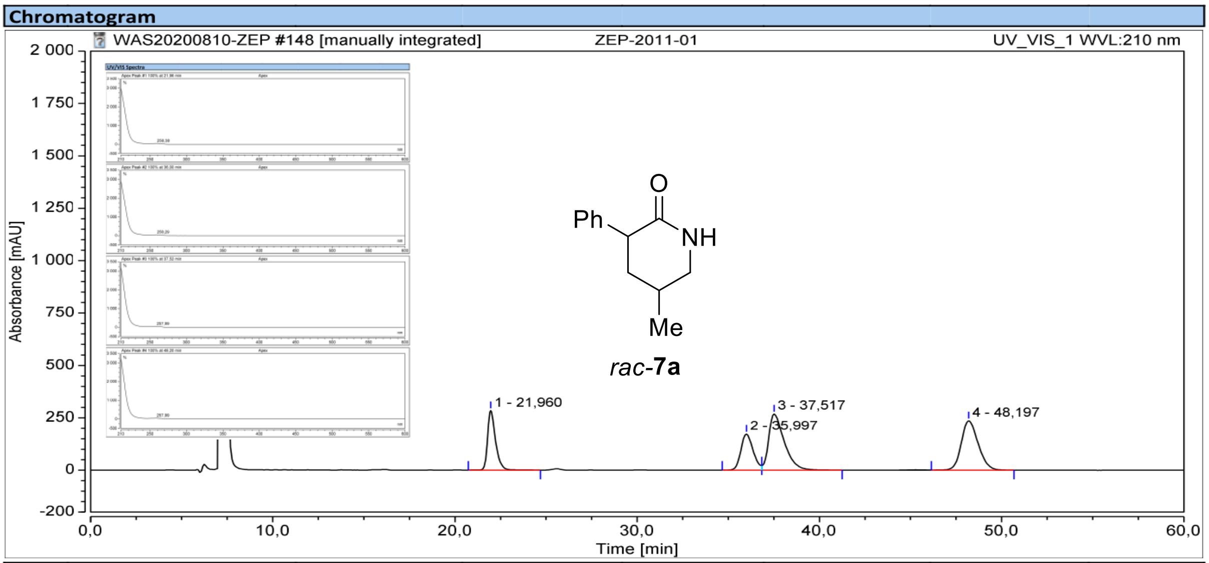

Integration Results

No.	Peak Name	Retention Time min	Area mAU*min	Height mAU	Relative Area %	Relative Height %	Amount
n.a.	Peak 1	n.a.	n.a.	n.a.	n.a.	n.a.	n.a.
1		21,960	144,827	283,950	18,05	29,52	n.a.
2		35,997	134,037	173,394	16,71	18,03	n.a.
3		37,517	268,379	268,500	33,45	27,92	n.a.
4		48,197	255,089	235,929	31,79	24,53	n.a.
**Total:**			**802,332**	**961,773**	**100,00**	**100,00**	

HPLC traces of compound **8a**

YMC Chiral ART Cellulose-SB, eluent: *n*-hexane:*i*-PrOH = 1/1, 1.0 mL·min^{-1}, 10 °C, λ = 210 nm

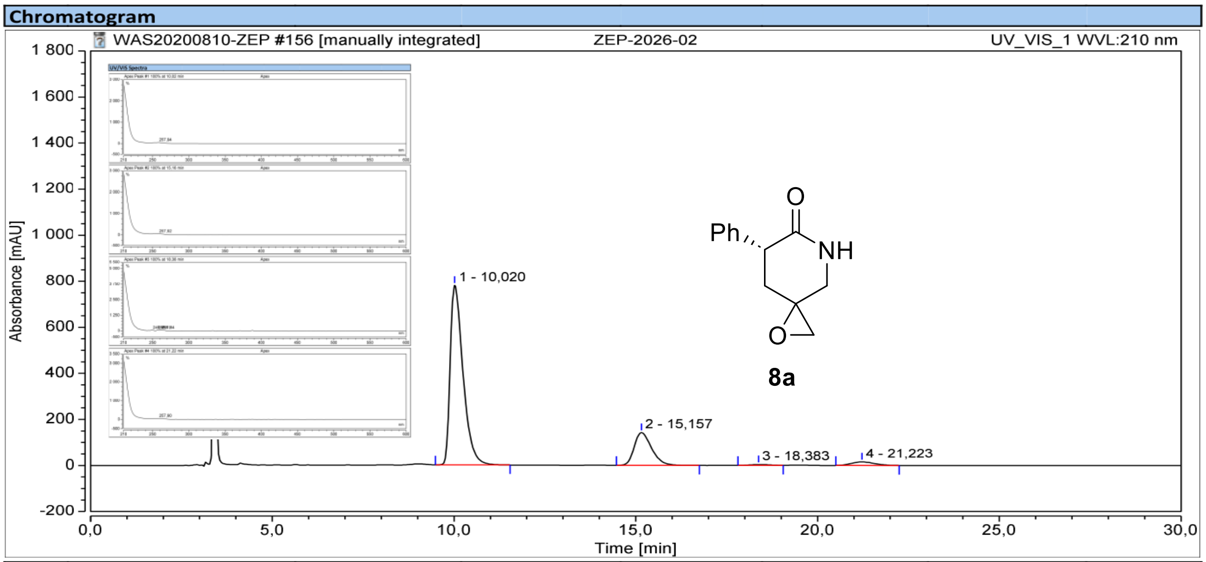

Integration Results

No.	Peak Name	Retention Time min	Area mAU*min	Height mAU	Relative Area %	Relative Height %	Amount
n.a.	Peak 1	n.a.	n.a.	n.a.	n.a.	n.a.	n.a.
1		10,020	319,651	780,185	77,56	82,89	n.a.
2		15,157	78,949	142,182	19,16	15,11	n.a.
3		18,383	2,262	3,571	0,55	0,38	n.a.
4		21,223	11,254	15,244	2,73	1,62	n.a.
**Total:**			**412,116**	**941,183**	**100,00**	**100,00**	

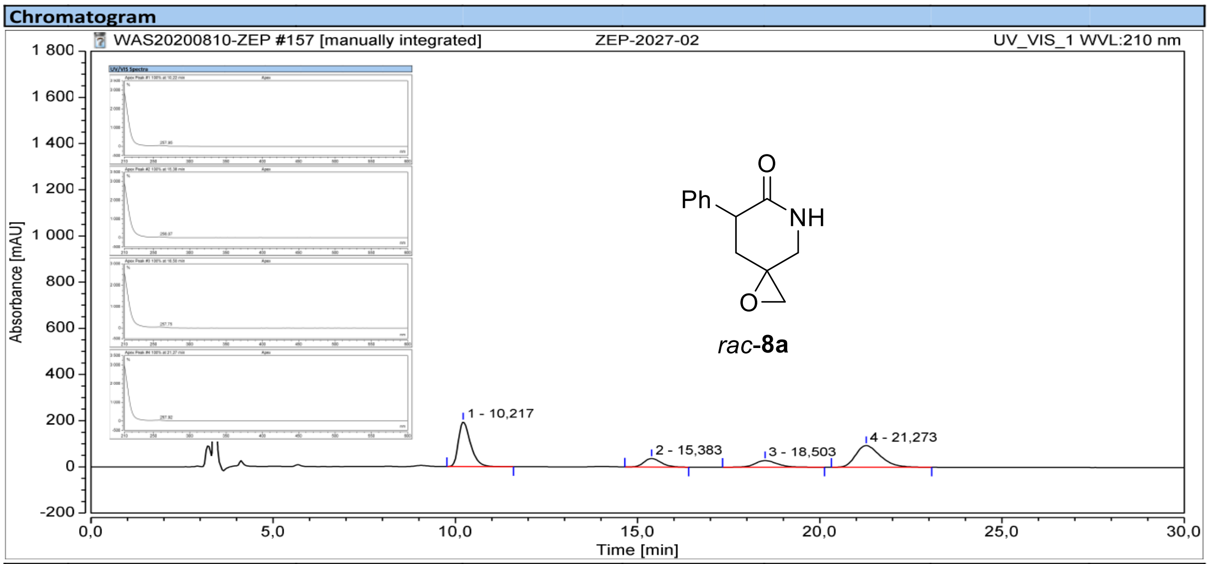

Integration Results

No.	Peak Name	Retention Time min	Area mAU*min	Height mAU	Relative Area %	Relative Height %	Amount
n.a.	Peak 1	n.a.	n.a.	n.a.	n.a.	n.a.	n.a.
1		10,217	74,882	193,252	39,58	54,75	n.a.
2		15,383	20,031	37,041	10,59	10,49	n.a.
3		18,503	20,313	28,820	10,74	8,17	n.a.
4		21,273	73,982	93,857	39,10	26,59	n.a.
**Total:**			**189,208**	**352,970**	**100,00**	**100,00**	

8. HRMS Data

HRMS spectrum of compound **1f**

ESI-QTOF, MeOH, calcd m/z for $C_{15}H_{13}F_5NO_2S^+$: 366.0582 [*M*+NH4]+; found: 366.0578.

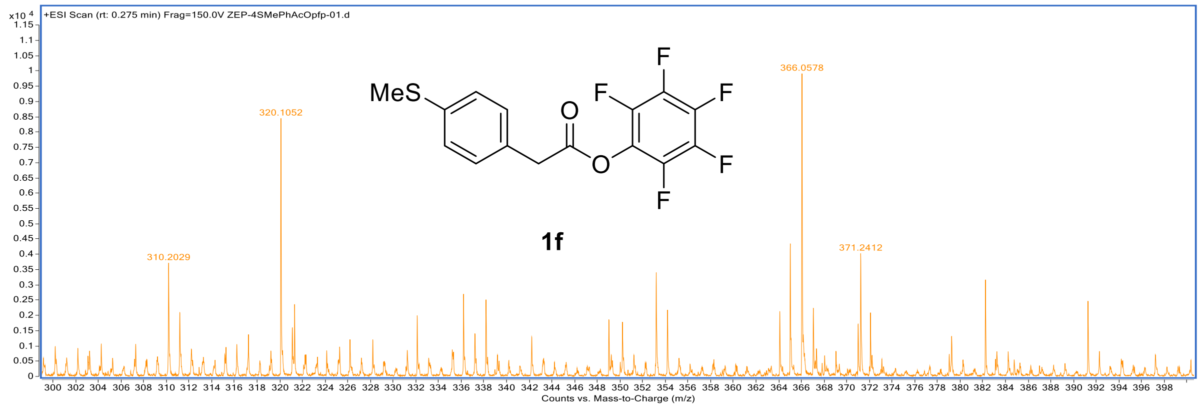

HRMS spectrum of compound **1p**

ESI-QTOF, MeOH, calcd *m/z* for $C_{22}H_{14}F_5O_2^+$: 405.0908 [*M*+H]+; found: 405.0914.

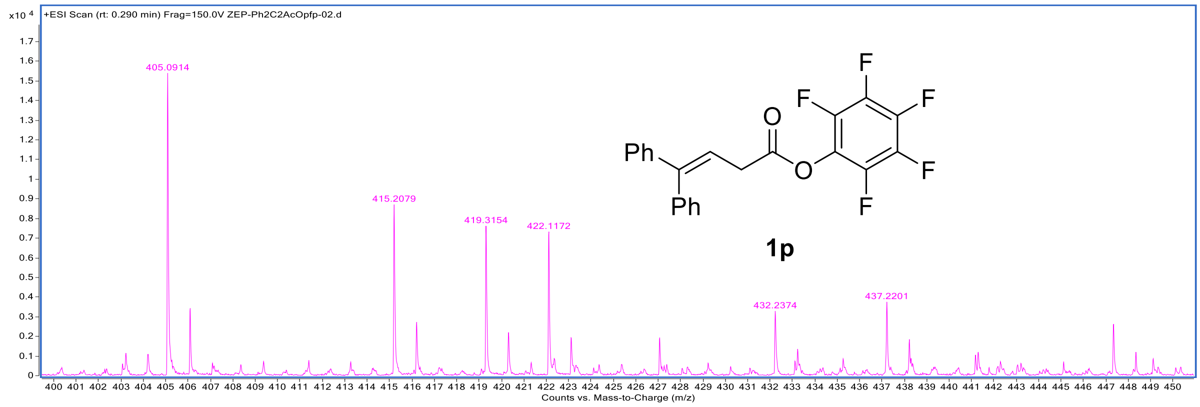

HRMS spectrum of compound **2a**

ESI-QTOF, MeOH, calcd *m/z* for $C_{10}H_{19}NNaO_5S^+$: 288.0876 [*M*+Na]+; found: 288.0878.

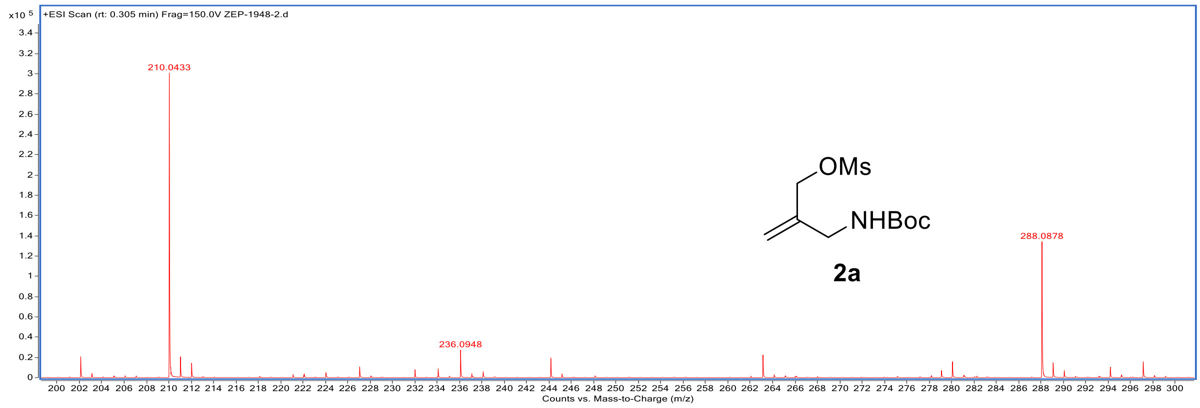

HRMS spectrum of compound **2b**

ESI-QTOF, MeOH, calcd *m/z* for $C_{11}H_{19}NNaO_4^+$: 252.1206 [*M*+Na]$^+$; found: 252.1208.

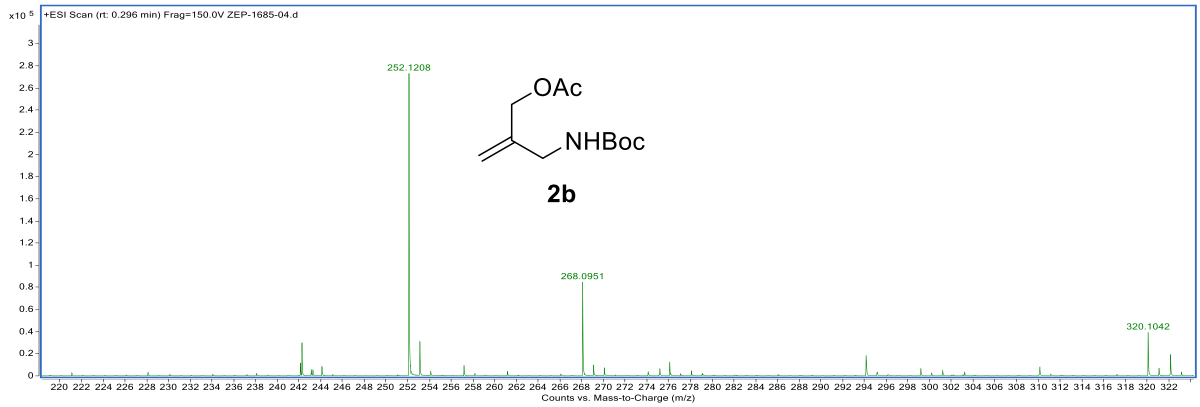

HRMS spectrum of compound **2c**

ESI-QTOF, MeOH, calcd *m/z* for $C_{11}H_{19}NNaO_5^+$: 268.1155 [*M*+Na]$^+$; found: 268.1153.

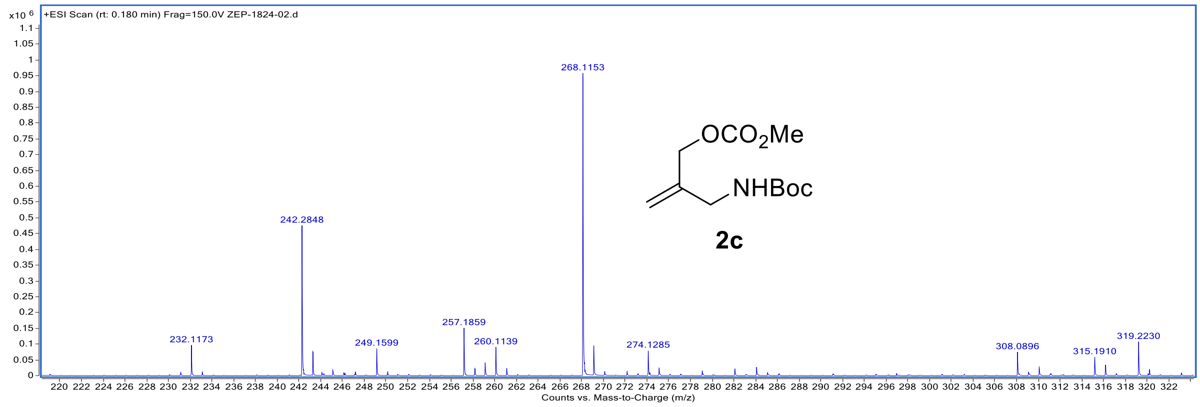

HRMS spectrum of compound **2d**

ESI-QTOF, MeOH, calcd *m/z* for $C_{21}H_{26}NNaO_6P^+$: 442.1390 [*M*+Na]$^+$; found: 442.1393.

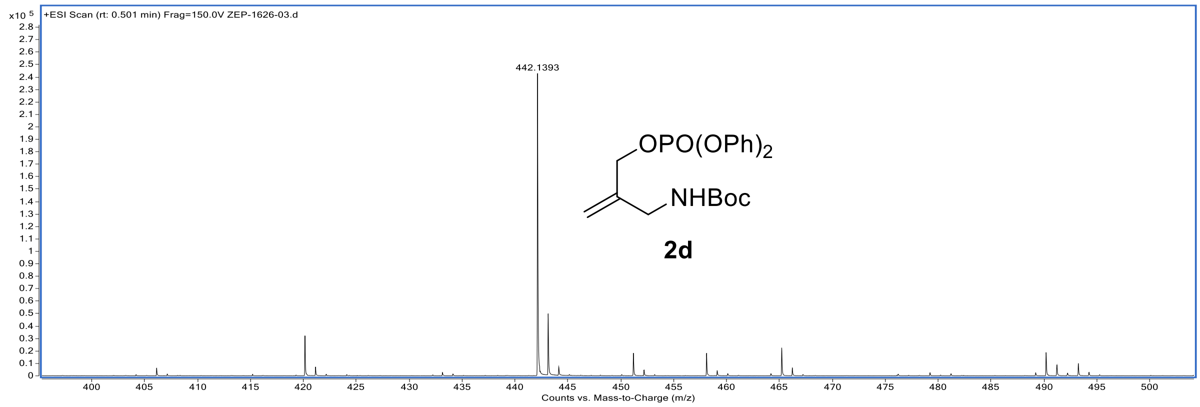

HRMS spectrum of compound **2e**

ESI-QTOF, MeOH, calcd *m/z* for $C_{14}H_{25}NNaO_4^+$: 294.1676 [*M*+Na]$^+$; found: 294.1679.

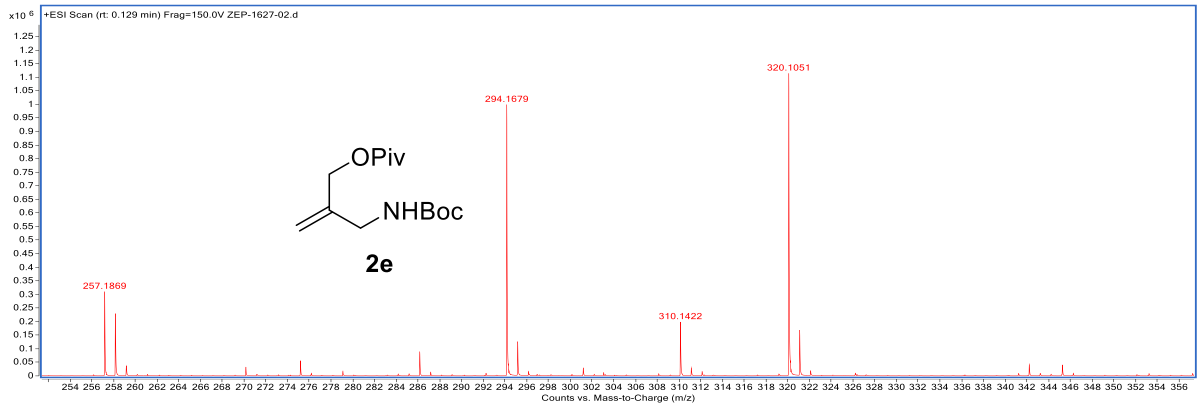

HRMS spectrum of compound **3a**

ESI-QTOF, MeOH, calcd *m/z* for $C_{12}H_{14}NO^+$: 188.1070 [*M*+H]$^+$; found: 188.1071.

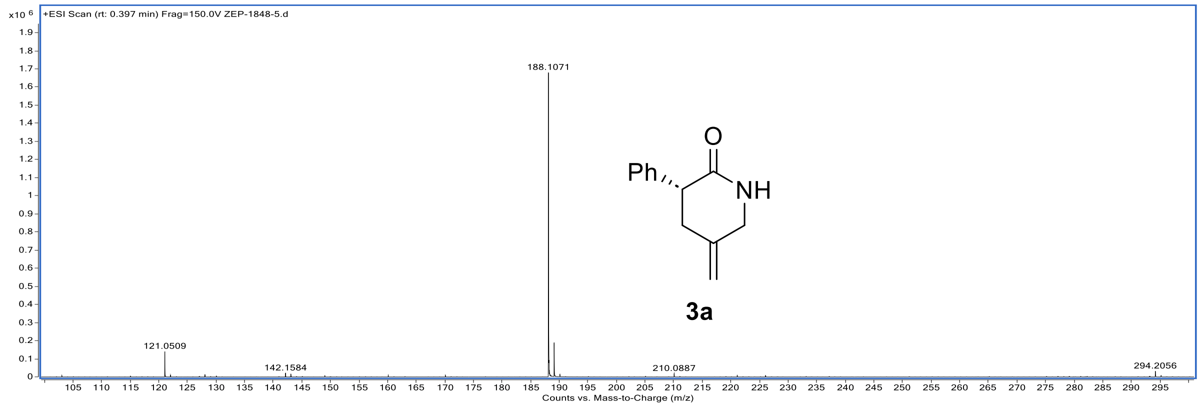

HRMS spectrum of compound **3b**

ESI-QTOF, MeOH, calcd *m/z* for $C_{12}H_{13}FNO^+$: 206.0976 [*M*+H]$^+$; found: 206.0978.

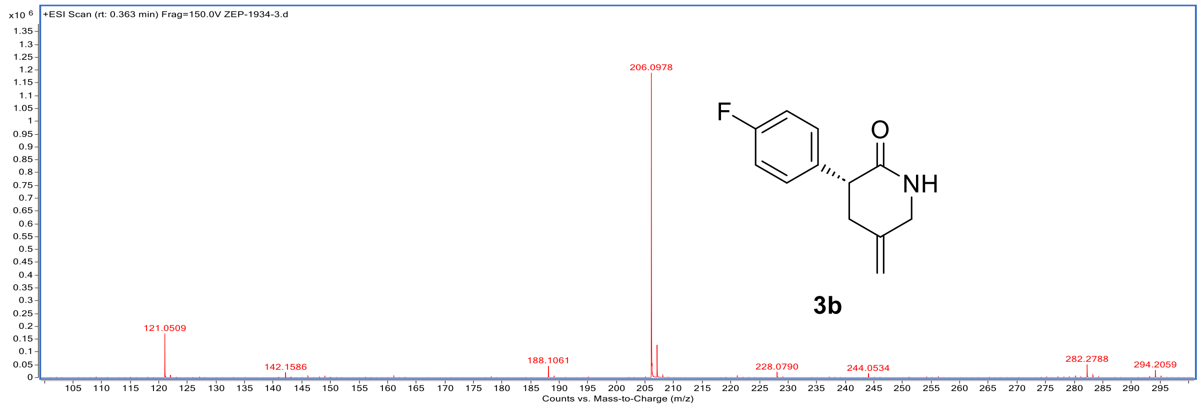

<u>**HRMS spectrum**</u> <u>of compound **3c**</u>

ESI-QTOF, MeOH, calcd *m/z* for $C_{12}H_{13}ClNO^+$: 222.0680 [*M*+H]$^+$; found: 222.0683.

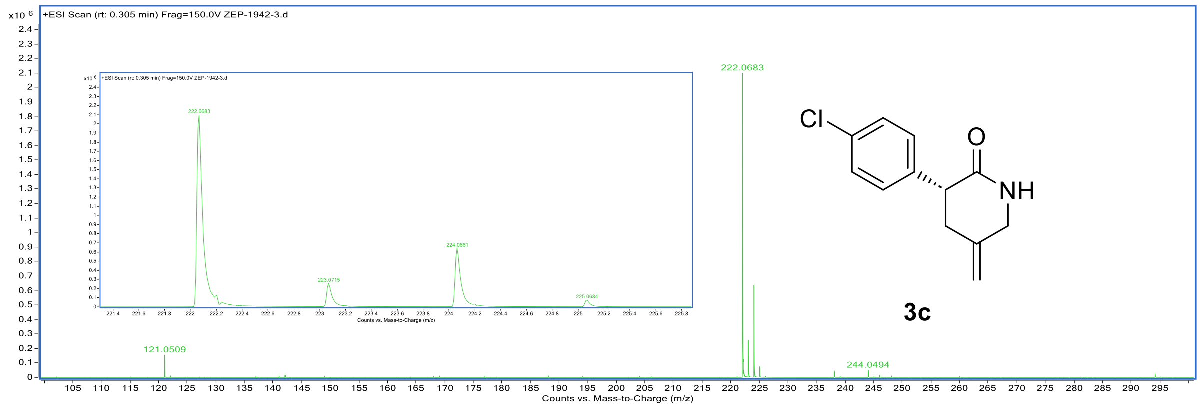

<u>**HRMS spectrum**</u> <u>of compound **3d**</u>

ESI-QTOF, MeOH, calcd *m/z* for $C_{12}H_{13}BrNO^+$: 266.0175 [*M*+H]$^+$; found: 266.0179.

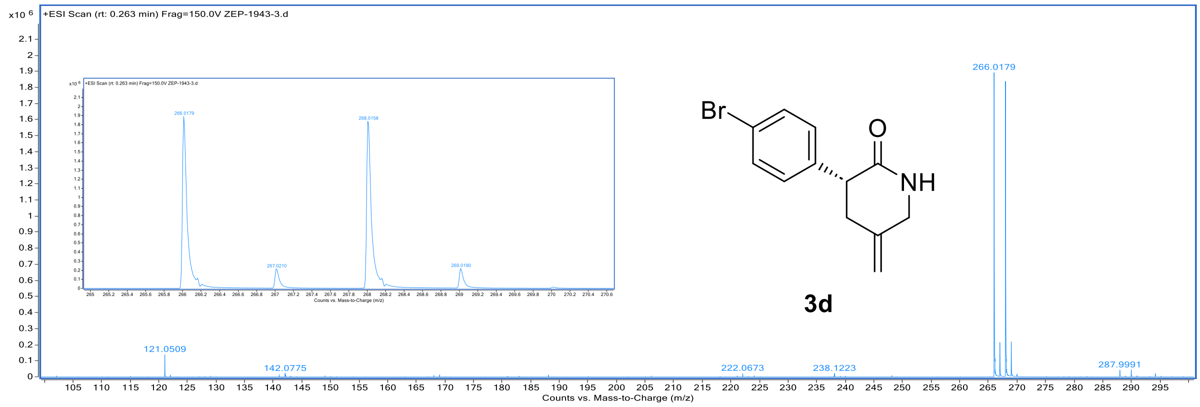

<u>**HRMS spectrum**</u> <u>of compound **3e**</u>

ESI-QTOF, MeOH, calcd *m/z* for $C_{13}H_{16}NO_2^+$: 218.1176 [*M*+H]$^+$; found: 218.1175.

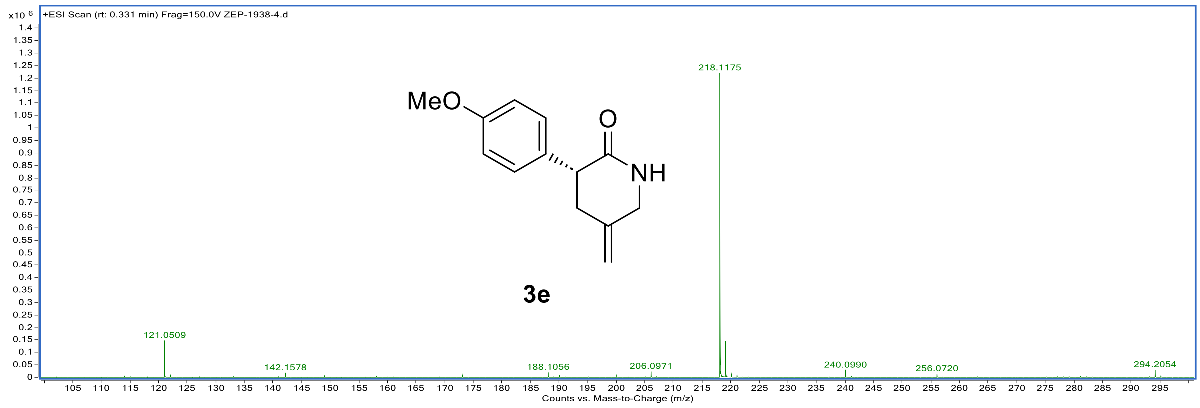

HRMS spectrum of compound **3f**

ESI-QTOF, MeOH, calcd *m/z* for $C_{13}H_{16}NOS^+$: 234.0947 [*M*+H]$^+$; found: 234.0948.

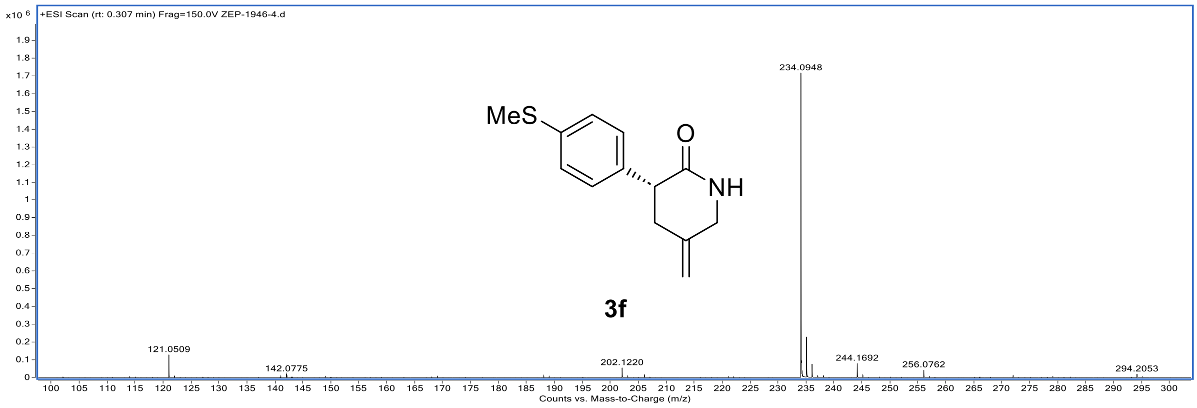

HRMS spectrum of compound **3g**

ESI-QTOF, MeOH, calcd *m/z* for $C_{16}H_{22}NO^+$: 244.1696 [*M*+H]$^+$; found: 244.1697.

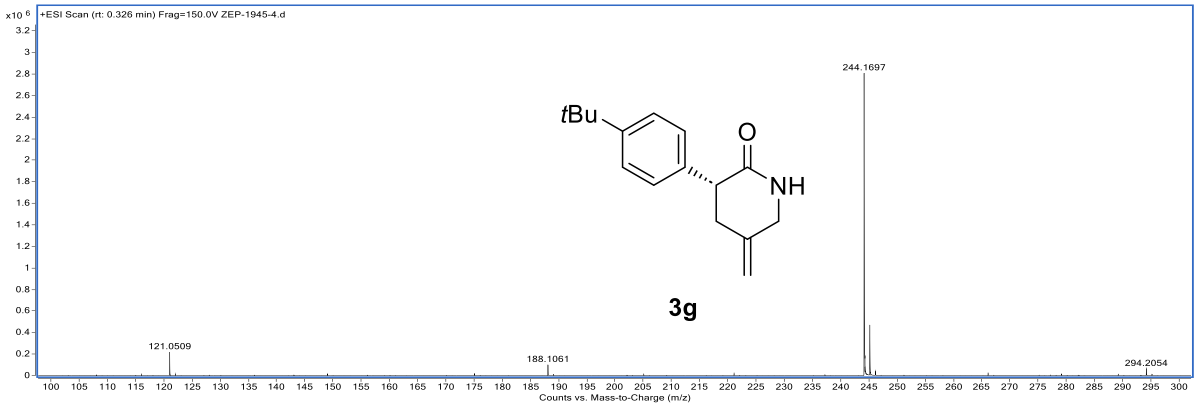

HRMS spectrum of compound **3h**

ESI-QTOF, MeOH, calcd *m/z* for $C_{13}H_{16}NO^+$: 202.1226 [*M*+H]$^+$; found: 202.1229.

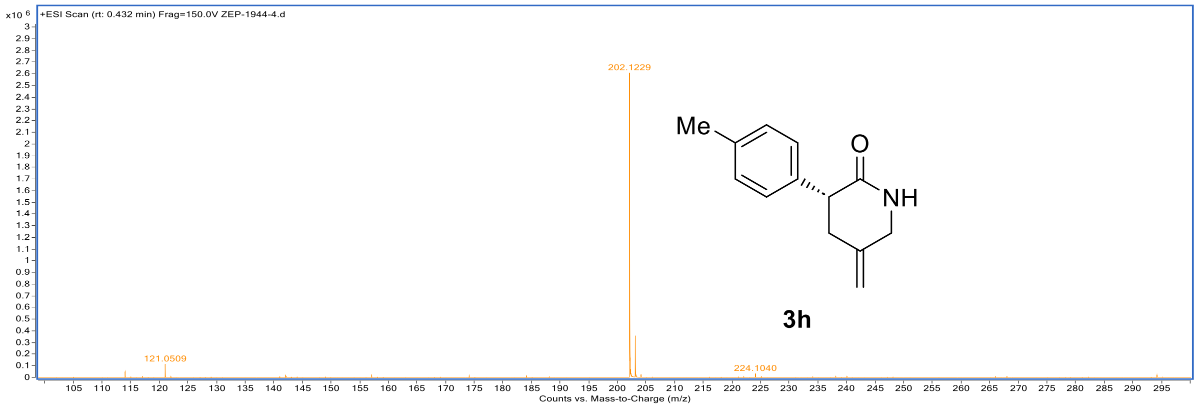

HRMS spectrum of compound **3i**

ESI-QTOF, MeOH, calcd *m/z* for $C_{13}H_{16}NO^+$: 202.1226 [*M*+H]$^+$; found: 202.1223.

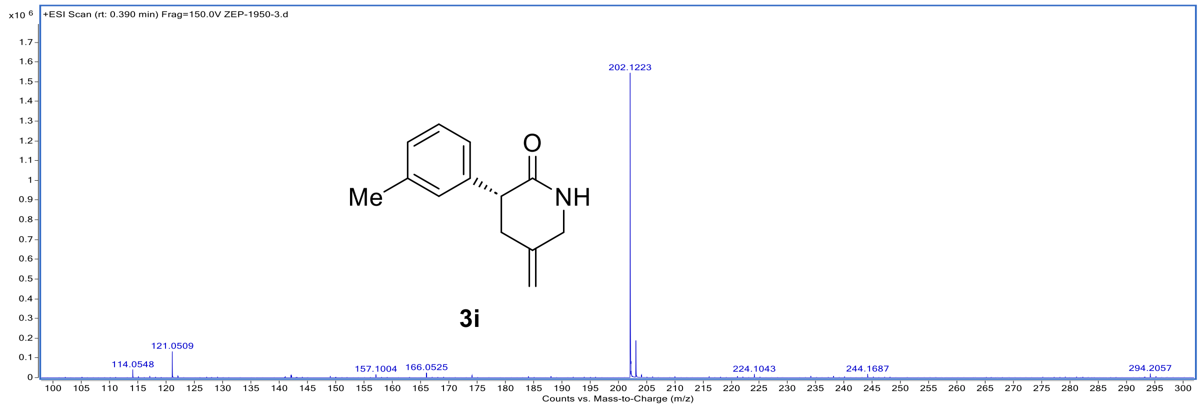

HRMS spectrum of compound **3j**

ESI-QTOF, MeOH, calcd *m/z* for $C_{13}H_{16}NO^+$: 202.1226 [*M*+H]$^+$; found: 202.1228.

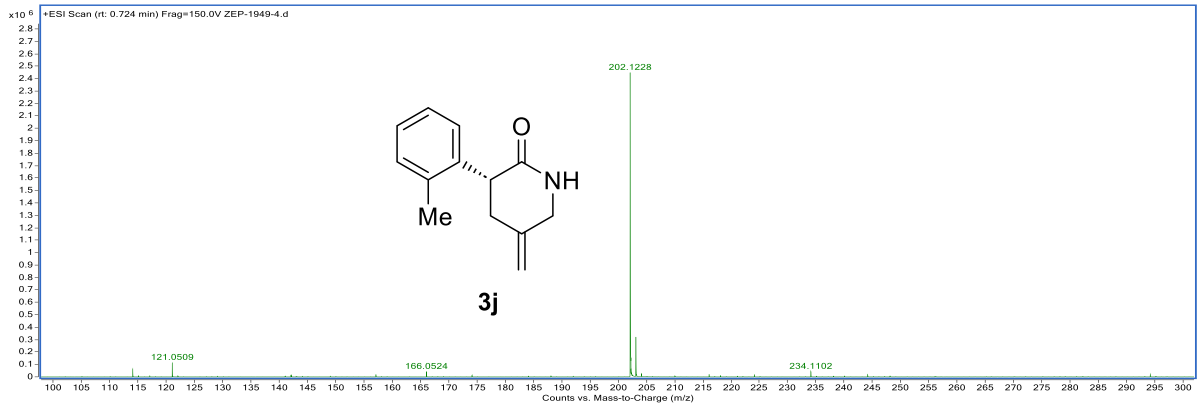

HRMS spectrum of compound **3k**

ESI-QTOF, MeOH, calcd *m/z* for $C_{14}H_{18}NO_3^+$: 248.1281 [*M*+H]$^+$; found: 248.1282.

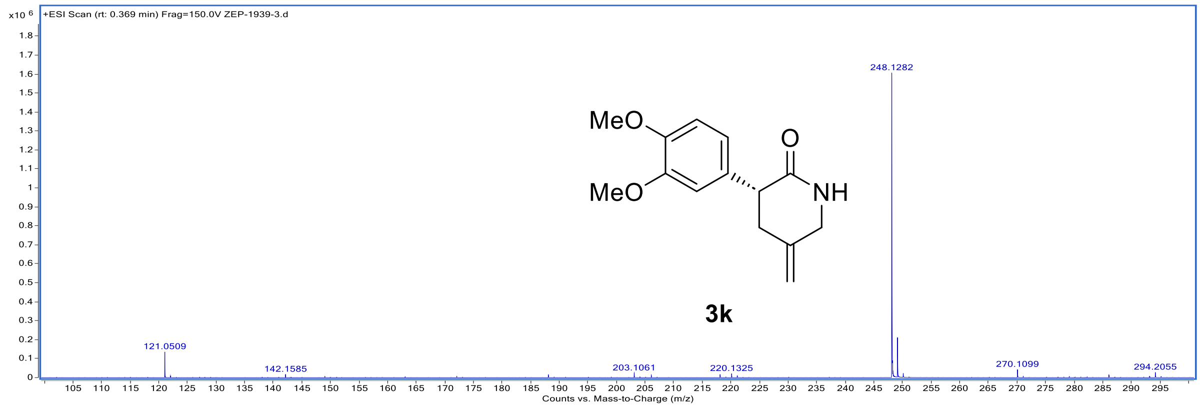

HRMS spectrum of compound **3l**

ESI-QTOF, MeOH, calcd *m/z* for $C_{13}H_{13}F_3NO^+$: 256.0944 [M+H]$^+$; found: 256.0946.

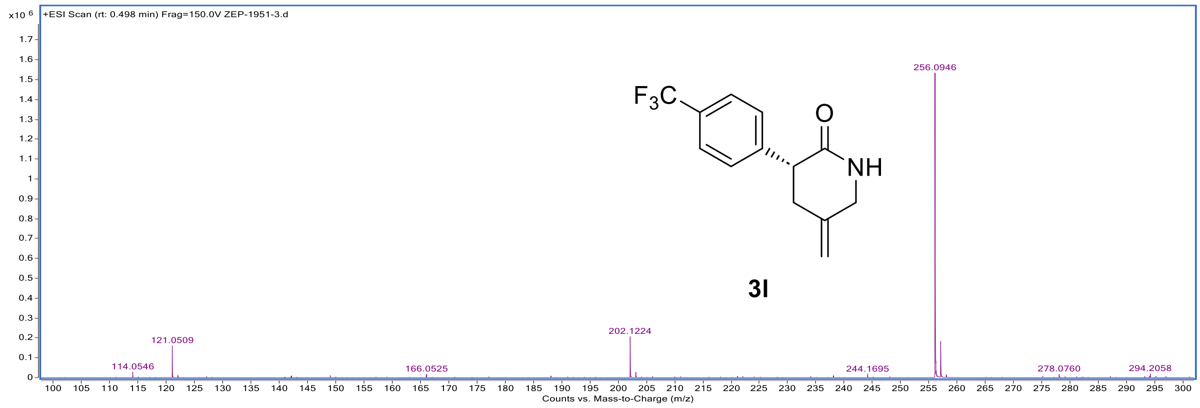

HRMS spectrum of compound **3m**

ESI-QTOF, MeOH, calcd *m/z* for $C_{16}H_{16}NO^+$: 238.1226 [M+H]$^+$; found: 238.1228.

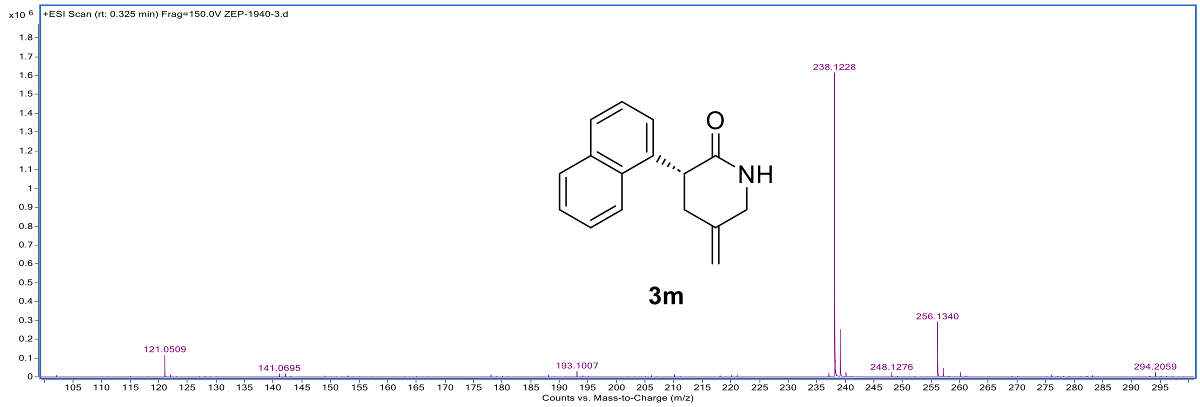

HRMS spectrum of compound **3n**

ESI-QTOF, MeOH, calcd *m/z* for $C_{16}H_{16}NO^+$: 238.1226 [M+H]$^+$; found: 238.1226.

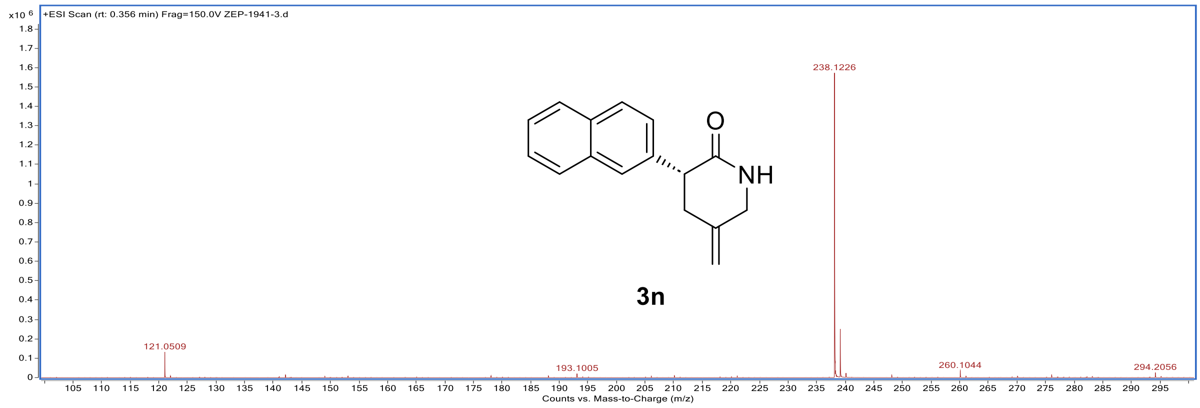

HRMS spectrum of compound **3o**

ESI-QTOF, MeOH, calcd *m/z* for $C_{10}H_{12}NOS^+$: 194.0634 [*M*+H]$^+$; found: 194.0635.

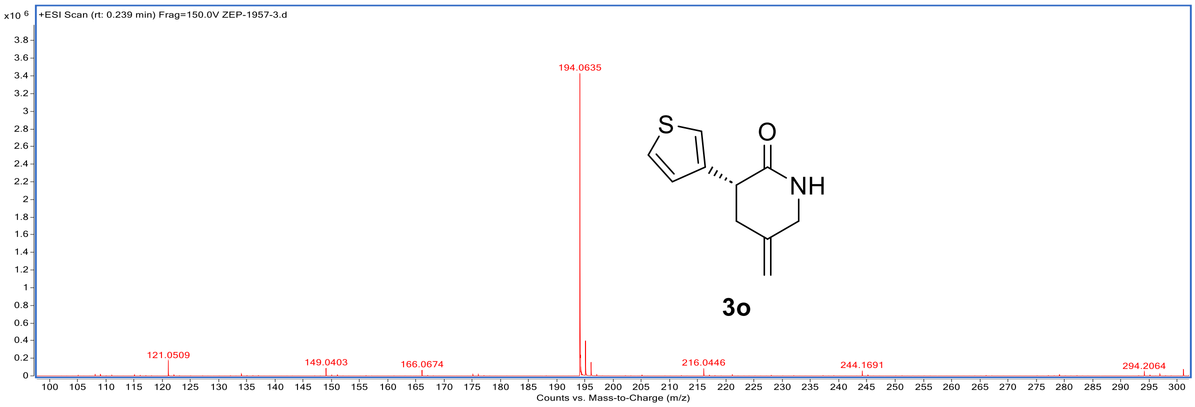

HRMS spectrum of compound **3p**

ESI-QTOF, MeOH, calcd *m/z* for $C_{20}H_{20}NO^+$: 290.1539 [*M*+H]$^+$; found: 290.1535.

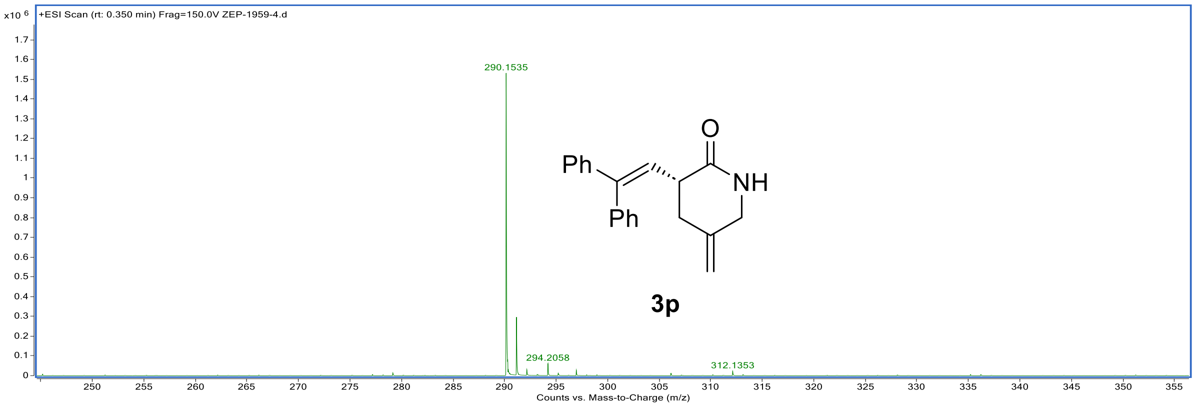

HRMS spectrum of compound **4a**

ESI-QTOF, MeOH, calcd *m/z* for $C_{23}H_{22}F_5NNaO_4^+$: 494.1361 [*M*+Na]$^+$; found: 494.1361.

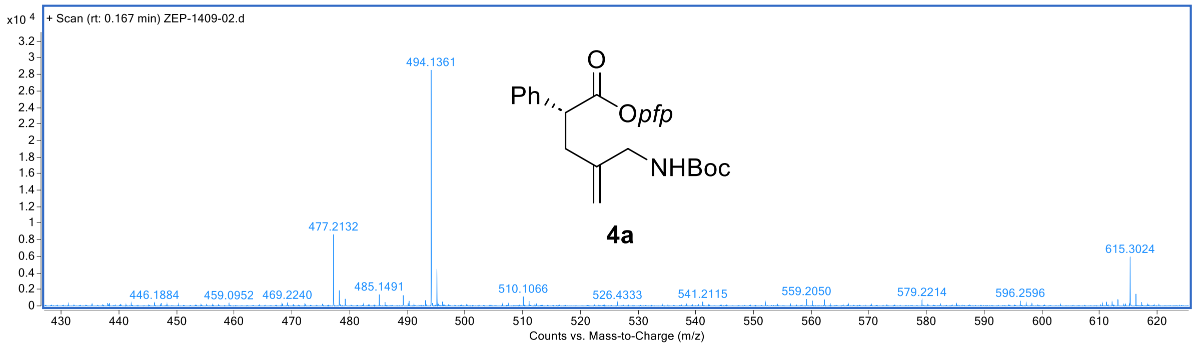

<u>HRMS spectrum</u> <u>of compound **6a**</u>

ESI-QTOF, MeOH, calcd *m/z* for $C_{11}H_{12}NO_2^+$: 190.0863 [*M*+H]$^+$; found: 190.0865.

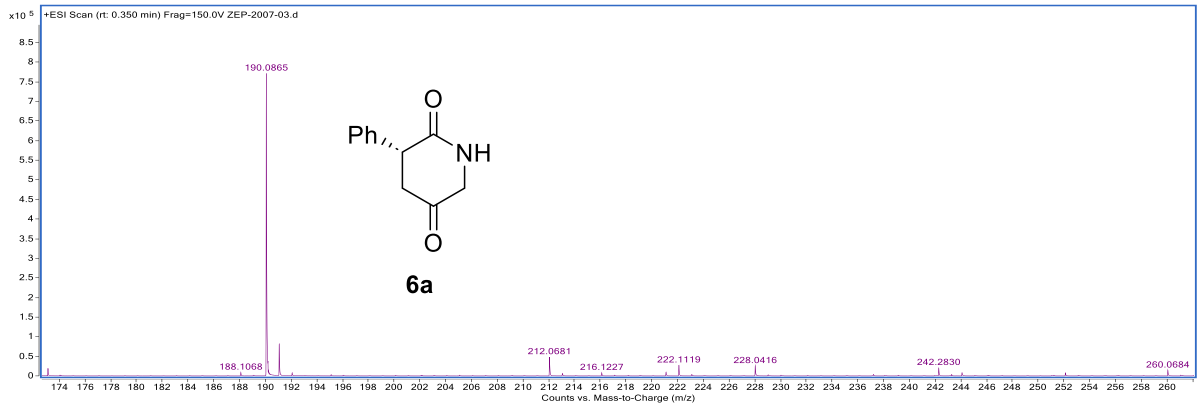

<u>HRMS spectrum</u> <u>of compound **7a**</u>

ESI-QTOF, MeOH, calcd *m/z* for $C_{12}H_{16}NO^+$: 190.1226 [*M*+H]$^+$; found: 190.1231.

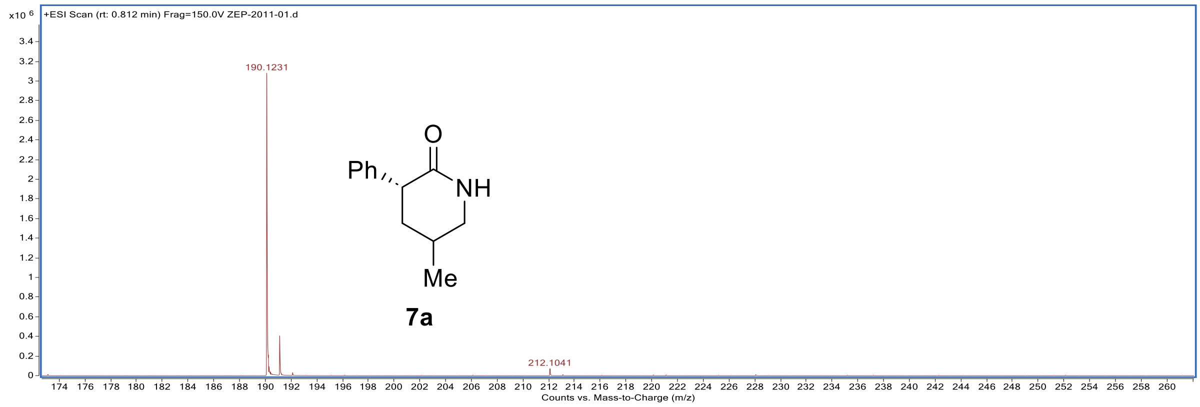

<u>HRMS spectrum</u> <u>of compound **8a**</u>

ESI-QTOF, MeOH, calcd *m/z* for $C_{12}H_{14}NO_2^+$: 204.1019 [*M*+H]$^+$; found: 204.1021.

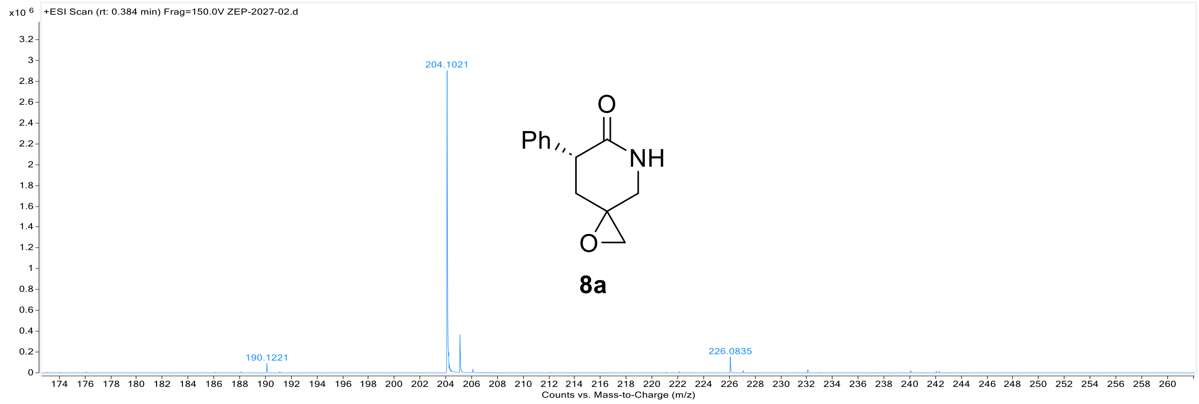

Supplement: Supplementary file 1 — Supporting Information [file EJOC-26-0-s001.pdf]
